# Classification Performance of Deep Learning Models for the Assessment of Vertical Dimension on Lateral Cephalometric Radiographs

**DOI:** 10.3390/diagnostics15172240

**Published:** 2025-09-03

**Authors:** Mehmet Birol Özel, Sultan Büşra Ay Kartbak, Muhammet Çakmak

**Affiliations:** 1 Department of Orthodontics, Faculty of Dentistry, Kocaeli University, Kocaeli 41190, Türkiye; sultanbusraay@gmail.com; 2 Department of Computer Engineering, Faculty of Engineering, Giresun University, Giresun 28200, Türkiye; muhammet.cakmak@giresun.edu.tr

**Keywords:** deep learning, artificial intelligence, cephalometry, vertical discrepancies

## Abstract

**Background/Objectives**: Vertical growth pattern significantly influences facial aesthetics and treatment choices. Lateral cephalograms are routinely used for the evaluation of vertical jaw relationships in orthodontic diagnosis. The aim of this study was to evaluate the performance of deep learning algorithms in classifying cephalometric radiographs according to vertical skeletal growth patterns without the need for anatomical landmark identification. **Methods**: This study was carried out on lateral cephalometric radiographs of 1050 patients. Cephalometric radiographs were divided into 3 subgroups based on FMA, SN-GoGn, and Cant of Occlusal Plane angles. Six deep learning models (ResNet101, DenseNet 201, EfficientNet B0, EfficientNet V2 B0, ConvNetBase, and a hybrid model) were employed for the classification of the dataset. The performances of the well-known deep learning models and the hybrid model were compared for accuracy, precision, F1-Score, mean absolute error, Cohen’s Kappa, and Grad-CAM metrics. **Results**: The highest accuracy rates were achieved by the Hybrid Model with 86.67% for FMA groups, 87.29% for SN-GoGn groups, and 82.71% for Cant of Occlusal Plane groups. The lowest accuracy rates were achieved by ConvNet with 79.58% for FMA groups, 65% for SN-GoGn, and 70.21% for Cant of Occlusal Plane groups. **Conclusions**: The six deep learning algorithms employed demonstrated classification success rates ranging from 65% to 87.29%. The highest classification accuracy was observed in the FMA angle, while the lowest accuracy was recorded for the Cant of the Occlusal Plane angle. The proposed DL algorithms showed potential for direct skeletal orthodontic diagnosis without the need for cephalometric landmark detection steps.

## 1. Introduction

Accurately evaluating a patient’s facial skeletal pattern in three dimensions—transverse, vertical, and sagittal—is crucial for orthodontic diagnosis and treatment planning [1]. Variations in vertical growth are frequent and carry particular orthodontic significance. Abnormalities in hard or soft tissues can cause the face to lengthen or shorten. Insufficient lips, an elongated face, and a gummy smile may occur due to an excessive vertical dimension. On the other hand, insufficient vertical growth can lead to inadequate incisor display and a short face [2].

Assessing the vertical jaw relationship is crucial because the vertical growth pattern influences facial balance and treatment choices, such as the need for anchorage, macro aesthetics, extraction or non-extraction treatment/surgical or non-surgical treatment decision, and treatment time [3]. Since the vertical growth of the face ends last, the evaluation of vertical discrepancies is extremely important not only for correct diagnosis and effective treatment planning, but also to prevent relapse after treatment [4].

Cephalometry is one of the radiographic methods that provides morphological and descriptive diagnostic data about skeletal and dentoalveolar malocclusion [5]. Different angular and linear cephalometric techniques have been utilized to evaluate vertical jaw relationships, each with its own set of advantages and disadvantages. However, there is no single extensively studied method in the literature for determining vertical plane discrepancies, as the vertical plane can be affected by a multitude of factors such as age, gender, type of malocclusion, and mandibular plane angle [3,6].

Artificial intelligence (AI) algorithms have demonstrated their effectiveness across multiple medical fields, often exceeding the abilities of seasoned healthcare professionals. These algorithms facilitate the examination, organization, visualization, and classification of healthcare data [7].

Deep learning (DL), a subset of artificial intelligence, allows computers to learn and make decisions without being explicitly programmed. These algorithms, inspired by the neural networks found in the human brain, have diverse applications across various industries, including healthcare [8]. In the field of computer vision, particularly in image classification tasks, deep convolutional neural networks (CNNs) have emerged as the primary algorithms, achieving state-of-the-art performance in many applications. The strength of CNNs lies in their deep architecture, which enables the extraction of a hierarchy of discriminative features at multiple levels of abstraction [9]. In orthodontics, deep learning-based convolutional neural networks (CNNs) are utilized in a wide range of applications, including the prediction of growth and developmental stages, as well as diagnosis and treatment planning [10].

The integration of AI into clinical orthodontic practice enhanced various aspects of patient care, including diagnosis, treatment planning, evaluation of growth and development, monitoring of treatment progress and outcomes, maintenance phase management, remote monitoring, and long-term follow-up [11]. Additionally, chatbot systems developed based on large language models (LLMs) present substantial potential in the field, facilitating broader and more accessible applications of artificial intelligence due to their ease of access via the internet and their ability to perform a wide range of tasks [10]. AI also offers promising opportunities to enhance patient education and the dissemination of medical information [12].

As AI systems advance, numerous research efforts are focused on automatic landmark identification for cephalometric analyses. While there is no universally accepted gold standard for evaluating vertical discrepancies in cephalometric analysis, most clinical and research applications rely on expert-defined criteria or commonly used reference angles such as FMA, SN-GoGn, and Occlusal Plane Inclination. These reference points serve as practical benchmarks despite variability in their application across studies and institutions. A systematic review and meta-analysis investigated the application of DL for cephalometric landmark detection. Applications like WebCeph, OrthoDx, Ceph-X, AudaxCeph, and WeDoCeph have been created to automate the marking of cephalometric points, analysis, and calculations through the use of artificial intelligence technology [13].

Artificial intelligence (AI) algorithms employed for automated landmark identification on lateral cephalometric radiographs are generally characterized by high accuracy. Across most studies, over 80% of landmarks were detected within a 2 mm confidence interval [14]. Since the diagnosis of abnormalities through cephalometric analysis relies on measurements within a narrow range of millimeters or degrees, even minor errors in landmark localization can lead to significant misclassification [15]. Beyond traditional cephalometric radiographs, several studies have demonstrated that skeletal classifications can also be performed by applying cephalometric analysis directly to photographic images [16,17,18].

The aim of this study was to evaluate the performance of deep learning algorithms in classifying lateral cephalometric radiographs in terms of vertical classifications done using FMA, SN-GoGn, and Occlusal Plane Inclination measurements. Unlike traditional landmark-based diagnostic approaches, the proposed deep learning framework aims to eliminate the dependency on anatomical point identification, which is prone to inter- and intra-observer variability and may introduce cumulative measurement errors. To the best of our knowledge, this is one of the first studies to compare traditional cephalometric angles with landmark-independent deep learning classifiers in the context of vertical discrepancy classification.

The null hypothesis (H_0_) of our study is as follows: Deep learning models do not exhibit comparable diagnostic accuracy with landmark-based diagnostic approaches in terms of vertical cephalometric evaluation.

## 2. Materials and Methods

This is a cross-sectional retrospective study that was carried out in the Department of Orthodontics and Dentofacial Orthopedics, Kocaeli University, after approval from the Institutional Ethics Committee (KU GOKAEK-2025/15/30).

### 2.1. Data Acquisition and Classification

Lateral cephalograms of 1050 subjects were selected for the study from the department’s record archive (mean age = 15.6 years, age range = 8.8 to 47.8 years, 588 females, 462 males). The inclusion criteria were (1) high-quality lateral cephalometric radiographs with fully visible craniofacial landmarks, (2) patients aged 10 to 30 years at the time of imaging, (3) radiographs acquired prior to any orthodontic or surgical intervention, and (4) availability of corresponding vertical cephalometric measurements (FMA, SN-GoGn, and OP angles). Exclusion criteria were (1) radiographs with significant anatomical distortion or motion artifacts, (2) history of craniofacial trauma or syndromic conditions (e.g., cleft lip/palate), (3) incomplete clinical records or missing reference measurements, and (4) presence of orthodontic appliances at the time of imaging. All lateral cephalometric radiographs were taken under standard method with an X Ray device (J. Morita MFG. Corp Veraviewepocs 2D, Kyoto, Japan) with a magnification difference of 1.1 mm as determined by the manufacturer (80 kV, 10 mA, and 7.4 s). Lateral cephalometric radiographs were taken with the patient in neutral head position, teeth in centric occlusion, and lips in a relaxed position.

Webceph AI-based orthodontic and orthognathic online platform (AssembleCircle Corp., Seongnam, Republic of Korea) was used for performing the cephalometric measurements. Digital images of lateral cephalometric radiographs were uploaded to the WebCephTM server. Magnification correction was undertaken based on a known distance of 10 mm between two fixed points on the cephalostat rod in the radiograph. After automatic landmark identification was performed by WebCeph, anatomical landmarks were checked and manually repositioned.

**Figure 1:**
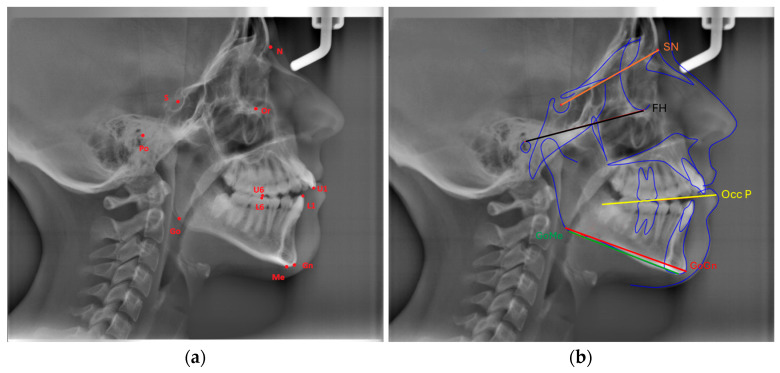
(**a**) Cephalometric landmarks and their definitions: Or (Orbitale): The most inferior point of the orbital floor, Po (Porion): Superior point of the meatus acusticus exter-nus, Go (Gonion): Posteroinferior point of the angulus mandibula formed by the intersection of posterior border of the mandibular ramus and the inferior border of the mandibular corpus, Gn (Gnathion): The most anteroinferior point of the mandibular symphysis between pogonion and menton, Me (Menton): The most inferior point on the mandibular symphysis, S (Sella): The center point of the sella turcica, N (Nasion): The most anterior point on the nasofrontal suture, U6: Tip of the mesiobuccal cusp of upper permanent first molar, U1: Incisal tip of the maxillary central incisor, L6: Tip of the mesiobuccal cusp of lower permanent first molar, L1: Incisal tip of the mandibular central incisor. (**b**) cephalometric measurements.

Eleven landmarks were marked on each cephalogram (Or, Po, Go, Gn, Me, S, N, U6, U1, L6, L1), and the following three measurements were made using three planes formed by these landmarks (Figure 1a,b):

FMA: The angle formed between the Frankfort Horizontal Plane (Or-Po) and the mandibular plane (Go-Me).

SN-GoGn (Mandibular plane angle): The angle between the GoGn and SN lines.

Cant of Occlusal Plane: The angle between the occlusal plane (the line bisecting the overlapping cusps of first molars and incisor overbite) and the Frankfort Horizontal Plane.

To assess the reproducibility of the measurements, the same researcher re-evaluated 50 randomly chosen lateral cephalometric radiographs one week after the completion of all four cephalometric measures. The intraclass correlation coefficient, which measures intraobserver agreement, was found to be between 0.89 to 0.98, indicating strong reproducibility of cephalometric measurements.

Each cephalometric parameter—FMA, SN-GoGn, and Occlusal Plane Inclination—was divided into three subgroups representing vertical skeletal patterns: hypodivergent (low angle), normodivergent (average angle), and hyperdivergent (high angle). These groups were formed based on the numerical distribution of the dataset rather than fixed normative thresholds. Classification intervals were derived by dividing the full range of values into three equal-sized quantiles (see Table 1). This approach ensured statistical balance across subgroups for model training.

### 2.2. Dataset Formation, DL Algorithms, and Training

The dataset used in this study comprised three classes: FMA, SN-GoGn, and the Cant of the occlusal plane. The original dataset contained 350 images for each subclass. To augment the dataset, several image processing techniques were applied, including horizontal translation, rotation, width shift, height shift, and zoom. As a result of these augmentation techniques, the number of images in each subclass was increased to 800. Following the data augmentation process, the dataset was partitioned into training and testing sets, with labeled examples used for all DL models. In all models, 80% of the dataset was allocated for training, while the remaining 20% was designated for testing.

In this study, six DL models were utilized: ResNet101, DenseNet201, EfficientNet B0, EfficientNet V2 B0, ConvNetBase, and a hybrid model.

This study employed widely used convolutional neural network architectures—ResNet101, DenseNet201, EfficientNet B0, EfficientNet V2 B0, and a hybrid model integrating EfficientNet B0 and DenseNet201. These models were selected due to their proven performance in medical image classification tasks and their architectural advantages: ResNet’s residual connections mitigate vanishing gradient problems in deep networks; DenseNet promotes feature reuse through dense connectivity; EfficientNet optimizes accuracy and computational cost via compound scaling; and the proposed hybrid model aims to combine DenseNet’s rich feature extraction with EfficientNet’s parameter efficiency to improve diagnostic precision.

ResNet101 (Residual Network 101) is a deep convolutional neural network comprising 101 layers, designed to address the vanishing gradient problem in very deep networks through the use of skip (residual) connections. As the depth of a neural network increases, training becomes increasingly difficult due to the degradation of gradient flow, which can hinder the update of model parameters. ResNet101 mitigates this issue by enabling direct information transfer between non-adjacent layers, thereby maintaining efficient gradient propagation and facilitating effective learning even in extremely deep architectures.

DenseNet201 (densely connected convolutional network) is a 201-layer deep neural network that introduces dense connectivity between layers. Unlike conventional CNNs, where each layer receives input only from the preceding layer, in DenseNet201, each layer has direct access to the outputs of all preceding layers. This architecture enhances gradient flow, encourages feature reuse, and reduces the number of parameters required, resulting in more efficient learning and improved model performance.

EfficientNet B0 is a convolutional neural network that prioritizes parameter efficiency while maintaining high accuracy. Traditional CNNs often scale model performance by increasing depth or width independently; in contrast, EfficientNet employs a compound scaling method that jointly optimizes network depth, width, and input resolution. This balanced approach enables EfficientNet B0 to achieve superior accuracy with significantly fewer parameters, making it suitable for deployment in resource-constrained environments such as mobile or edge devices.

EfficientNetV2 B0 is an enhanced version of EfficientNet B0, offering faster training and improved performance. It incorporates a progressive learning strategy, wherein data augmentation is gradually increased throughout training. This method accelerates convergence and enhances generalization. EfficientNetV2 B0 achieves higher accuracy while requiring fewer floating-point operations (FLOPs), thereby improving training efficiency and inference speed.

ConvNetBase is a baseline convolutional neural network model built upon the core principles of traditional CNN architectures. It consists of standard convolutional layers, activation functions (typically ReLU), and pooling operations. While it is more lightweight and computationally less demanding than more complex architectures, its performance in terms of accuracy is generally lower. ConvNetBase is often utilized in scenarios involving small datasets or when rapid model training is a priority.

**Figure 2:**
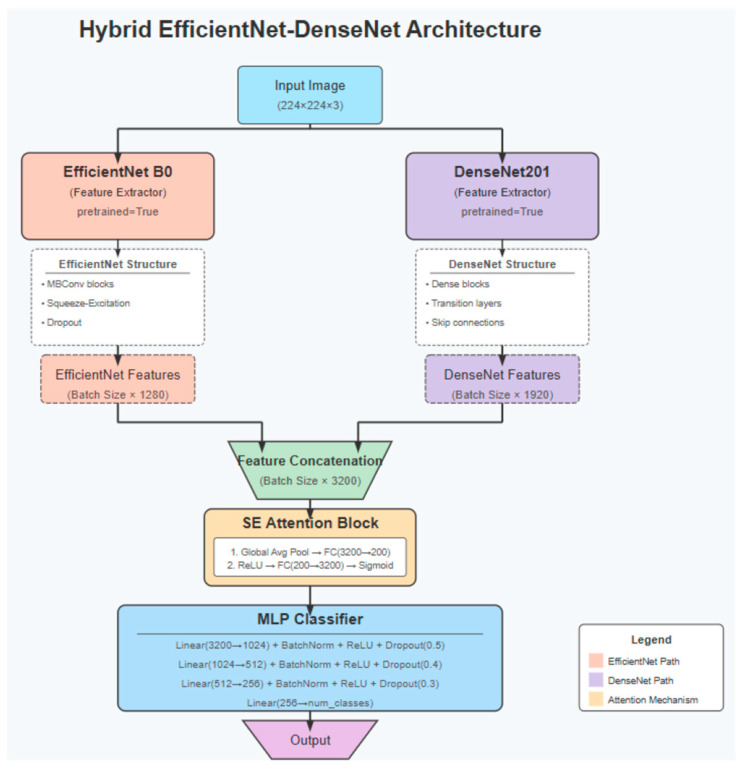
Hybrid model architecture.

The hybrid model, a robust classification architecture, was developed by integrating the EfficientNet B0 and DenseNet201 networks. The architecture processes a 224 × 224 × 3 input image, which was simultaneously fed into two distinct deep learning models. EfficientNet B0, known for its parameter efficiency, optimizes the features extracted from deeper layers, generating a feature vector of size 1280. In contrast, DenseNet201 leverages dense connections to extract richer, more detailed features, resulting in a feature vector of size 1920. These two feature vectors were then merged to form a unified feature space of 3200 dimensions. Subsequently, a squeeze-and-excitation (SE) attention block was applied to enhance the model’s learning by emphasizing critical information. This attention mechanism refines feature selection through channel-based scaling, enabling the model to focus on more informative features. The final stage of the model consists of a multi-layer perceptron (MLP) classifier. The MLP classifier was designed with a series of layers, starting at 1024 units, gradually reducing to 512 and 256 units, before producing the final class prediction. To promote a more stable and regular learning process, several techniques were employed, including batch normalization, ReLU activation, and dropout between the layers (Figure 2).

All deep learning models were trained using a Google Cloud-based system equipped with an NVIDIA Tesla T4 GPU (16 GB VRAM), Intel Xeon CPU (2.20 GHz), and 16 GB RAM. The average training time per model was approximately 3.8 h. Inference time per image was ~85 milliseconds (0.085 s), enabling near-real-time prediction.

Diagnostic accuracy, sensitivity, specificity, the area under the curve (AUC), Cohen’s Kappa metrics among training and test groups, mean absolute error (MAE), and confusion matrices were used to test the performance of DL algorithms among training and test groups.

The Grad-CAM (gradient-weighted class activation mapping) method was used to visualize the decision mechanism of the model and analyze it with explainable artificial intelligence methods.

## 3. Results

The results according to the parameters used in our study are as follows:

### 3.1. FMA

The accuracy, MAE, and Cohen’s Kappa metric values for six deep learning algorithms applied to the FMA parameter are provided in Table 2.

The highest accuracy rate for FMA was obtained by the hybrid model (86.67%), with 416 correct classifications out of 480 images. ConvNetBase presented the lowest accuracy rate, at 79.58%, with correct classifications for 382 images out of 480. Evaluation using the MAE and Cohen’s Kappa metrics indicates that the hybrid model is the most reliable among the models assessed.

**Figure 3:**
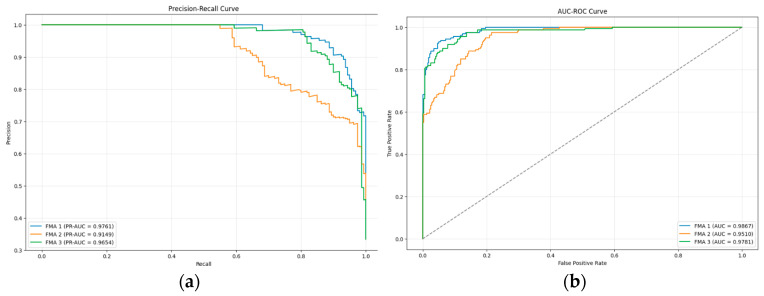
(**a**) The precision–recall curve of FMA; (**b**) AUC-ROC curve of FMA.

The precision–recall curve and the AUC-ROC curve for FMA using the hybrid model are presented in Figure 3. The lowest precision–recall value across the three subgroups was 0.9149, while the lowest AUC value recorded was 0.9510.

**Figure 4:**
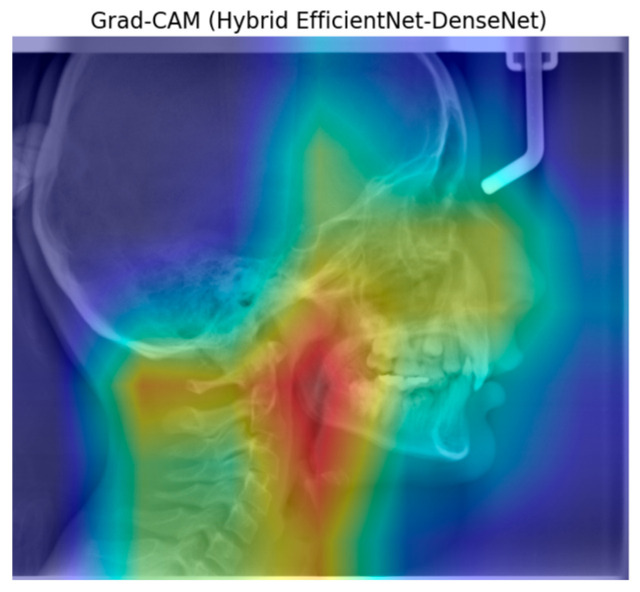
Grad-CAM of the hybrid model for FMA.

Figure 4 shows an example of heatmap images reflecting the characteristics of the learning and classification behavior of the hybrid model for FMA.

### 3.2. SN-GoGn

The accuracy, MAE, and Cohen’s Kappa metric values for six deep learning algorithms applied to the SN-GoGn parameter are provided in Table 3.

The highest accuracy rate for SN-GoGn was obtained by the hybrid model (87.29%), with 419 correct classifications out of 480 images. ConvNetBase presented the lowest accuracy rate, at 65%, with correct classifications for 312 images out of 480. Evaluation using the MAE and Cohen’s Kappa metrics indicates that the hybrid model is the most reliable among the models assessed.

**Figure 5:**
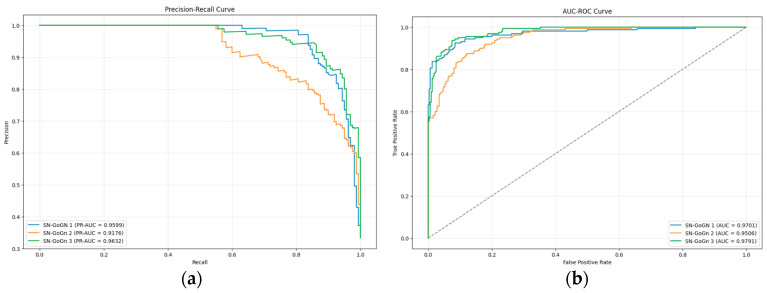
(**a**) The precision–recall curve of SN-GoGn; (**b**) AUC-ROC curve of SN-GoGn.

The precision–recall curve and the AUC-ROC curve for SN-GoGn using the hybrid model are presented in Figure 5. The lowest precision–recall value across the three subgroups was 0.9176, while the lowest AUC value recorded was 0.9506.

**Figure 6:**
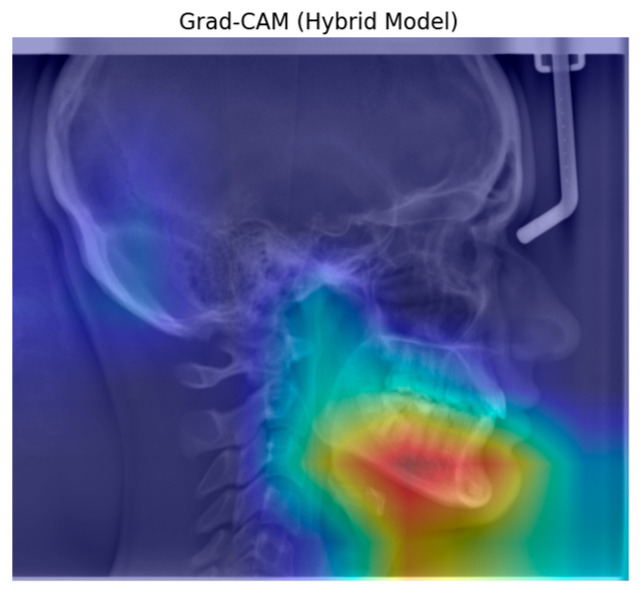
Grad-CAM of the hybrid model for SN-GoGn.

Figure 6 shows an example of heatmap images reflecting the characteristics of the learning and classification behavior of the hybrid model for SN-GoGn.

### 3.3. Cant of Occlusal Plane

The accuracy, MAE, and Cohen’s Kappa metric values for six deep learning algorithms applied to the Cant of the Occlusal Plane parameter are provided in Table 4.

The highest accuracy rate for the Cant of Occlusal Plane was obtained by the Hybrid model (82.71%), with 397 correct classifications out of 480 images. ConvNetBase presented the lowest accuracy rate, at 70.21%, with correct classifications for 337 images out of 480. Evaluation using the MAE and Cohen’s Kappa metrics indicates that the hybrid model is the most reliable among the models assessed.

**Figure 7:**
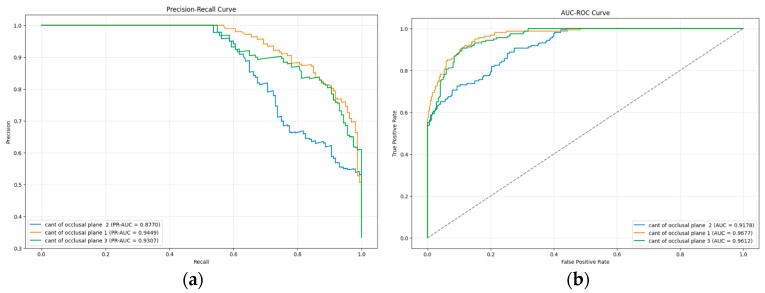
(**a**) The precision–recall curve of Cant of the Occlusal Plane; (**b**) AUC-ROC curve of Cant of the Occlusal Plane.

The precision–recall curve and the AUC-ROC curve for the Cant of the Occlusal Plane using the hybrid model are presented in Figure 7. The lowest precision–recall value across the three subgroups was 0.8770, while the lowest AUC value recorded was 0.9178.

**Figure 8:**
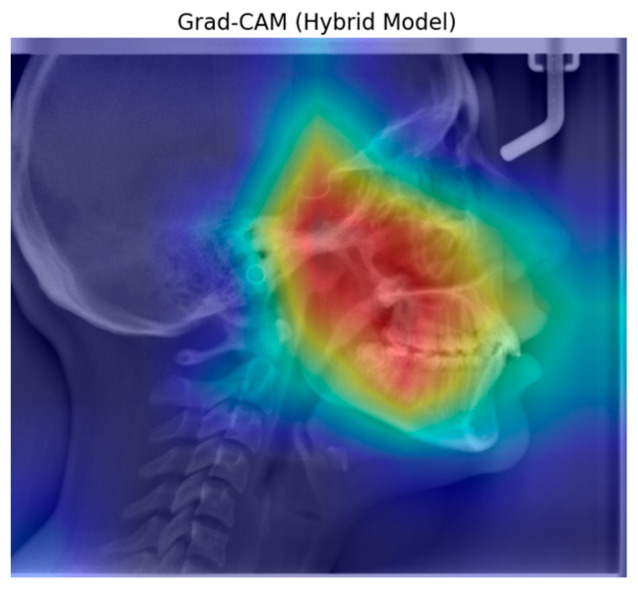
Grad-CAM of the hybrid model for the Cant of the Occlusal Plane.

Figure 8 shows an example of heatmap images reflecting the characteristics of the learning and classification behavior of the hybrid model for the Cant of the Occlusal Plane.

### 3.4. Visualization of Localization Results

**Figure 9:**
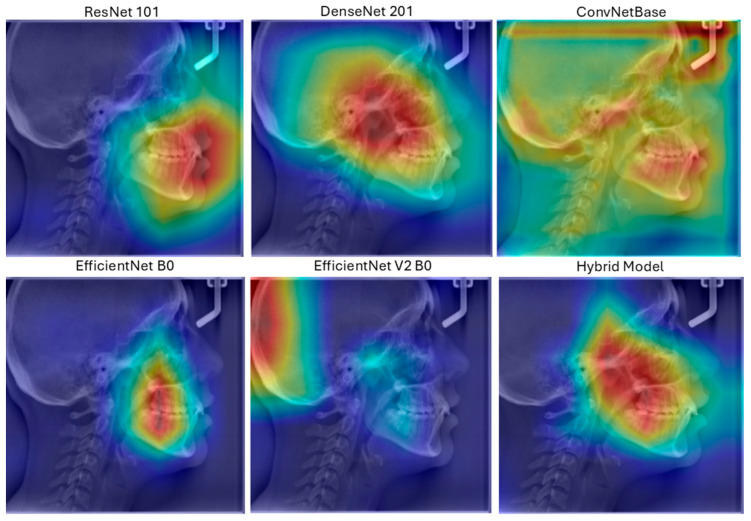
Grad-CAM analysis of DL models used in this study.

Grad-CAM analysis of ResNet101, DenseNet201, ConvNetBase, EfficientNet B0, EfficientNet V2 B0, and the hybrid model is given in Figure 9.

The Grad-CAM visualization of the ResNet101 architecture reveals that it typically focuses on the central features of the object, although the focus area is relatively dispersed. In comparison, the DenseNet architecture exhibits a more concentrated activation region. The ConvNetBase model shows a wider and less precise activation area compared to the other architectures. EfficientNet B0 demonstrates a more balanced focus on the object’s characteristic features. The improved version, EfficientNet V2, produces more focused and less noisy activation regions than EfficientNet B0. As observed in the Grad-CAM visualizations, the hybrid model generates more accurate, concentrated, and meaningful activation regions than the others.

The whole confusion matrices, tables, and graphs of the DL models used in this study are included in the Supplementary Data.

## 4. Discussion

Among orthodontic malocclusions, vertical discrepancies are one of the most challenging to diagnose and treat due to their complex nature. The interaction and involvement of different contributing elements and their subsequent effects on the treatment approaches and results become an important priority compared to other types of malocclusions [19].

Numerous cephalometric measurements are employed in the evaluation of vertical discrepancies. For instance, the FMA angle is utilized in Tweed analysis; however, accurately determining the Frankfort Horizontal Plane is not always feasible. In Steiner analysis, the SN-GoGn angle is used to assess the vertical direction. However, the SN plane can be influenced by the inclination of the anterior cranial base, which may reduce its reliability in effectively evaluating growth patterns. Research suggests that SN-GoGn and FMA angles can be measured with greater precision in hyperdivergent and normodivergent patients [2]. Given that there is no single correct parameter for vertical direction assessment, our study incorporated three different parameters, and the classification successes among these parameters were compared and evaluated.

In our study, the classification performance of three different vertical parameters was compared using six different DL algorithms. The highest classification success rates were achieved for the SN-GoGn group, with 87.29%, for the FMA group with 86.67%, and for the Cant of Occlusal Plane group with 82.71% by the hybrid model. While all groups yielded promising classification results, the Cant of the Occlusal Plane group demonstrated relatively lower performance.

AI refers to the ability of a machine to mimic human intelligence in performing specific tasks. In recent years, significant advancements have been made in the field of artificial intelligence. Its applications have also increasingly been integrated into the medical field. In particular, digital radiographs offer a valuable resource for artificial intelligence in both medicine and dentistry. These images can be digitally encoded and easily translated into computational language, making them ideal for creating large datasets that can be analyzed using AI technologies [20]. As in our study, there are many studies in the literature in which deep learning algorithms use cephalometric radiographs as a dataset. Automated anatomical landmark detection, cervical vertebral maturation assessment, evaluation of extraction needs in orthodontic treatments, or determination of the necessity for orthognathic surgery, among other evaluations, have achieved successful results using deep learning algorithms on cephalometric radiographs [21,22,23,24,25,26].

In recent years, numerous traditional machine learning and deep learning methods have been developed for cephalometric analysis [27]. Recent studies have predominantly focused on unimodal deep learning approaches, which primarily utilize convolutional neural networks (CNNs) to detect anatomical landmarks from lateral cephalograms [28]. High performance has been reported in recent efforts aimed at the accurate identification of these landmarks. For instance, Lee et al. reported a successful detection rate of 82.11%; Zeng et al. reported 81.37% and 70.58% in two different tests; Uğurlu et al. reported 76.2%; and Yao et al. reported 97.3% [29,30,31,32]. One of the major limitations in training and testing deep learning models for the cephalometric analysis of radiographs is the complexity of data annotation. There is no single ground-truth localization for a given anthropometric landmark that can be universally accepted as a gold standard. Typically, multiple experts manually annotate a particular landmark, and the aggregated results are used to generate the reference labels for training deep learning algorithms [14]. Our study differs from these anatomical landmark detection approaches in that it does not require the detection of cephalometric landmarks. This characteristic offers a significant advantage by mitigating potential misclassifications that may arise due to incorrectly labeled landmarks. As such, our method addresses one of the key challenges in current deep learning-based cephalometric analysis.

In the present study, deep learning models were evaluated using vertical direction parameters. Comparable research has been carried out as well, though concentrating on the assessment of sagittal direction parameters. In the sagittal skeletal classification study conducted by Nan L. et al., the classification performance of the DenseNet algorithm was evaluated based on ANB and Wits measurements from cephalometric radiographs. The study reported an accuracy of 90.33% [18]. Zahng JN et al. reported that they achieved 85% accuracy in their study using a deep learning-based convolutional neural network (CNN) model to predict the growth trend of the mandible of a child with anterior crossbite from pretreatment cephalometric radiographs [33].

Similar to our study, Yu HJ et al. researched automatic skeletal classification using lateral cephalometric radiographs with deep learning, as they classified the vertical direction assessment based on the Björk sum and Jarabak ratio. They achieved 96.40% classification accuracy in this study using the Densenet algorithm. They reported that the classification success of hyperdivergent and hypodivergent groups showed a higher rate than the normal group [34]. Similarly, in our study, we observed that the classification accuracy of hyperdivergent and hypodivergent patients was higher.

Kocakaya DNC et al., in their study evaluating the classification performance of profile photographs using deep learning algorithms, reported achieving a classification accuracy of 97% based on the FMA angle [16]. The differing success rates between the photographs and cephalometric radiographs used as the dataset for the same parameter suggest the need for further research.

ConvNet was the model with the lowest classification accuracy in our study. To the best of our knowledge, ConvNet has not been applied to cephalometric radiographs; however, it has been applied to other radiographic data in certain research. He et al. employed ConvNet, DenseNet, and ResNet architectures for the detection of abnormalities in musculoskeletal radiographs, reporting that ConvNet achieved the lowest classification accuracy at 82%, while DenseNet demonstrated the highest accuracy at 90% [35]. Similar to this, Showkhatian et al. evaluated the architectures of ConvNet, DenseNet, VGG16, VGG19, ResNet50, Exception, and Inception V3 on chest radiographs for the detection of tuberculosis. They discovered that ConvNet had the lowest classification accuracy (87%), while Exception, ResNet50, and VGG16 performed the best (90%) [36].

From a methodological standpoint, landmark-based classification relies on first detecting anatomically defined reference points, followed by deterministic geometric calculations. This pipeline offers high diagnostic transparency, as the decision path is grounded in established cephalometric measurements. However, it is susceptible to cumulative error propagation from landmark misplacement, and it typically requires human oversight or post hoc verification, potentially slowing clinical workflows. By contrast, the end-to-end deep learning model proposed in this study eliminates the need for manual or automatic landmark annotation and learns discriminative features directly from raw radiographic input. This enhances classification speed and simplifies deployment, especially in high-throughput or chairside environments. Yet, it reduces interpretability for clinicians accustomed to traditional cephalometric angles and may obscure the basis for individual predictions unless explainable AI techniques are used. In practice, a hybrid strategy may be ideal—leveraging end-to-end models for initial screening while preserving landmark-based tools for transparent clinical auditing. Future research should explore how these approaches can be integrated seamlessly within digital orthodontic workflows.

The proposed landmark-independent deep learning model offers several practical advantages in orthodontic clinical settings. First, by eliminating the need for manual or automated landmark identification, the model significantly reduces the time required for cephalometric analysis—making real-time chairside diagnosis feasible. Second, it mitigates inter- and intra-examiner variability commonly associated with landmark placement, thereby increasing diagnostic consistency. Third, early and reliable classification of vertical skeletal growth patterns facilitates timely intervention planning, such as growth modification therapies in hyperdivergent patients or vertical control strategies in hypodivergent cases. Finally, the model can be integrated into digital orthodontic platforms to support automated pre-screening and triage, improving overall clinic efficiency.

Grad-CAM is a technique used to visualize the interpretability of models and can easily be applied to various CNN architectures for better understanding [37]. In our study, the hybrid model presents a unique approach by combining the strengths of different architectures. As observed from the Grad-CAM visualizations, this model produces activation maps that are more accurate, focused, and meaningful compared to others. This enhances the model’s performance in classification tasks. In contrast to other models, where activations are scattered across irrelevant regions, the hybrid model’s activation map concentrates more consistently on semantically significant parts of the object.

Recent advancements in artificial intelligence have expanded beyond image classification to include more complex analytical methods such as AI-assisted chaotic functional analysis. This approach leverages nonlinear dynamics and fractal-based pattern recognition to model the complex biological processes underlying craniofacial growth [38]. Integrating metaheuristic optimization techniques into our deep learning framework, where direct image-based classification is complemented by AI-driven chaotic functional models, may have potential for providing a comprehensive tool for individualized orthodontic diagnosis and treatment planning.

There are some limitations to our study. First of all, since this study was conducted at a single center, there is no data that may affect facial features belonging to different ethnic groups. In a multicenter study, the inclusion of radiographic data obtained with different cephalometric devices may allow our findings to be developed more comprehensively. DL algorithms may show more accurate classification performance by increasing the dataset. Although training the proposed models requires GPU acceleration, the final inference phase, once the model is deployed, can be executed on a mid-range CPU-based system with acceptable latency (<0.1 s/image). Therefore, it is technically feasible to implement the model on chair-side computers in clinical environments. However, for large-scale processing or training on new datasets, GPU or cloud resources remain preferable. While our end-to-end classification framework bypasses the landmark annotation step, future work may explore a comparative approach involving CNN-based landmark detection followed by angle-based geometric classification using FMA, SN-GoGn, and Occlusal Plane Inclination. This would allow for a direct evaluation of the performance and practicality trade-offs between fully automated image-to-class networks and semi-automated landmark-dependent systems.

The fact that only vertical parameters were examined in our study is one of the limitations of the study, since other factors that may affect these parameters were excluded. Finally, the use of a dataset consisting of 3D images instead of 2D images may contribute to obtaining more clinically valuable and comprehensive results.

### Strengths of the Study

This study presents several methodological and practical strengths: (1) The use of a novel hybrid deep learning architecture combining EfficientNet and DenseNet allows the model to benefit from both efficient scaling and dense feature propagation, resulting in robust classification performance. (2) Advanced data augmentation techniques, including rotation, translation, and brightness normalization, were applied to improve generalization and mitigate overfitting. (3) The inclusion of Grad-CAM–based visualization enhances the interpretability of the model by highlighting the anatomical regions that contribute most to the classification, promoting clinical trust in AI-generated decisions. (4) The model was trained and validated on a balanced and expertly annotated dataset with clearly defined vertical skeletal pattern categories, ensuring both clinical relevance and methodological rigor.

Our results support the alternative hypothesis (H_1_): deep learning models—especially the proposed hybrid architecture—can classify vertical skeletal patterns from cephalometric radiographs with high accuracy, surpassing the constraints of traditional landmark-dependent methods. The landmark-free pipeline not only improves diagnostic efficiency but also minimizes error propagation, thereby offering a practical tool for chairside clinical implementation.

## 5. Conclusions

This study demonstrates the feasibility of using deep learning models, specifically a hybrid CNN architecture, to classify vertical skeletal growth patterns directly from lateral cephalometric radiographs without relying on anatomical landmarks. Achieving high diagnostic performance and a time-efficient solution through bypassing manual or automated landmark detection, our findings support the integration of end-to-end DL models into clinical orthodontic assessment systems, with potential for real-time chairside implementation.

## Figures and Tables

**Table 1:** Classification values of cephalometric measurements.

Class	Subclass	Classification Value Range
FMA	FMA 1	8.1–22.49
	FMA 2	22.51–27.79
	FMA 3	27.8–46.68
SN-GoGn	SN-GoGn1	11.81–28.08
	SN-GoGn 2	28.14–33.29
	SN-GoGn 3	33.34–57.09
Cant of Occlusal Plane	Cant of Occlusal Plane 1	−6.97–6.64
	Cant of Occlusal Plane 2	6.65–10.63
	Cant of Occlusal Plane 3	10.7–20.64

**Table 2:** Model performance metrics of FMA.

Model	Accuracy	MAE	Cohen’s Kappa
ResNet101	0.8271	0.1958	0.7406
DenseNet201	0.8375	0.1708	0.7562
EfficientNetV2 B0	0.8167	0.1938	0.725
ConvNetBase	0.7958	0.2313	0.6937
EfficientNet B0	0.8375	0.1688	0.7562
Hybrid	0.8667	0.1354	0.8000

**Table 3:** Model performance metrics of SN-GoGn.

Model	Accuracy	MAE	Cohen’s Kappa
ResNet101	0.8042	0.225	0.7063
DenseNet201	0.8208	0.1917	0.7312
EfficientNetV2 B0	0.7917	0.2125	0.6875
ConvNetBase	0.65	0.5021	0.475
EfficientNet B0	0.8583	0.1417	0.7875
Hybrid	0.8729	0.1375	0.8094

**Table 4:** Model performance metrics of the Cant of the Occlusal Plane.

Model	Accuracy	MAE	Cohen’s Kappa
ResNet101	0.7625	0.3521	0.6438
DenseNet201	0.75	0.3688	0.625
EfficientNetV2 B0	0.775	0.3333	0.6625
ConvNetBase	0.7021	0.4104	0.5531
EfficientNet B0	0.7917	0.2979	0.6875
Hybrid	0.8271	0.2521	0.7406

## Data Availability

The raw data supporting the conclusions of this article will be made available by the authors upon request. The Python (version 3.10.12) and TensorFlow (version 2.12.0) code used to implement and evaluate the deep learning models will be made publicly available on GitHub upon publication of this article.

## Supplementary Materials

The following supporting information can be downloaded at: https://www.mdpi.com/article/10.3390/diagnostics15172240/s1.

## Abbreviations

The following abbreviations are used in this manuscript:

AI	Artificial intelligence
DL	Deep learning
MAE	Main absolute error
AUC	Area under the curve
CNN	Convolutional neural network
