# Supplementary material for: Classification Performance of Deep Learning Models for the Assessment of Vertical Dimension on Lateral Cephalometric Radiographs
Source: Diagnostics (Basel). 2025 Sep 3;15(17):2240. doi: 10.3390/diagnostics15172240 (PMC12428445; doi:10.3390/diagnostics15172240)

1.1 Classification of Cant of Occlusal Plane by ConvNet

Figure S1. 1 Training and Testing Loss and Training and Testing Accuracy Graphs for ConvNet

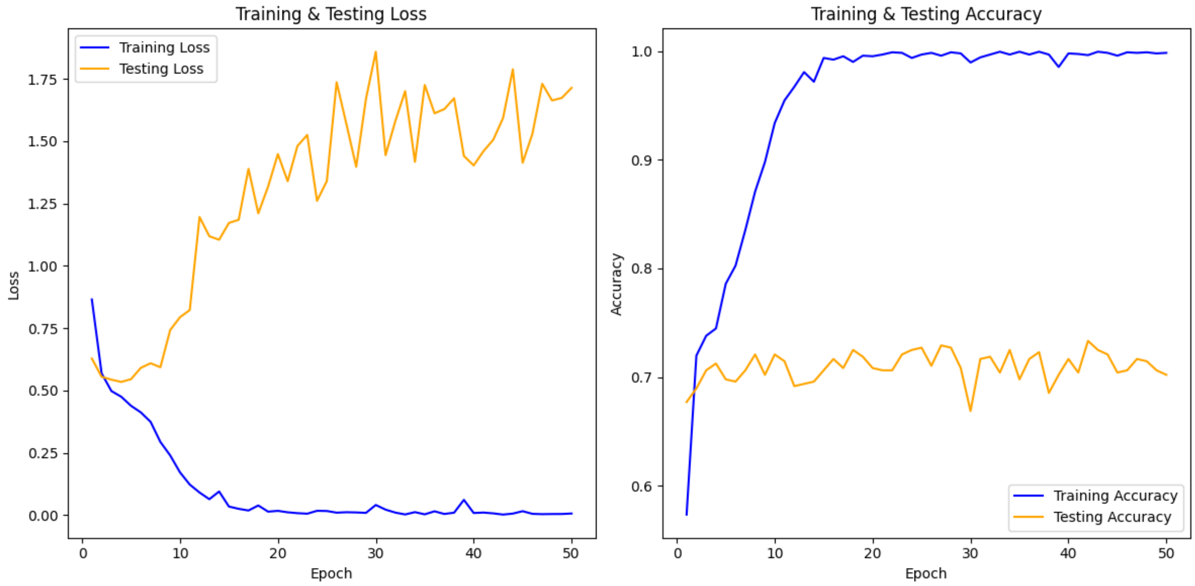

Figure S1. 2 Confusion Matrix for Actual and Predicted Cant of Occlusal Plane values classified by ConvNet

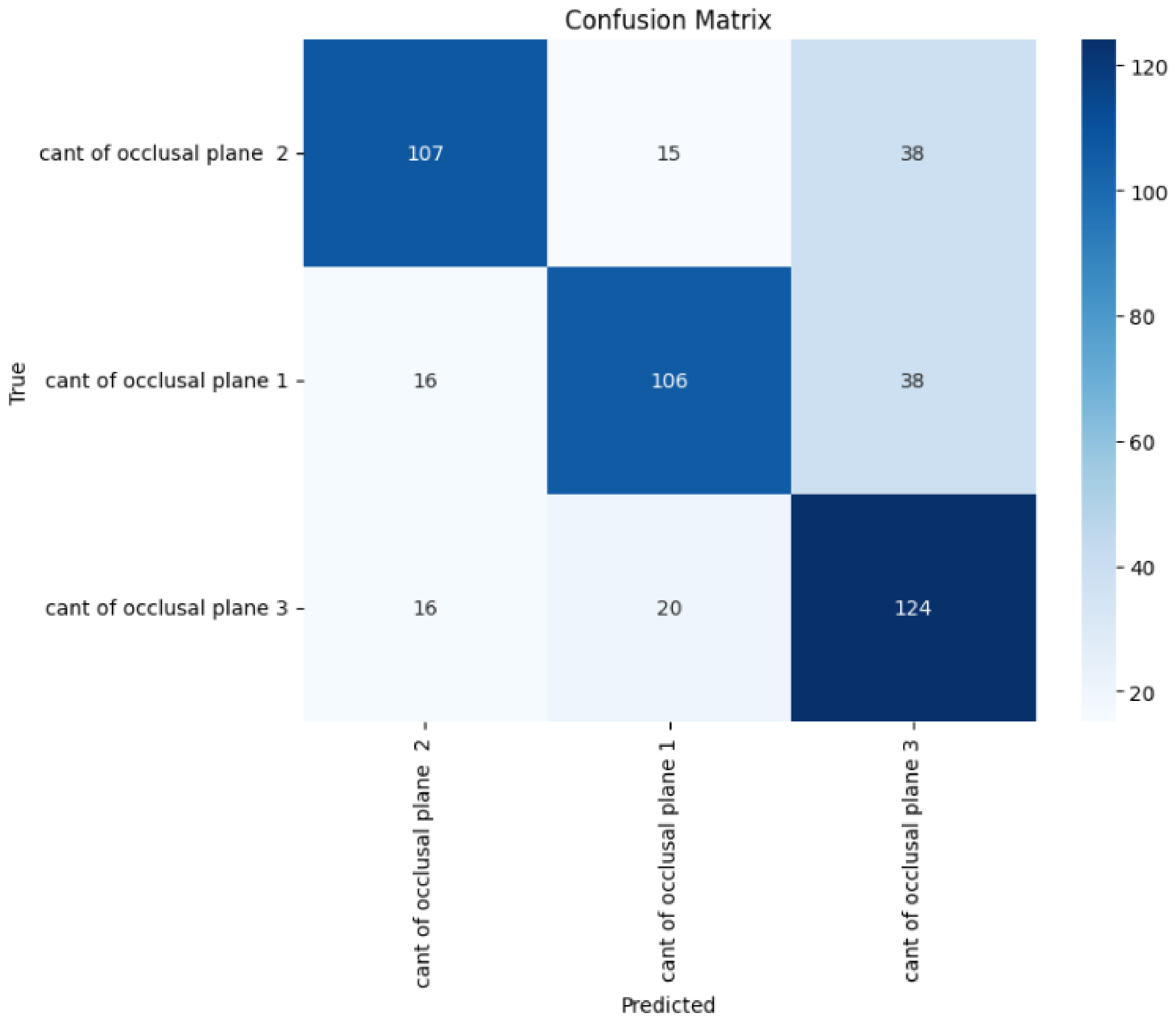

Figure S1. 3 AUC-ROC curve for Cant of Occlusal Plane classified by ConvNet

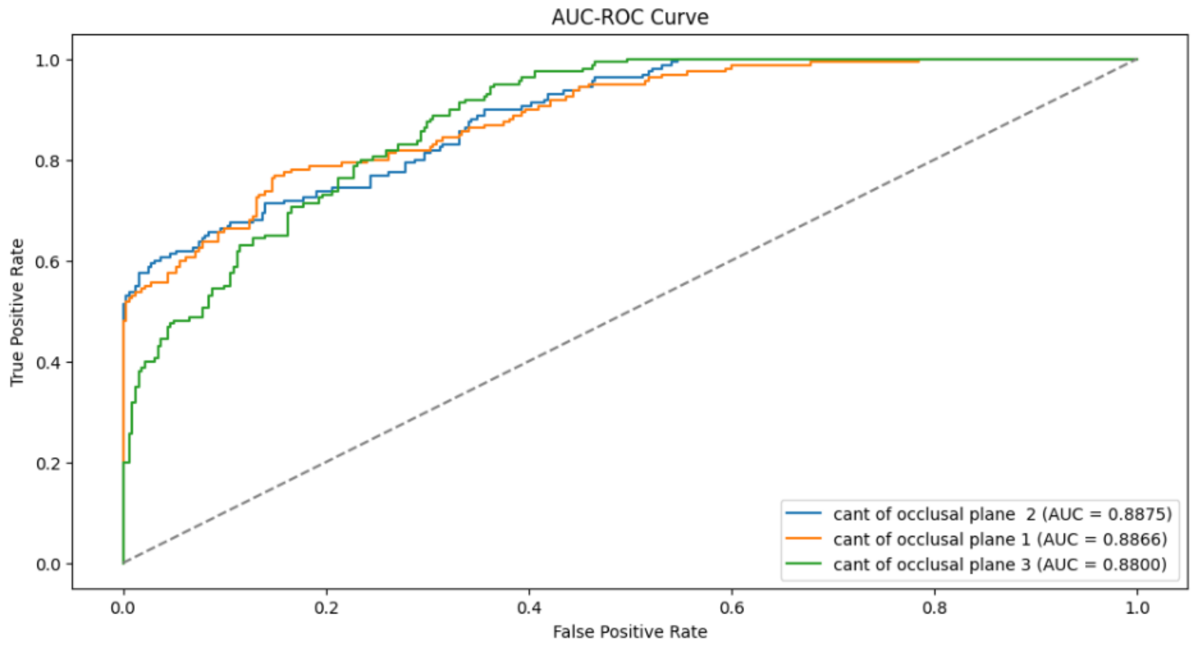

Figure S1. 4 Precision–recall curve for Cant of Occlusal Plane classified by ConvNet

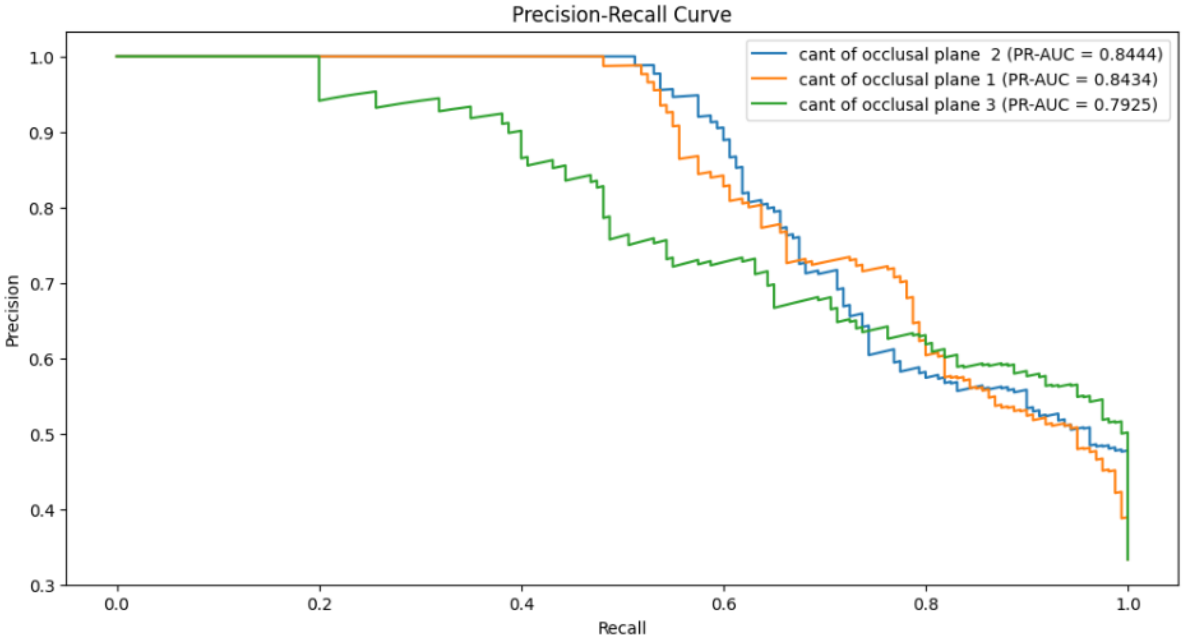

Table S1. 1 Classification Report for Cant of Occlusal Plane by ConvNet

Mean Absolute Error (MAE): 0.4104
Cohen's Kappa: 0.5531
Classification Report:

	precision	recall	f1-score	support
cant of occlusal plane 2	0.7698	0.6687	0.7157	160
cant of occlusal plane 1	0.7518	0.6625	0.7043	160
cant of occlusal plane 3	0.6200	0.7750	0.6889	160
accuracy			0.7021	480
macro avg	0.7139	0.7021	0.7030	480
weighted avg	0.7139	0.7021	0.7030	480

Figure S1. 5 The original and Grad-CAM Images for Cant of Occlusal Plane Generated by ConvNet

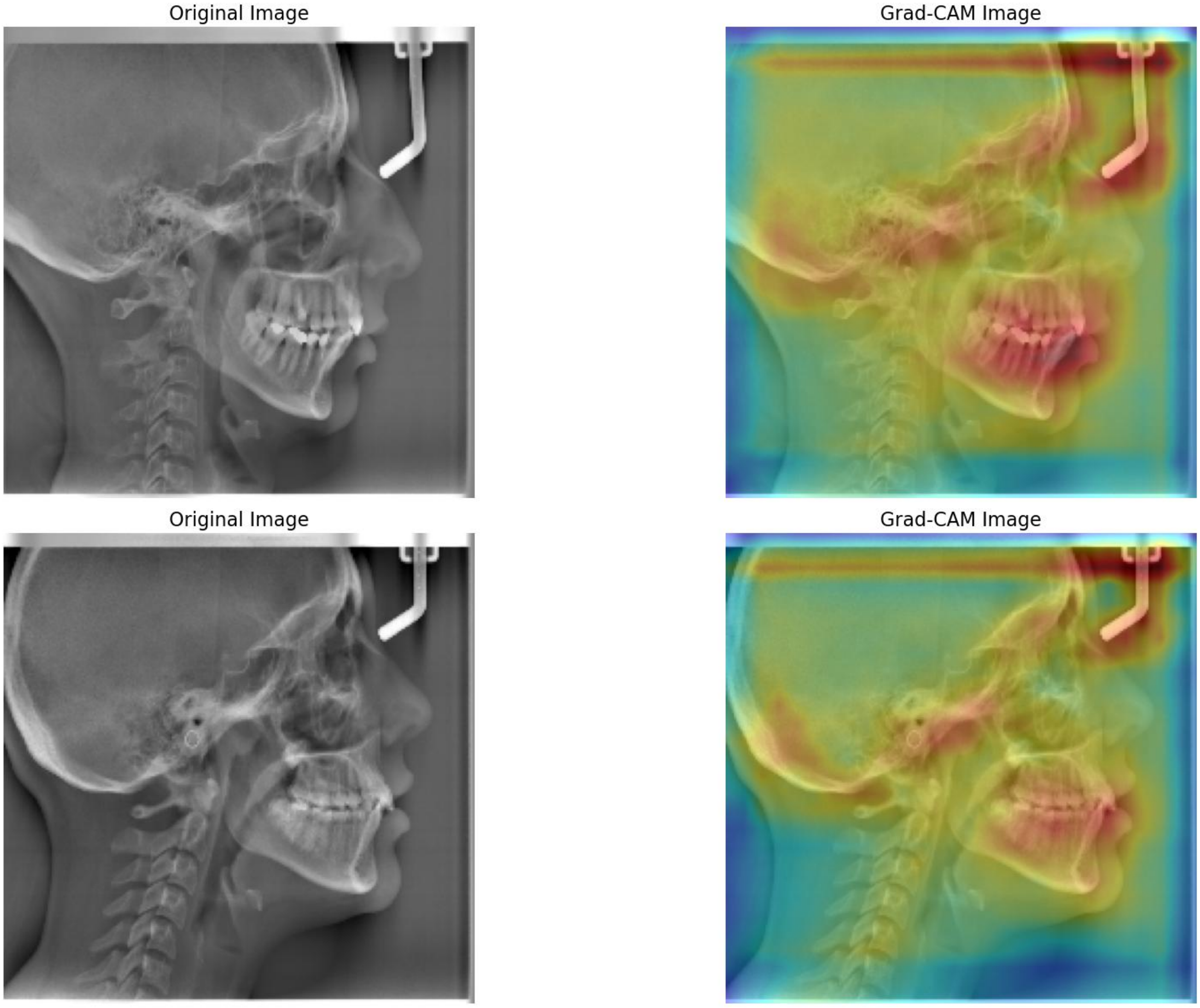

1.2 Classification of Cant of Occlusal Plane by DenseNet201

Figure S1. 6 Training and Testing Loss and Training and Testing Accuracy Graphs for DenseNet201

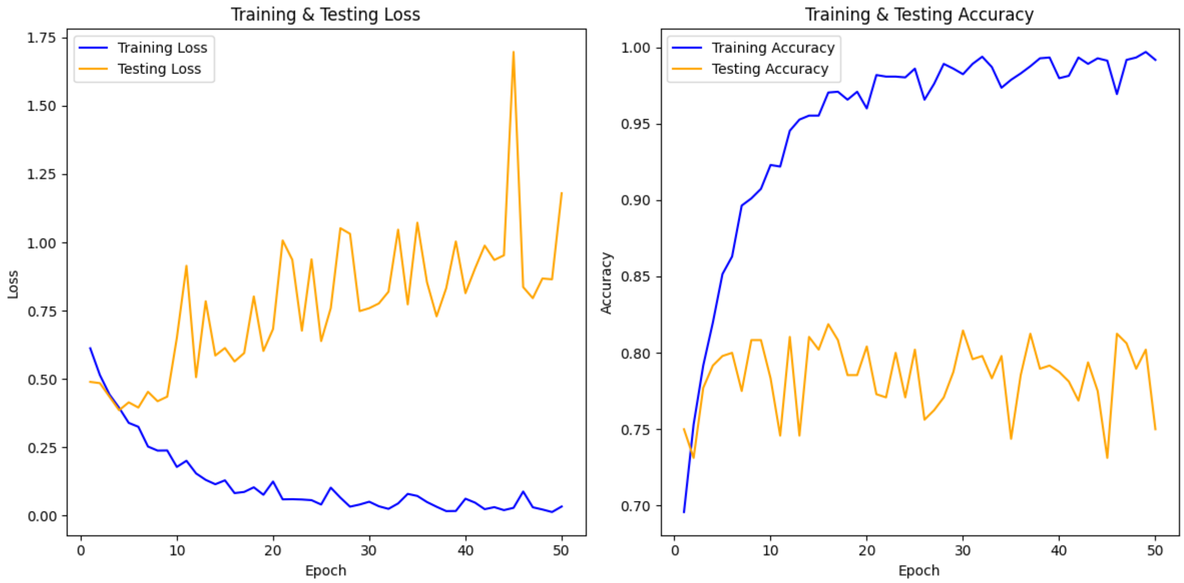

Figure S1. 7 Confusion Matrix for Actual and Predicted Cant of Occlusal Plane values classified by DenseNet201

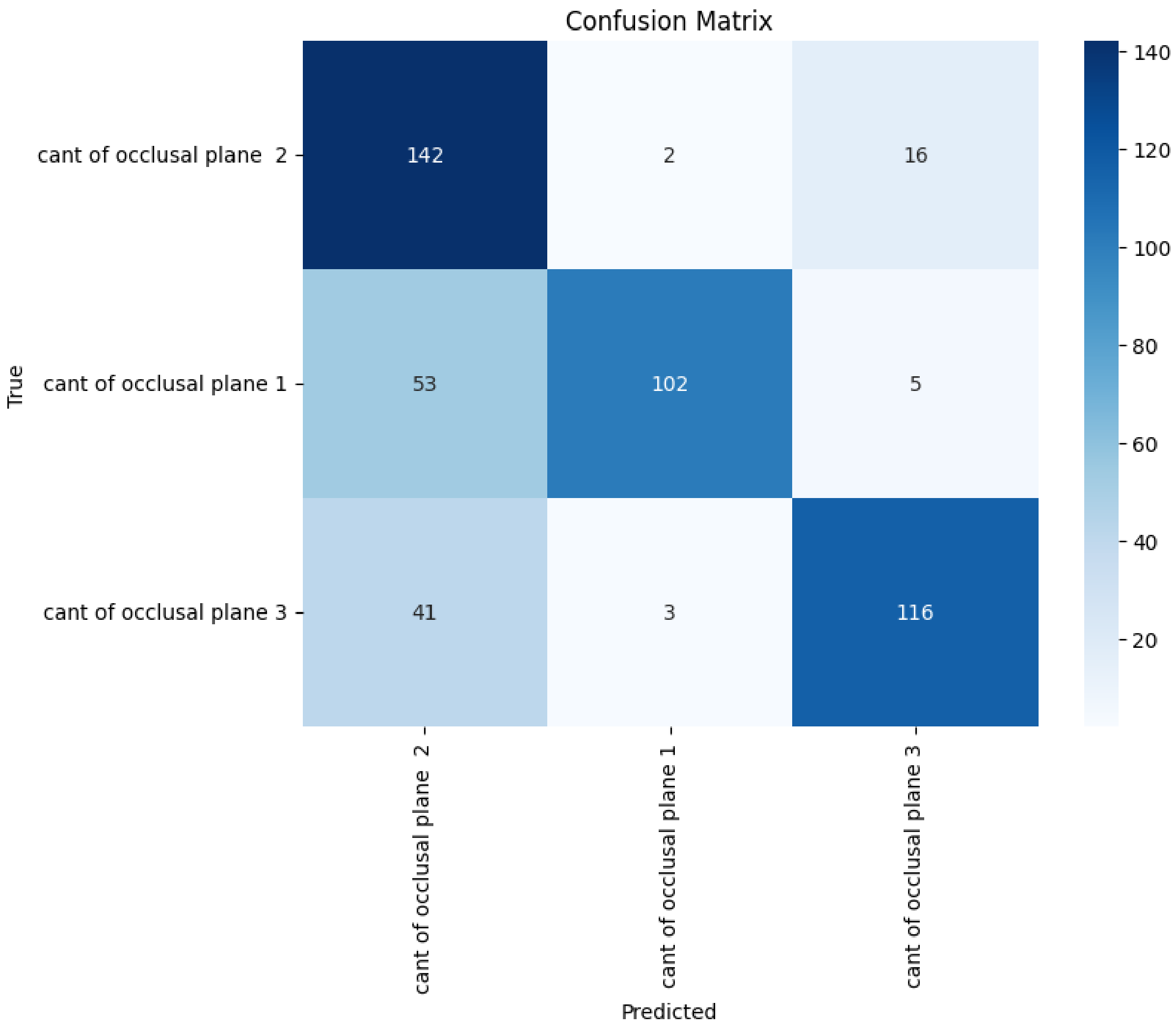

Figure S1. 8 AUC-ROC curve for Cant of Occlusal Plane classified by DenseNet201

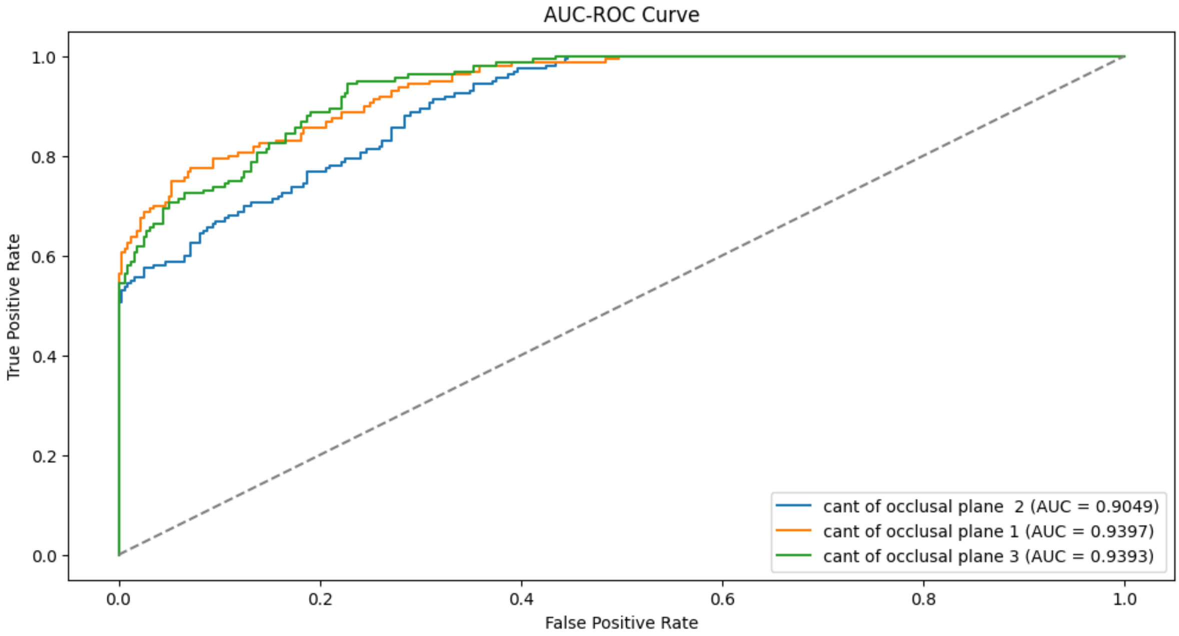

Figure S1. 9 Precision–recall curve for Cant of Occlusal Plane classified by DenseNet201

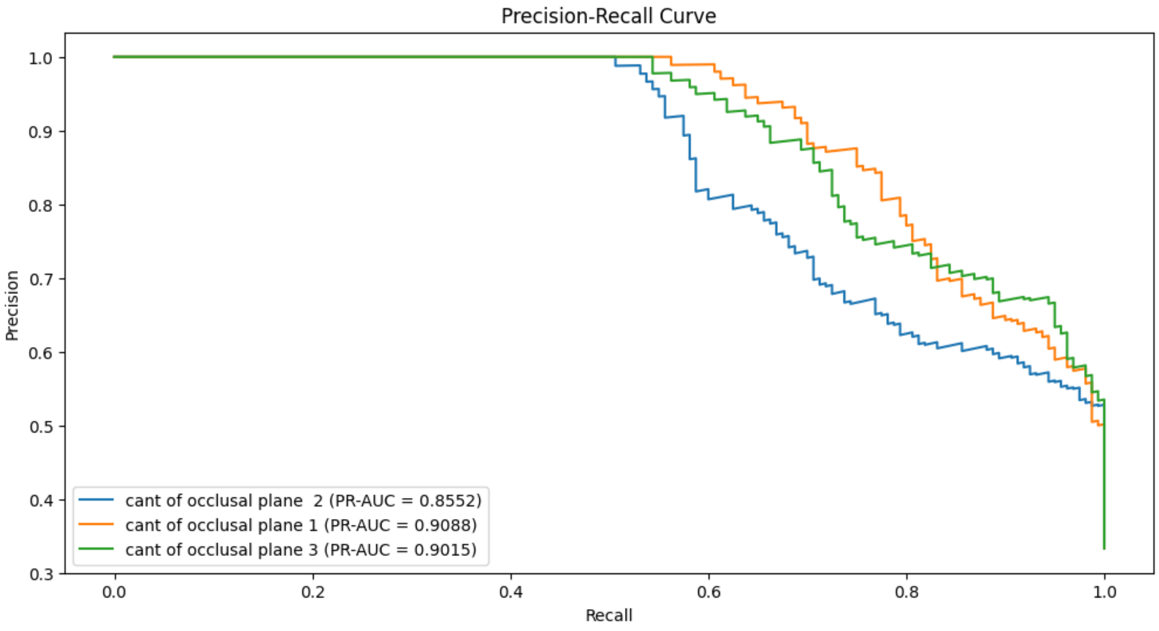

Table S1. 2 Classification Report for Cant of Occlusal Plane by DenseNet201

Mean Absolute Error (MAE): 0.3688
Cohen's Kappa: 0.6250
Classification Report:

	precision	recall	f1-score	support
cant of occlusal plane 2	0.6017	0.8875	0.7172	160
cant of occlusal plane 1	0.9533	0.6375	0.7640	160
cant of occlusal plane 3	0.8467	0.7250	0.7811	160
accuracy			0.7500	480
macro avg	0.8006	0.7500	0.7541	480
weighted avg	0.8006	0.7500	0.7541	480

Figure S1. 10 The original and Grad-CAM Images for Cant of Occlusal Plane Generated by DenseNet201

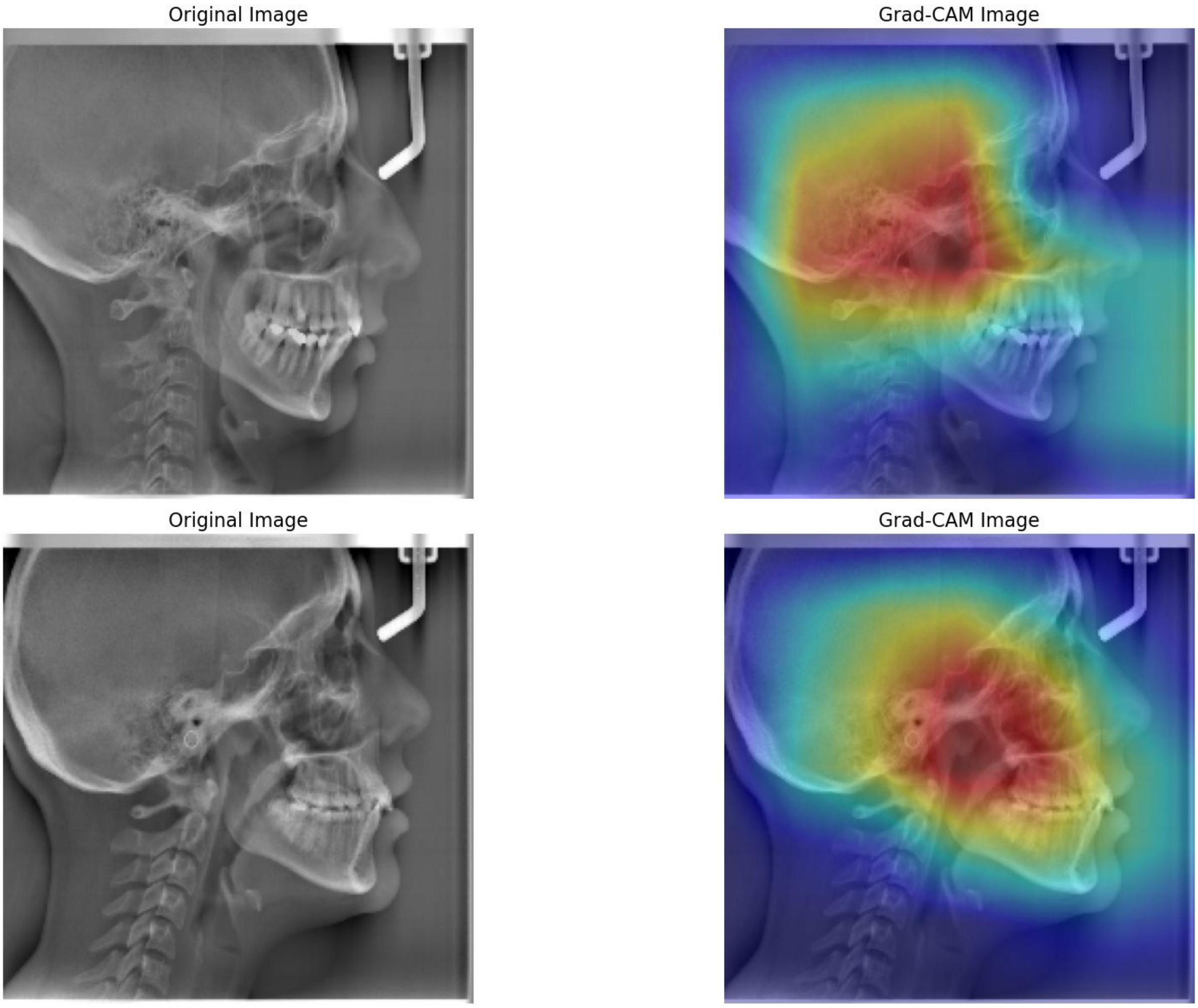

1.3 Classification of Cant of Occlusal Plane by EfficientNet B0

Figure S1. 11 Training and Testing Loss and Training and Testing Accuracy Graphs for EfficientNet B0

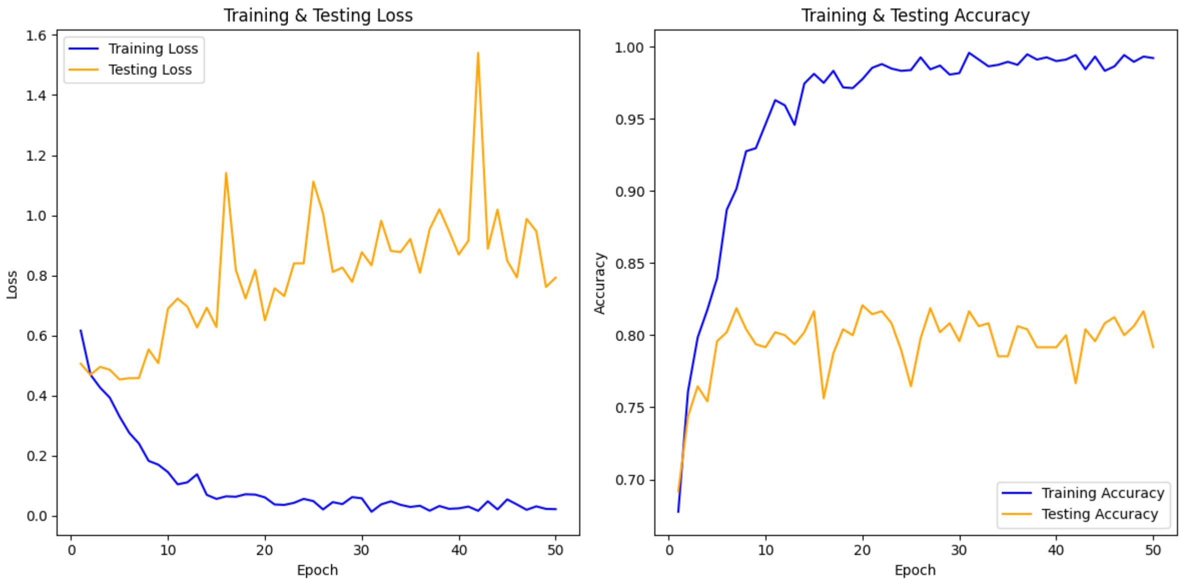

Figure S1. 12 Confusion Matrix for Actual and Predicted Cant of Occlusal Plane values classified by EfficientNet B0

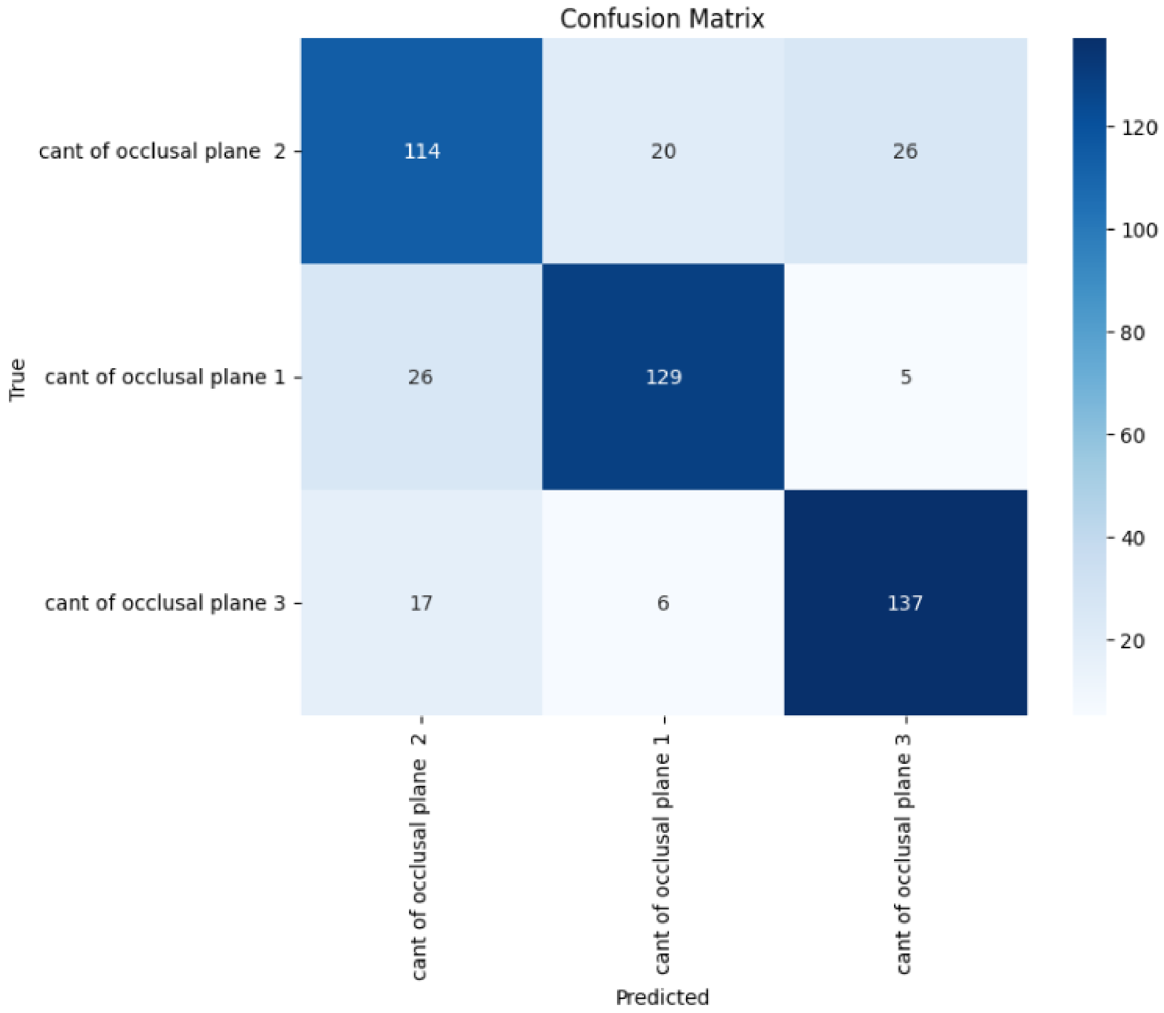

Figure S1. 13 AUC-ROC curve for Cant of Occlusal Plane classified by EfficientNet B0

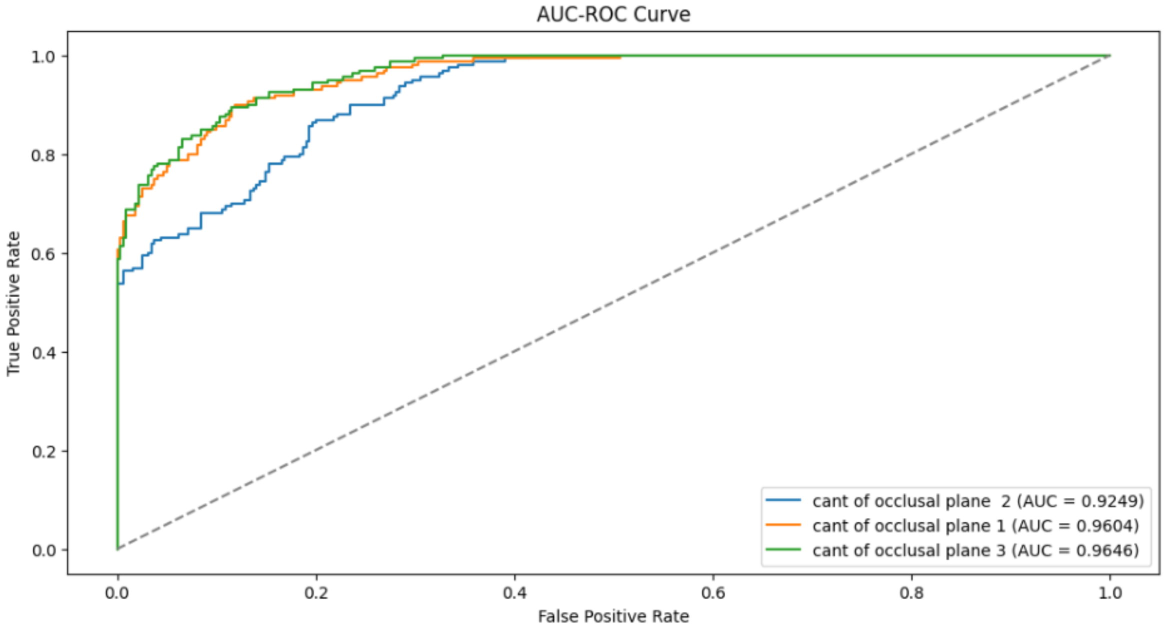

Figure S1. 14 Precision–recall curve for Cant of Occlusal Plane classified by EfficientNet B0

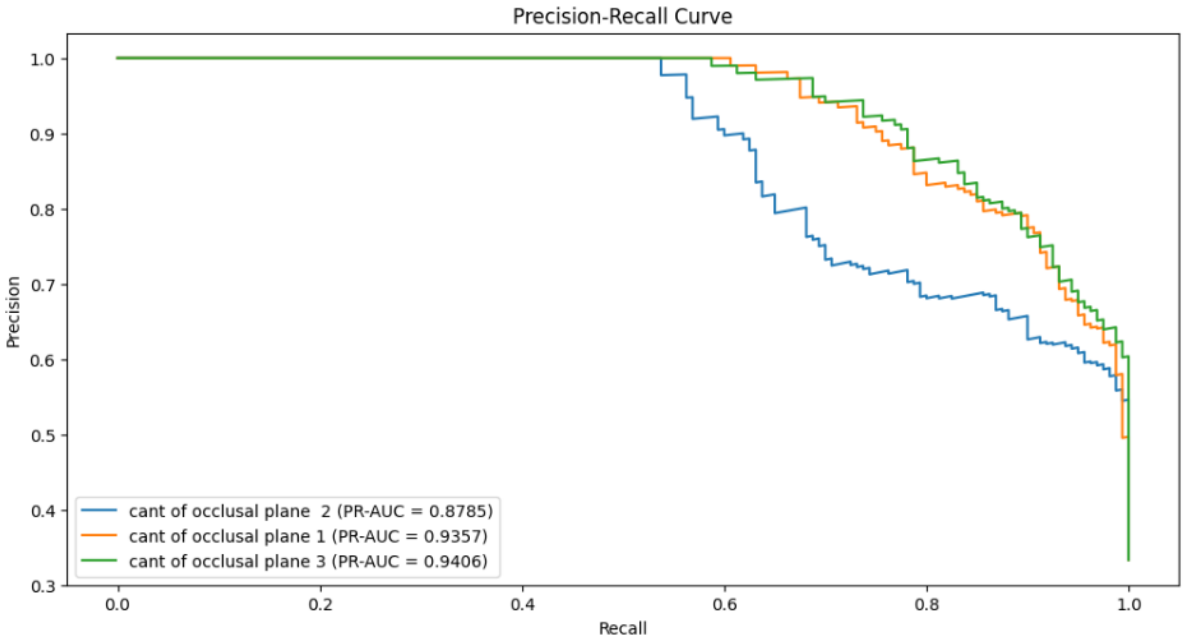

Table S1. 3 Classification Report for Cant of Occlusal Plane by EfficientNet B0

Mean Absolute Error (MAE): 0.2979
Cohen's Kappa: 0.6875
Classification Report:

	precision	recall	f1-score	support
cant of occlusal plane 2	0.7261	0.7125	0.7192	160
cant of occlusal plane 1	0.8323	0.8063	0.8190	160
cant of occlusal plane 3	0.8155	0.8562	0.8354	160
accuracy			0.7917	480
macro avg	0.7913	0.7917	0.7912	480
weighted avg	0.7913	0.7917	0.7912	480

Figure S1. 15 The original and Grad-CAM Images for Cant of Occlusal Plane Generated by EfficientNet B0

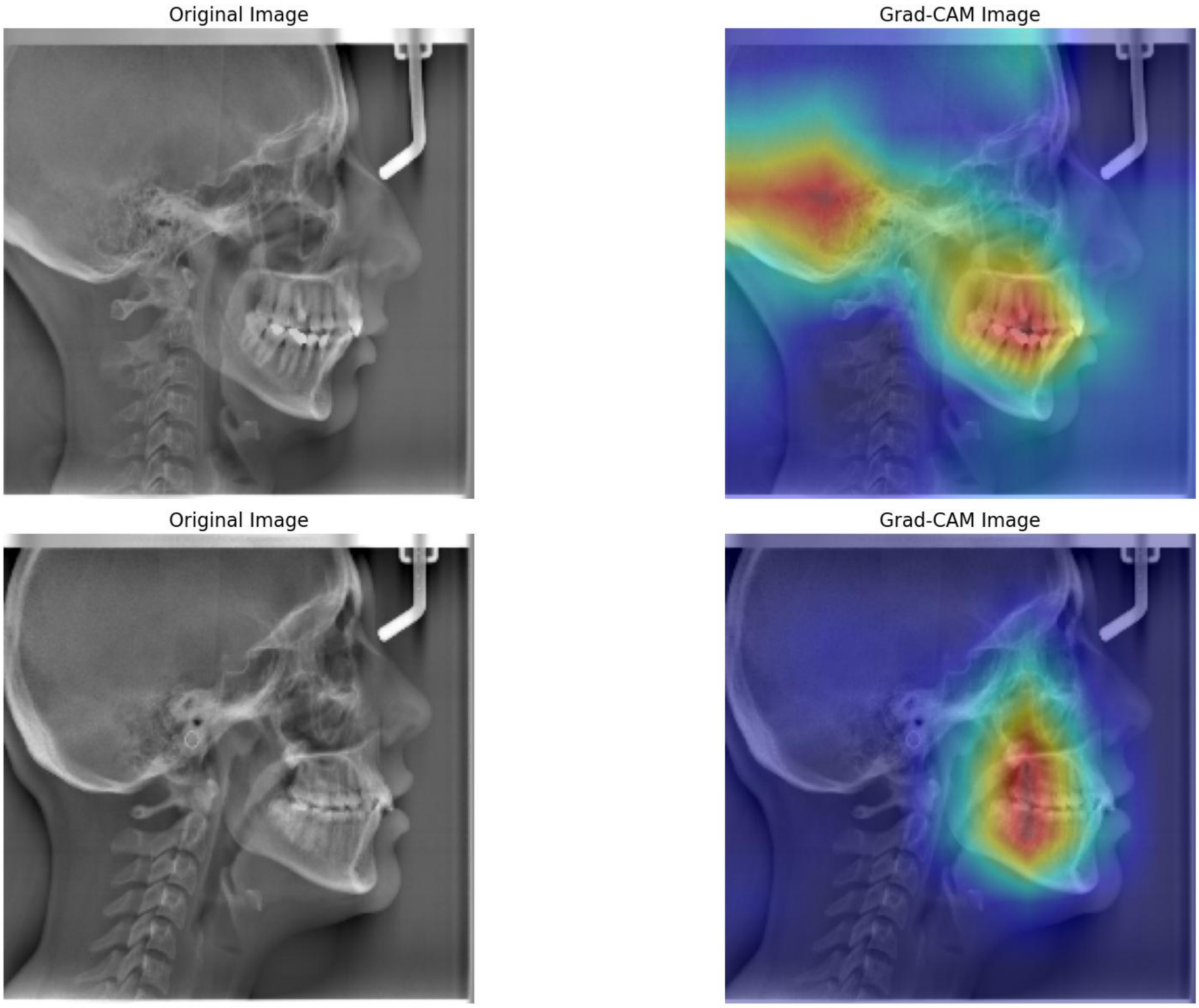

1.4 Classification of Cant of Occlusal Plane by EfficientNet V2

Figure S1. 16 Training and Testing Loss and Training and Testing Accuracy Graphs for EfficientNet V2

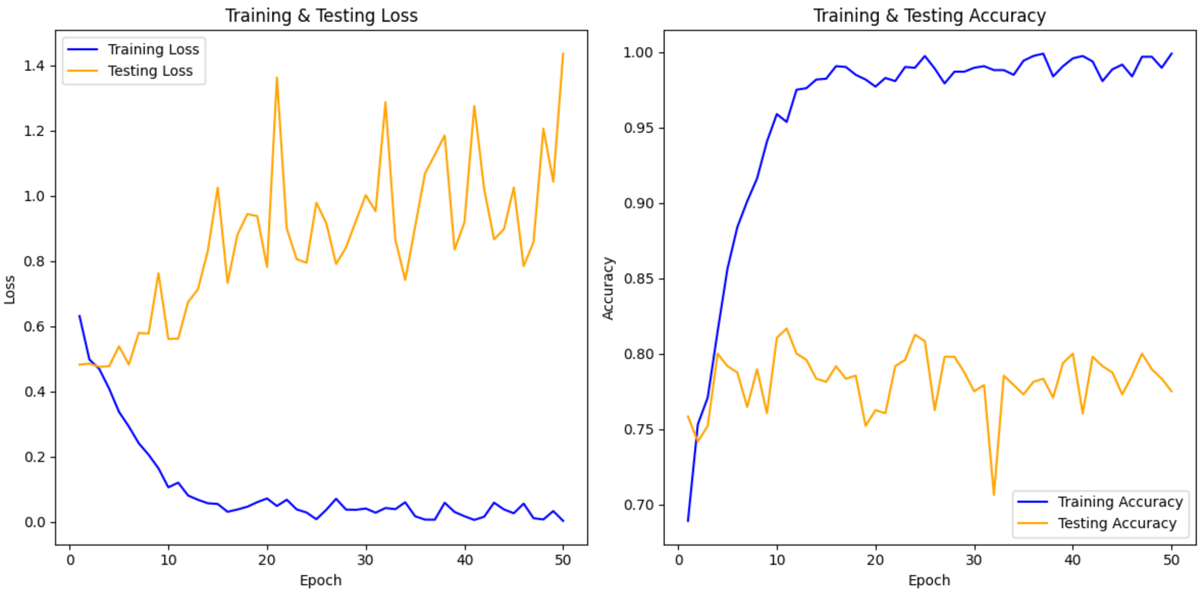

Figure S1. 17 Confusion Matrix for Actual and Predicted Cant of Occlusal Plane values classified by EfficientNet V2

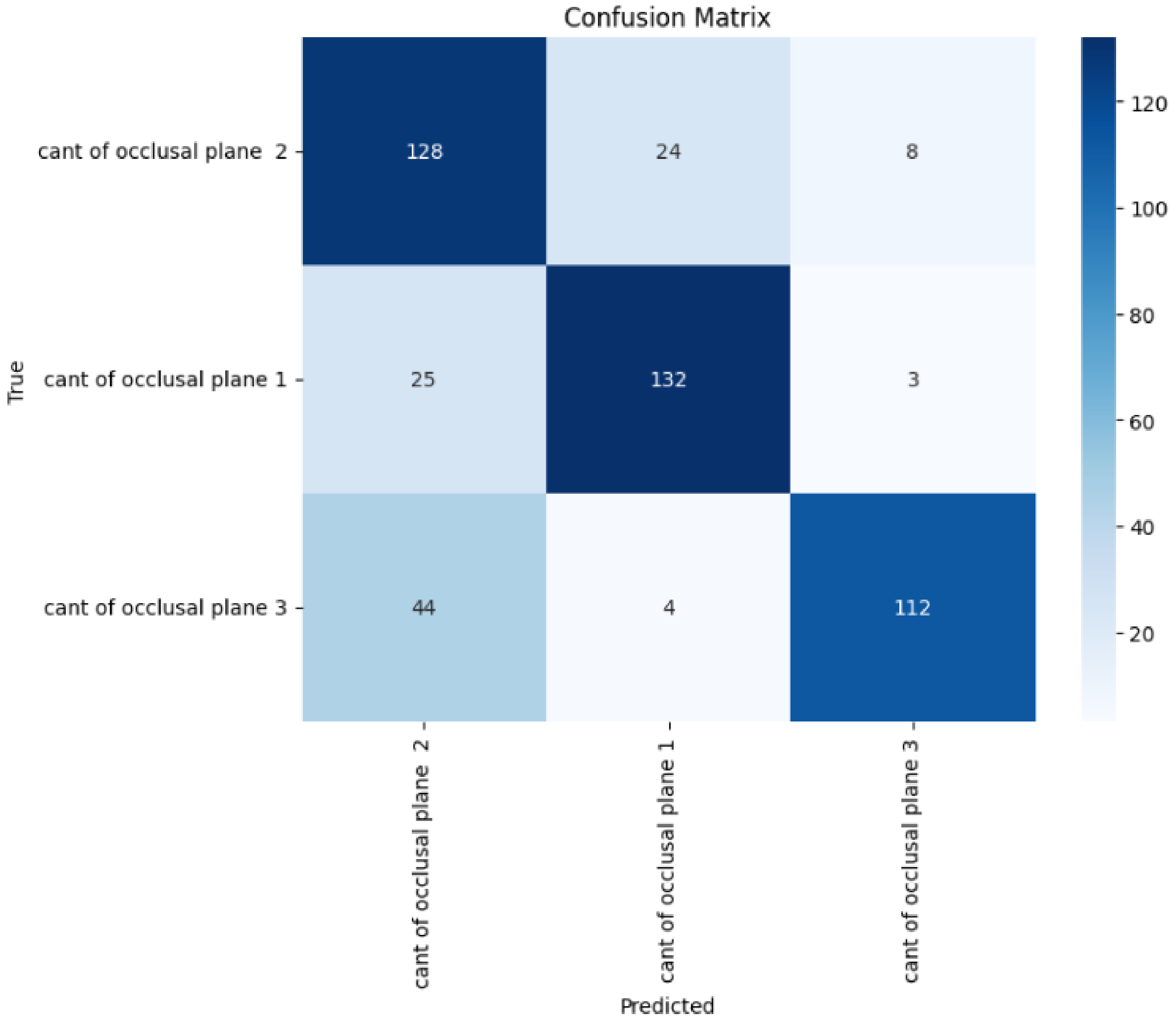

Figure S1. 18 AUC-ROC curve for Cant of Occlusal Plane classified by EfficientNet V2

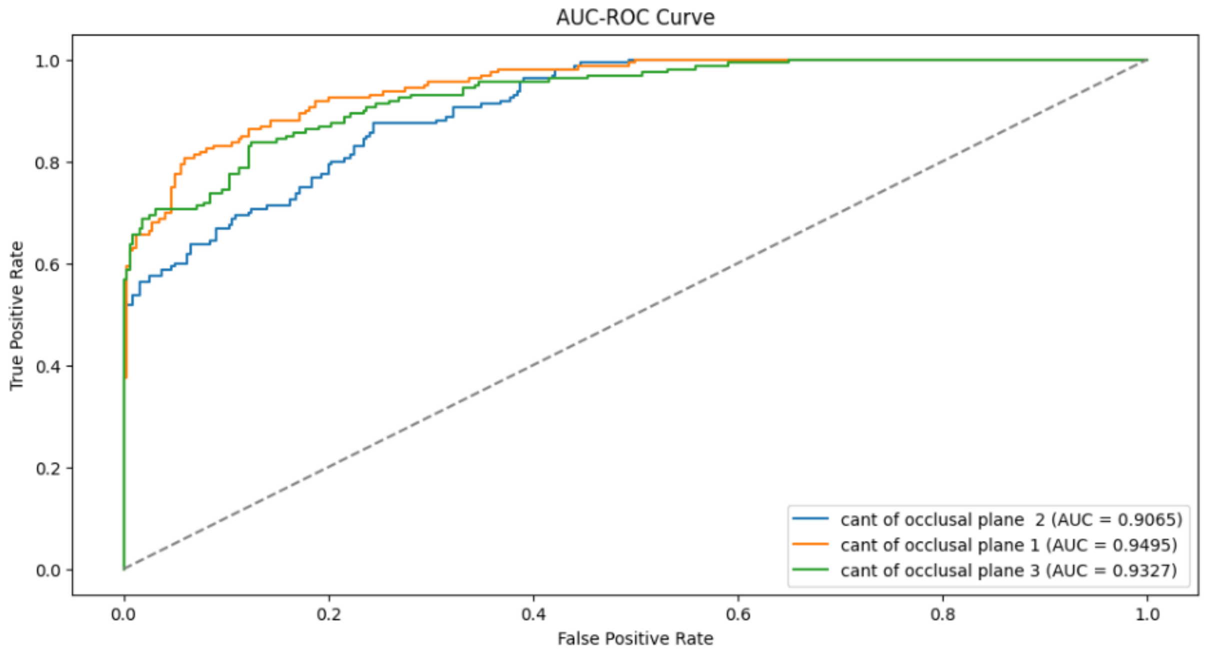

Figure S1. 19 Precision–recall curve for Cant of Occlusal Plane classified by EfficientNet V2

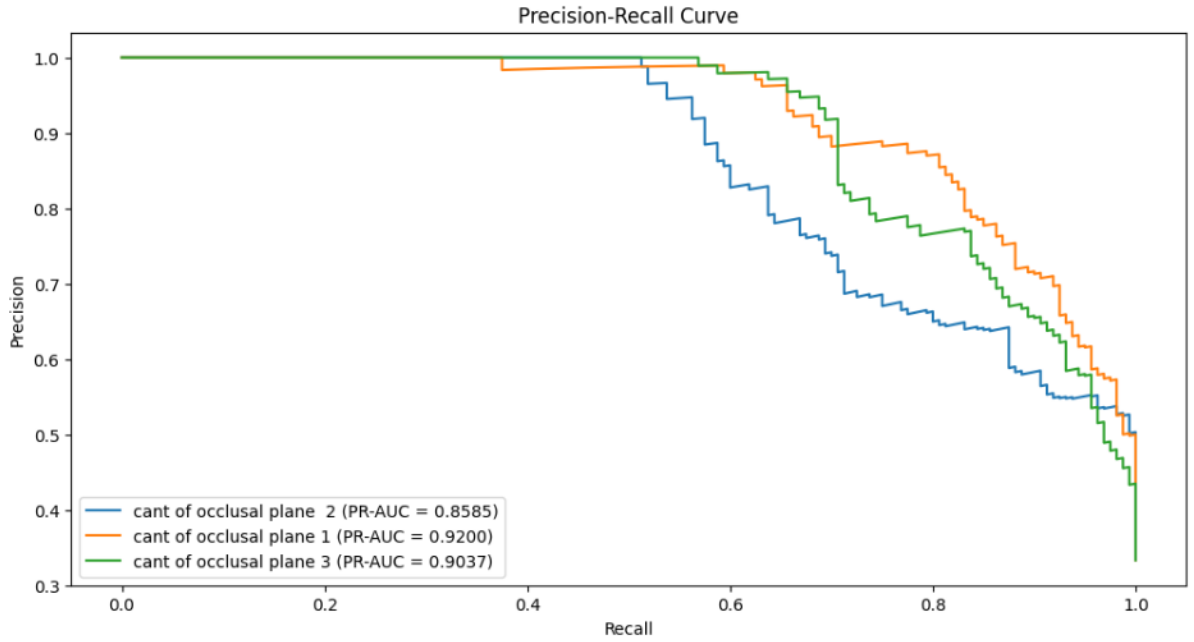

Table S1. 4 Classification Report for Cant of Occlusal Plane by EfficientNet V2

Mean Absolute Error (MAE): 0.3333
Cohen's Kappa: 0.6625
Classification Report:

	precision	recall	f1-score	support
cant of occlusal plane 2	0.6497	0.8000	0.7171	160
cant of occlusal plane 1	0.8250	0.8250	0.8250	160
cant of occlusal plane 3	0.9106	0.7000	0.7915	160
accuracy			0.7750	480
macro avg	0.7951	0.7750	0.7779	480
weighted avg	0.7951	0.7750	0.7779	480

Figure S1. 20 The original and Grad-CAM Images for Cant of Occlusal Plane Generated by EfficientNet V2

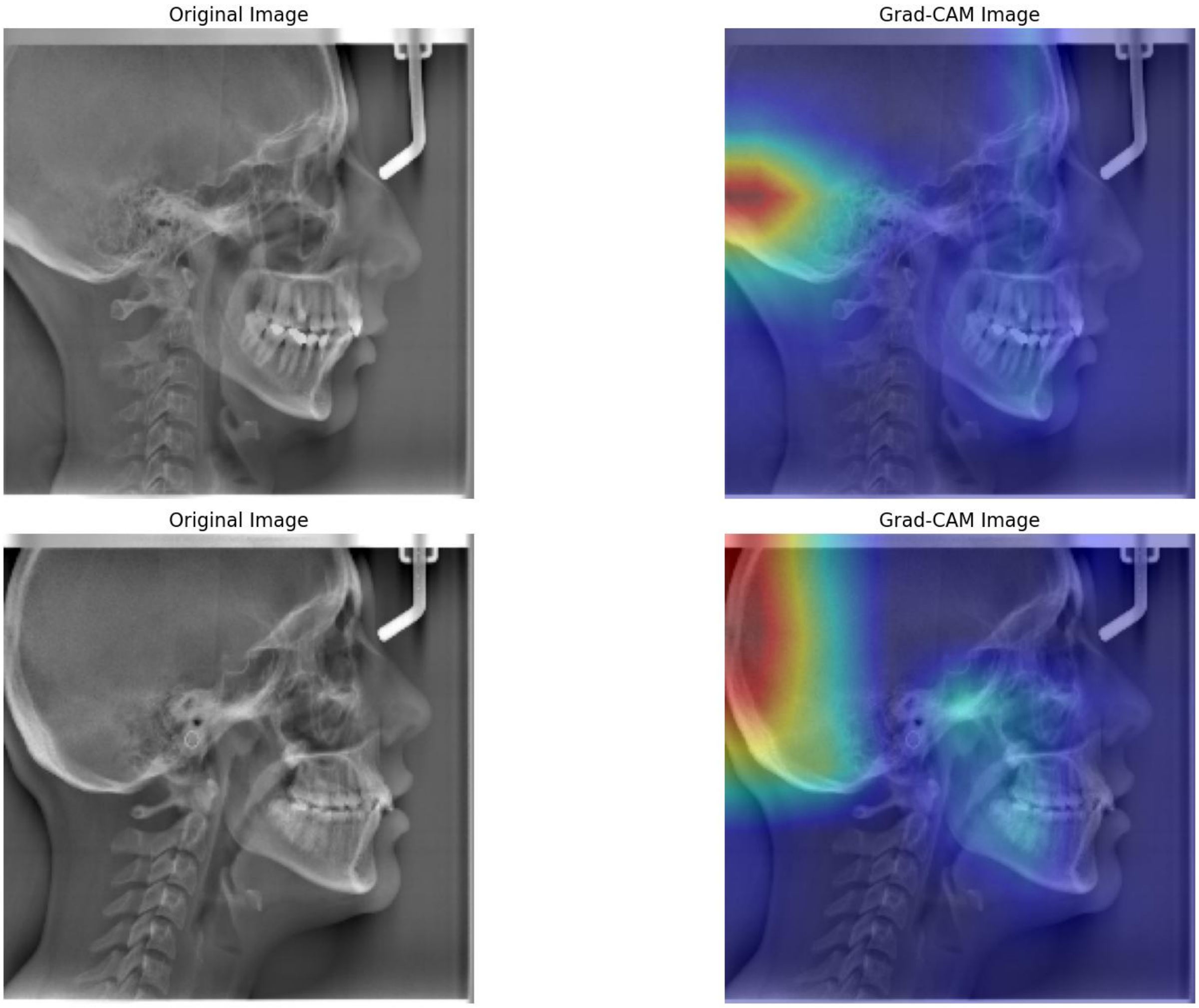

1.5 Classification of Cant of Occlusal Plane by Hybrid Algorithm

Figure S1. 21 Training and Testing Loss and Training and Testing Accuracy Graphs for Hybrid Algorithm

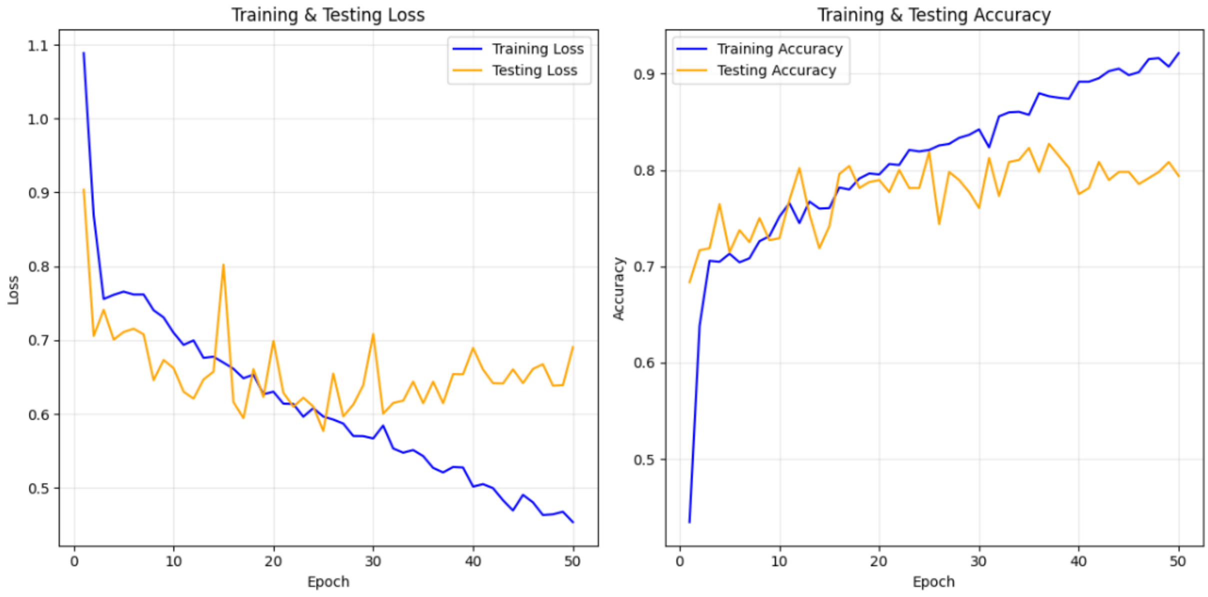

Figure S1. 22 Confusion Matrix for Actual and Predicted Cant of Occlusal Plane values classified by Hybrid Algorithm

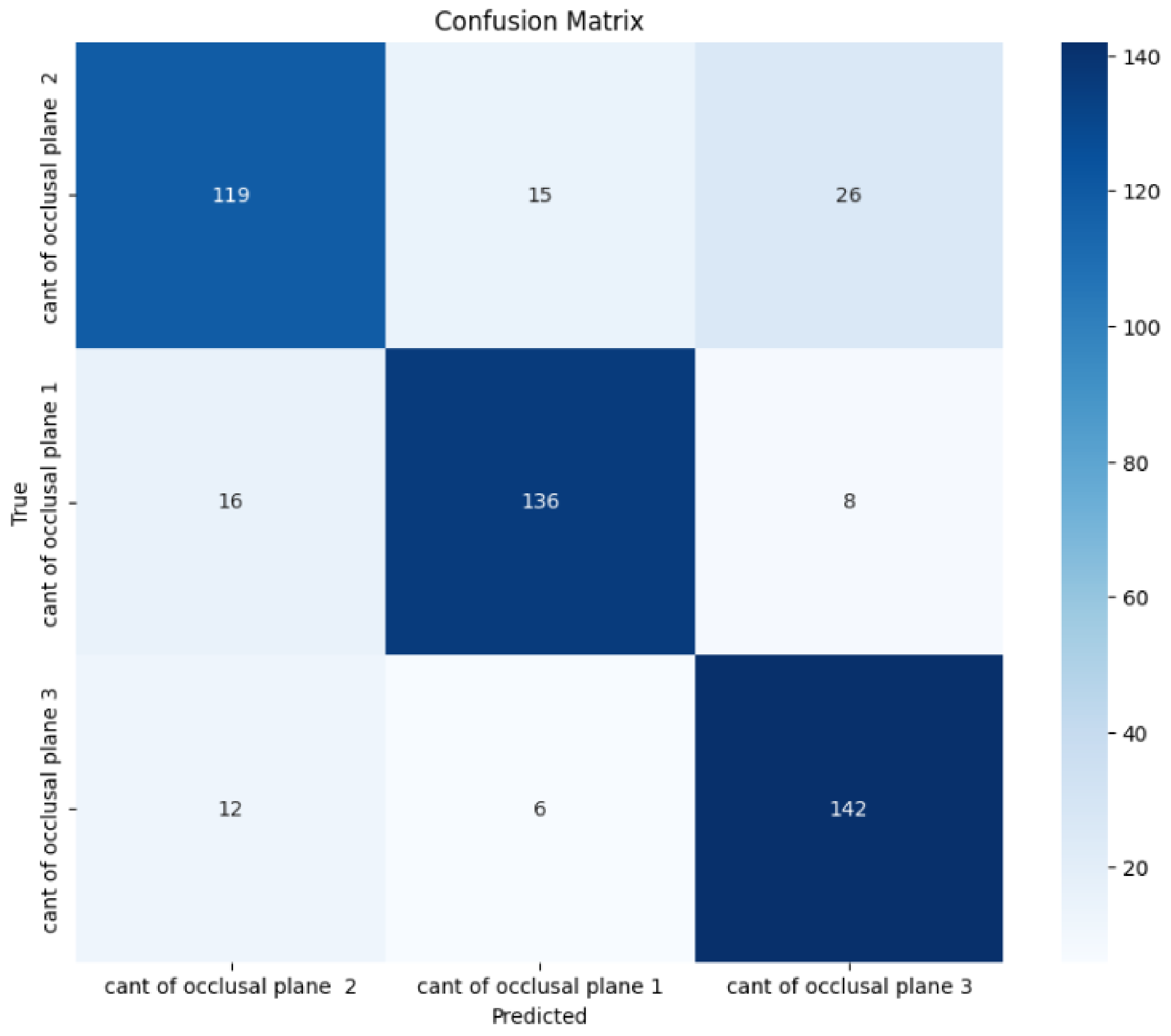

Figure S1. 23 AUC-ROC curve for Cant of Occlusal Plane classified by Hybrid Algorithm

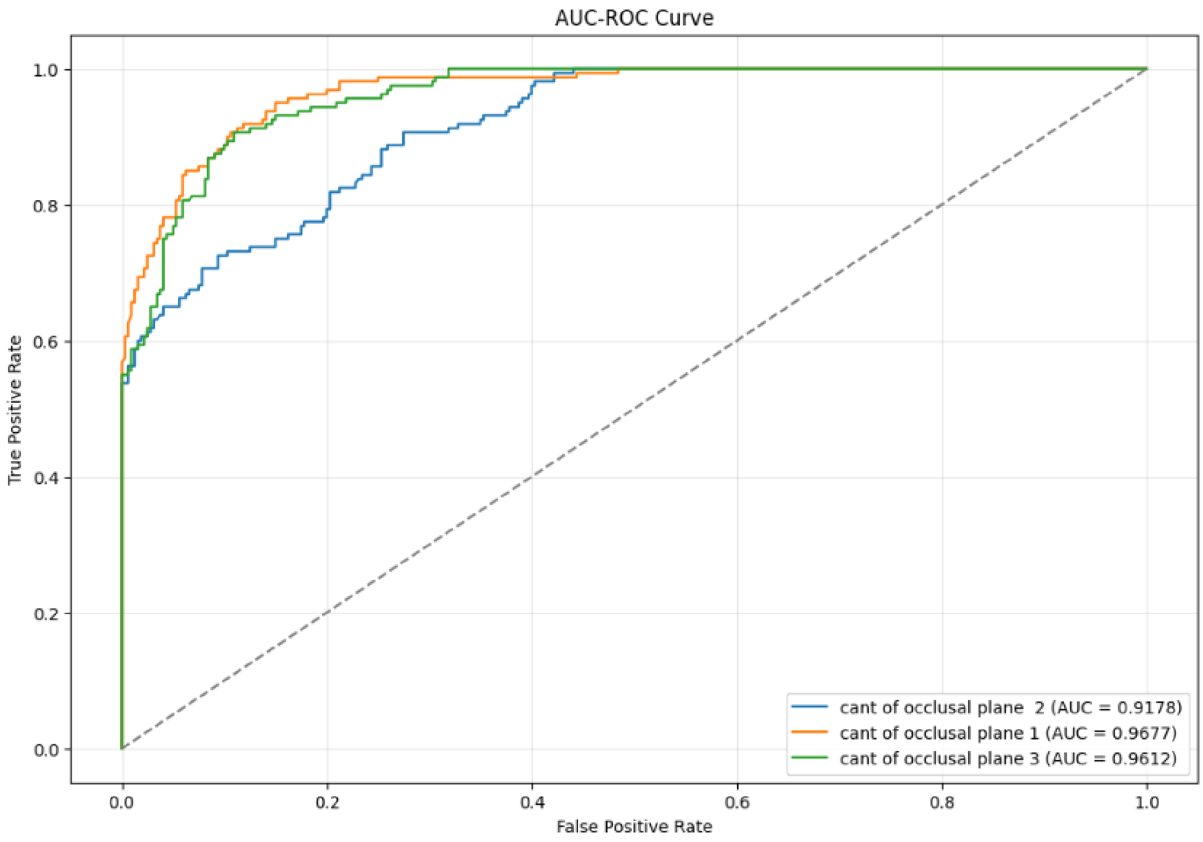

Figure S1. 24 Precision–recall curve for Cant of Occlusal Plane classified by Hybrid Algorithm

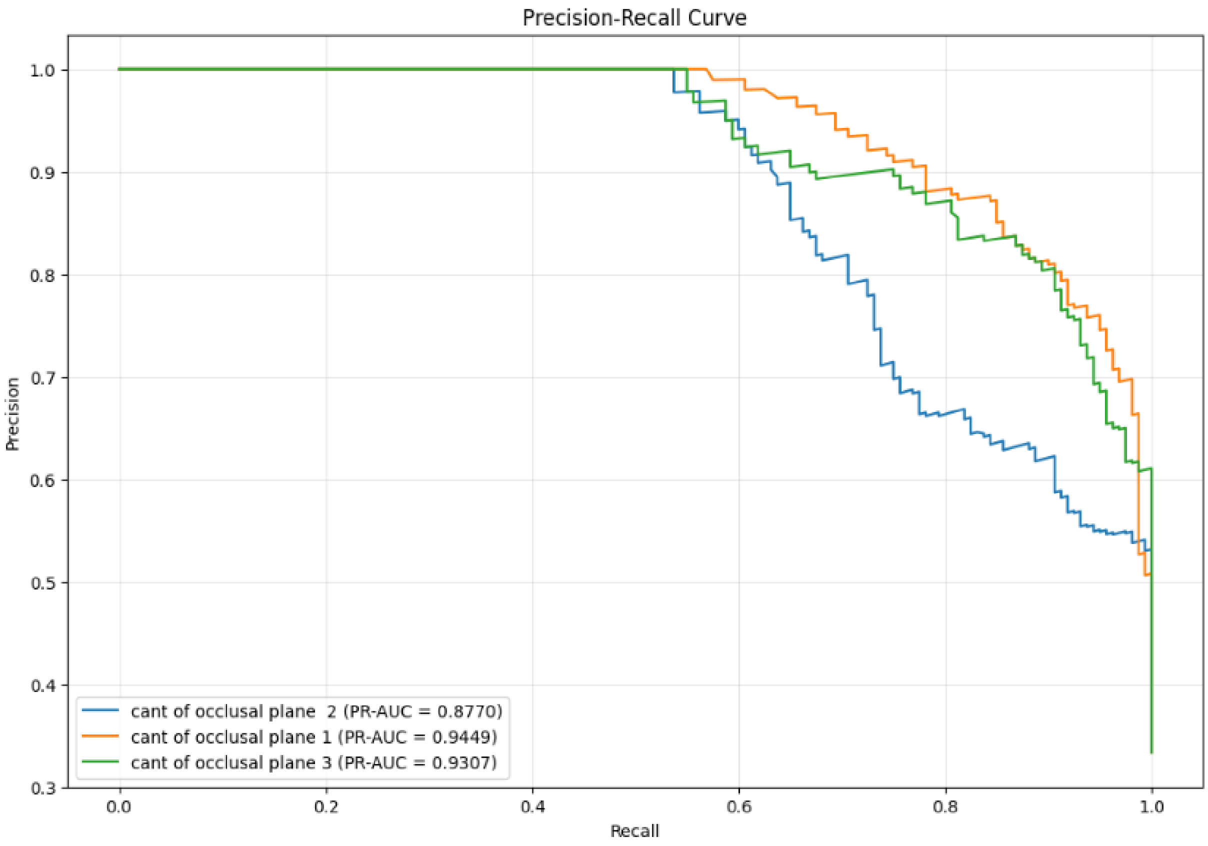

Table S1. 5 Classification Report for Cant of Occlusal Plane by Hybrid Algorithm

Mean Absolute Error (MAE): 0.2521
Cohen's Kappa: 0.7406
Classification Report:

	precision	recall	f1-score	support
cant of occlusal plane 2	0.8095	0.7438	0.7752	160
cant of occlusal plane 1	0.8662	0.8500	0.8580	160
cant of occlusal plane 3	0.8068	0.8875	0.8452	160
accuracy			0.8271	480
macro avg	0.8275	0.8271	0.8262	480
weighted avg	0.8275	0.8271	0.8262	480

Figure S1. 25 The original and Grad-CAM Images for Cant of Occlusal Plane Generated by Hybrid Algorithm

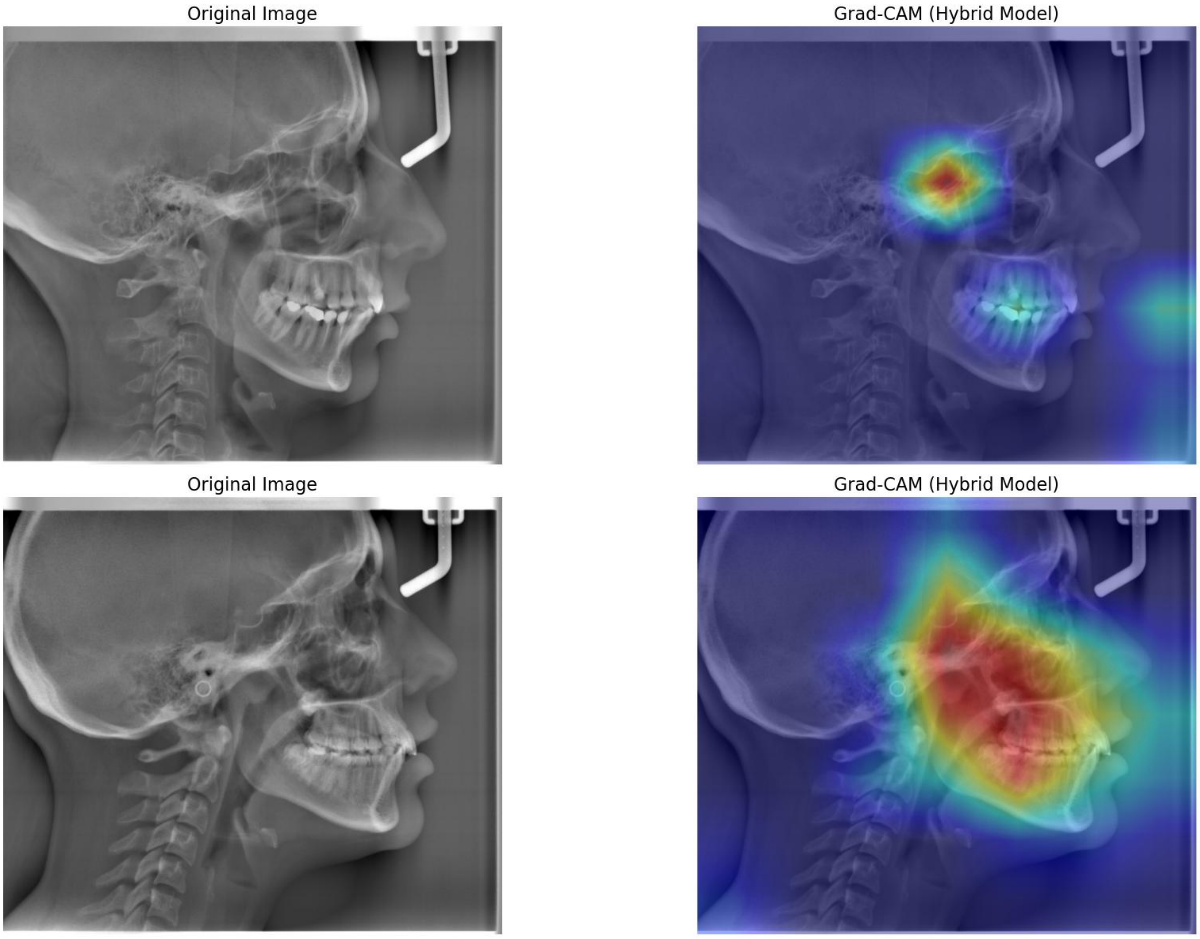

1.6 Classification of Cant of Occlusal Plane by MobileNetV2

Figure S1. 26 Training and Testing Loss and Training and Testing Accuracy Graphs for MobileNetV2

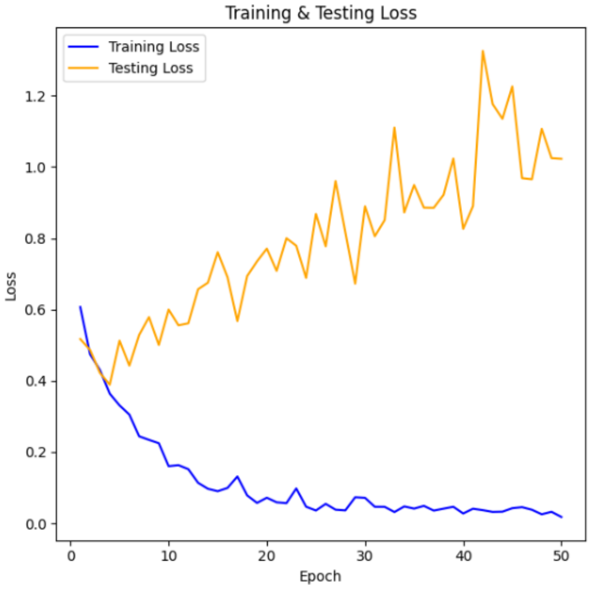

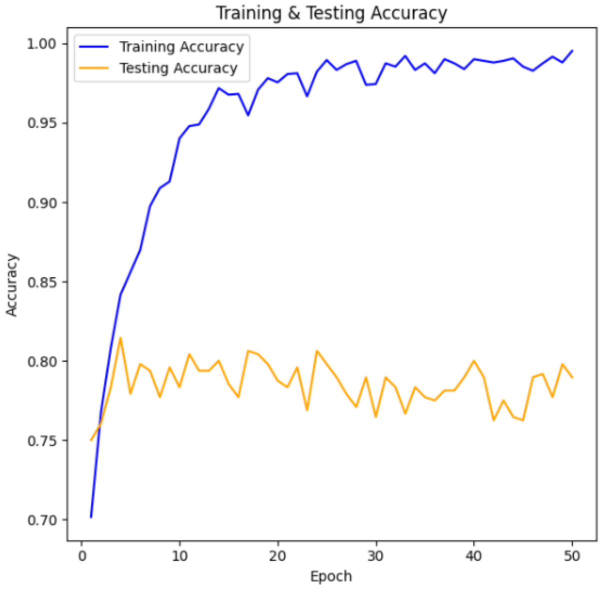

Figure S1. 27 Confusion Matrix for Actual and Predicted Cant of Occlusal Plane values classified by MobileNetV2

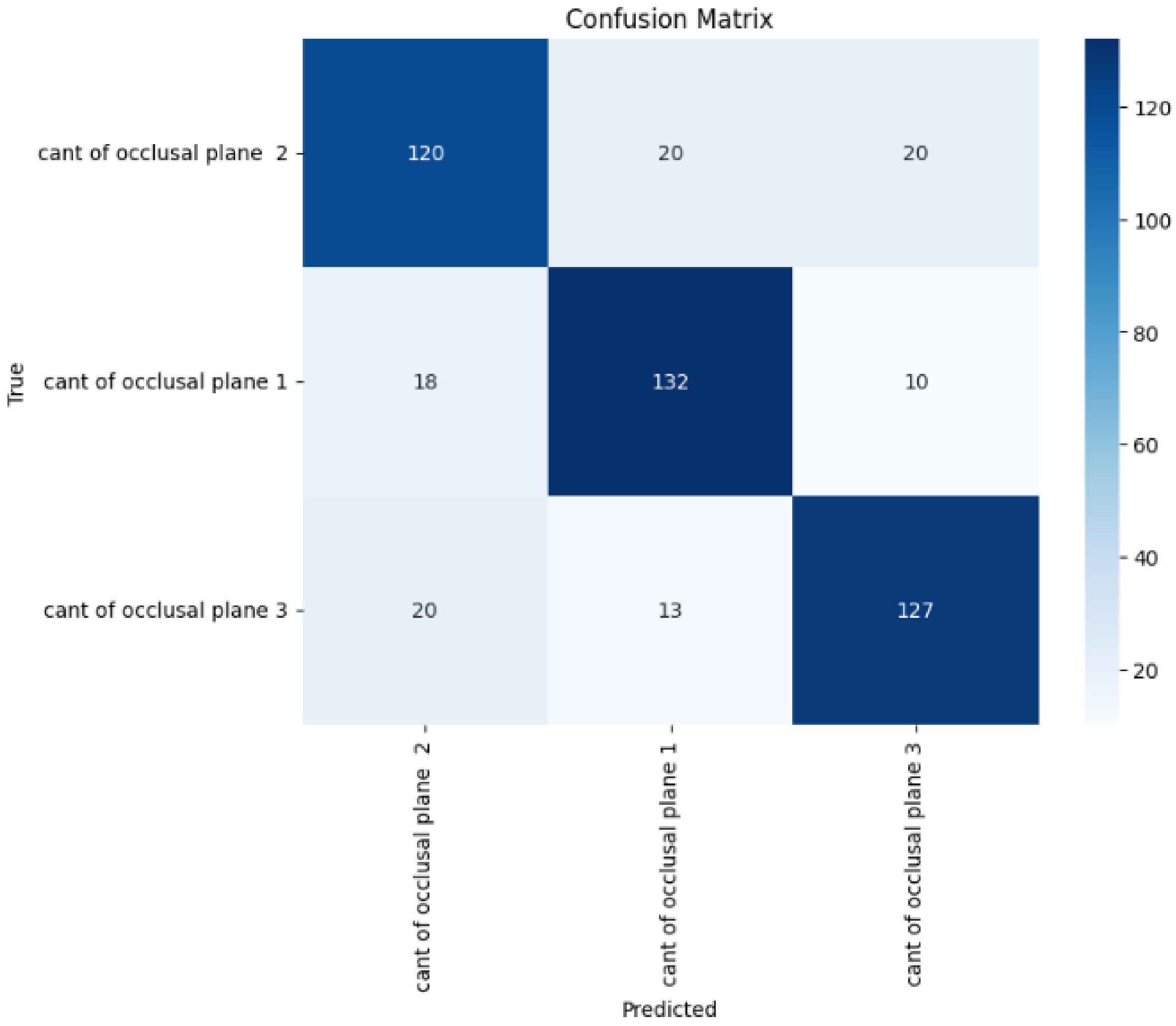

Figure S1. 28 AUC-ROC curve for Cant of Occlusal Plane classified by MobileNetV2

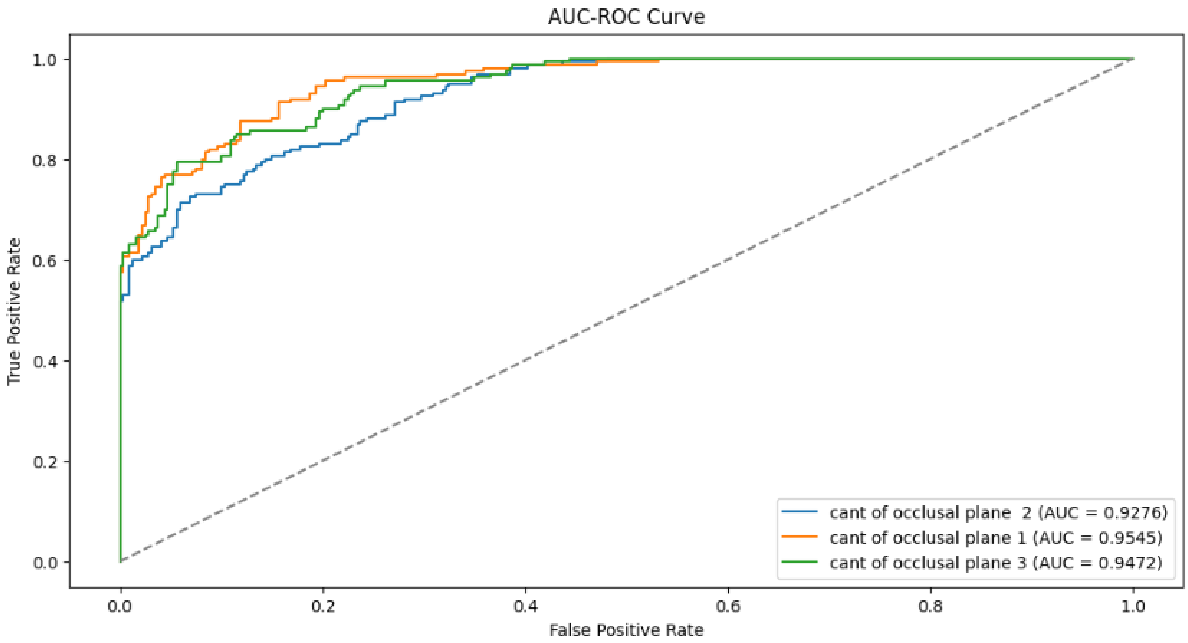

Figure S1. 29 Precision–recall curve for Cant of Occlusal Plane classified by MobileNetV2

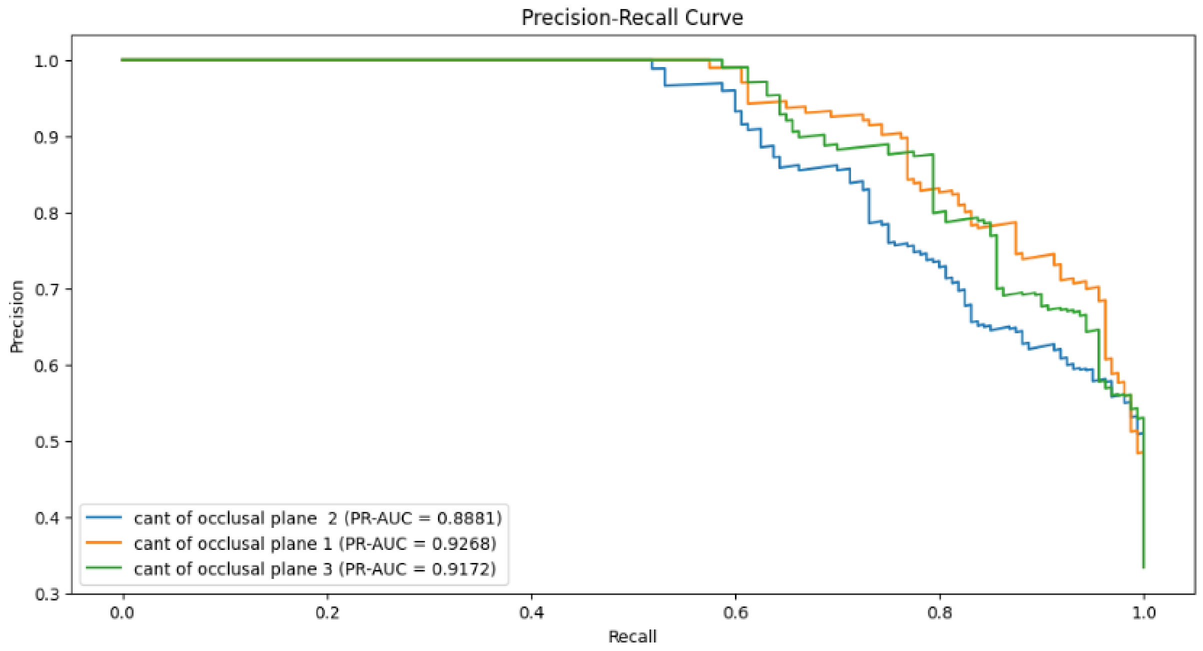

Table S1. 6 Classification Report for Cant of Occlusal Plane by MobileNetV2

Mean Absolute Error (MAE): 0.2938
Cohen's Kappa: 0.6844
Classification Report:

	precision	recall	f1-score	support
cant of occlusal plane 2	0.7595	0.7500	0.7547	160
cant of occlusal plane 1	0.8000	0.8250	0.8123	160
cant of occlusal plane 3	0.8089	0.7937	0.8013	160
accuracy			0.7896	480
macro avg	0.7895	0.7896	0.7894	480
weighted avg	0.7895	0.7896	0.7894	480

Figure S1. 30 The original and Grad-CAM Images for Cant of Occlusal Plane Generated by MobileNetV2

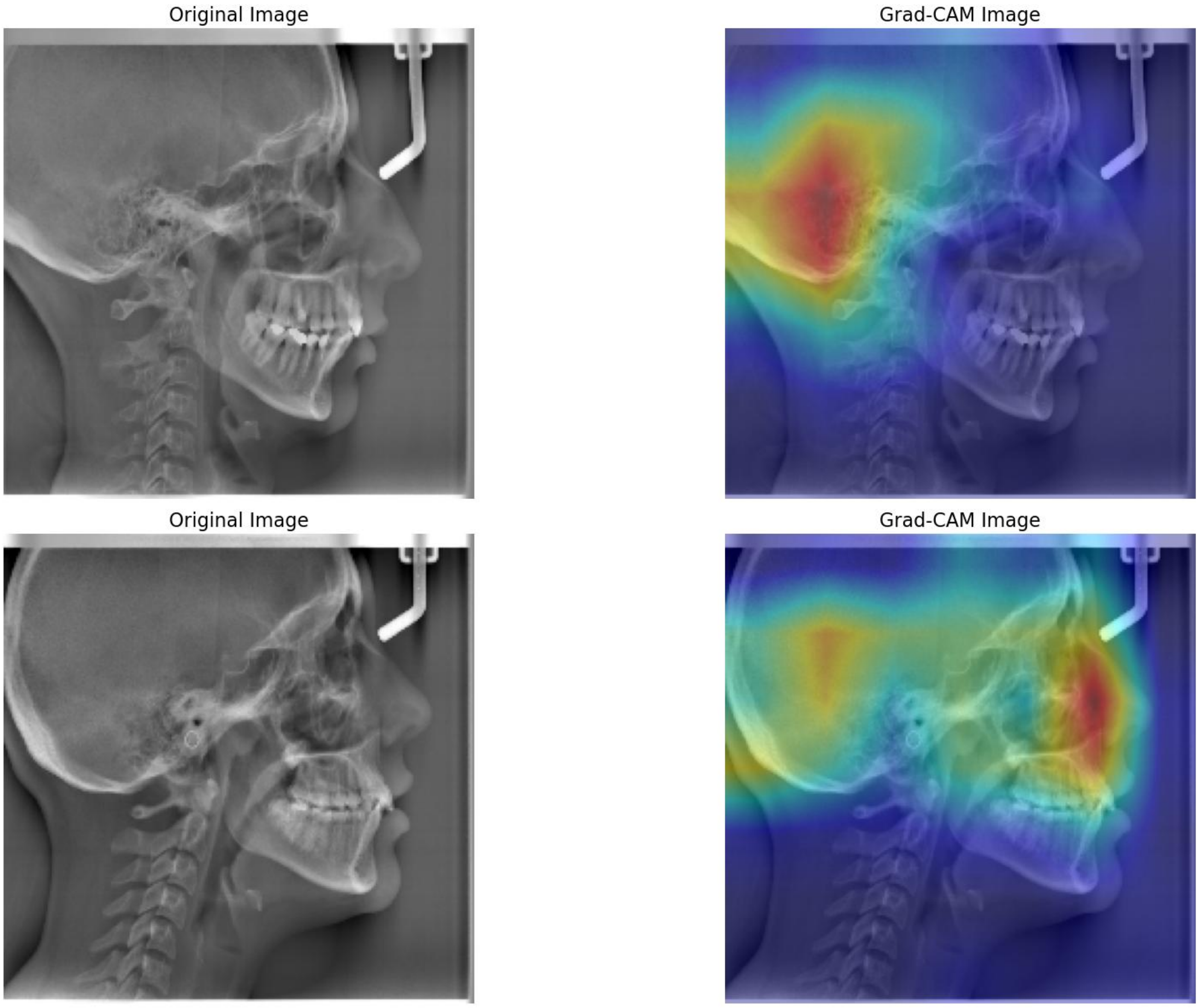

1.7 Classification of Cant of Occlusal Plane by ResNet101

Figure S1. 31 Training and Testing Loss and Training and Testing Accuracy Graphs for ResNet101

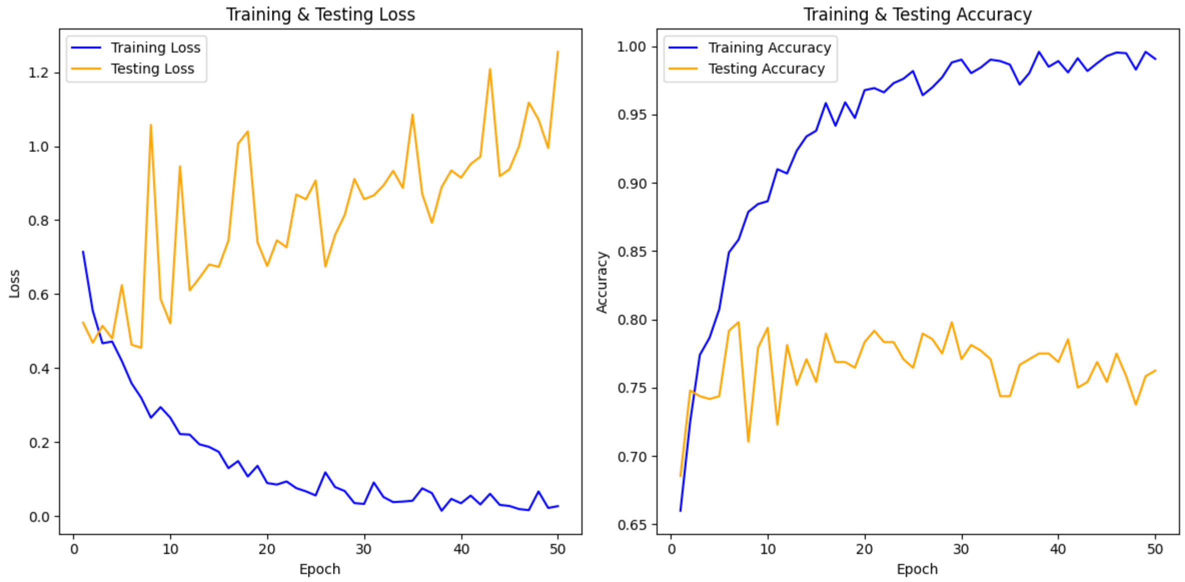

Figure S1. 32 Confusion Matrix for Actual and Predicted Cant of Occlusal Plane values classified by ResNet101

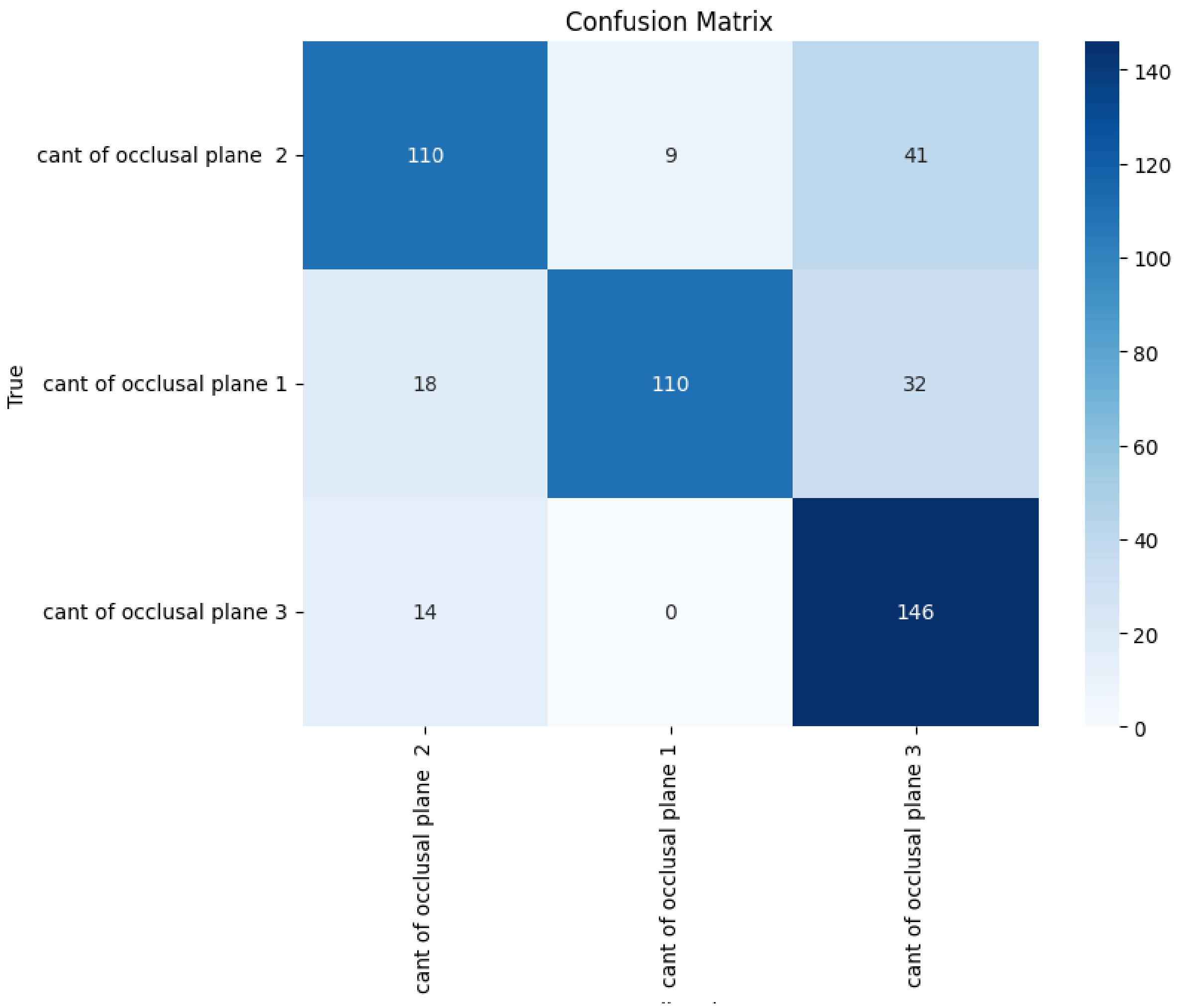

Figure S1. 33 AUC-ROC curve for Cant of Occlusal Plane classified by ResNet101

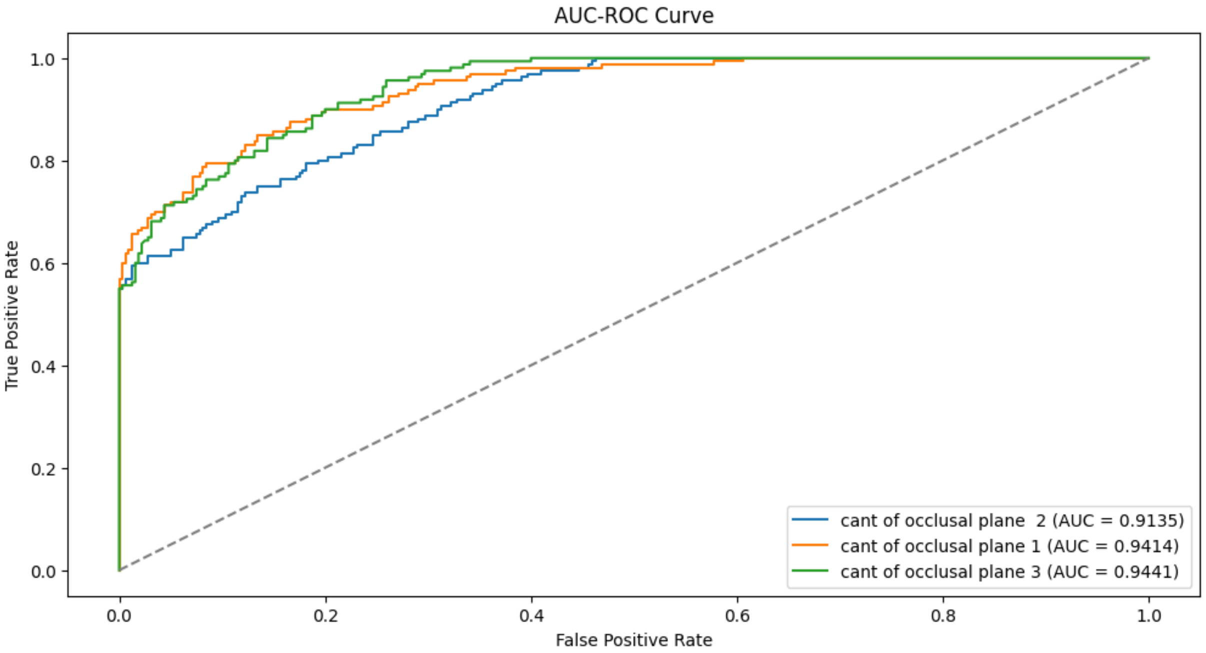

Figure S1. 34 Precision–recall curve for Cant of Occlusal Plane classified by ResNet101

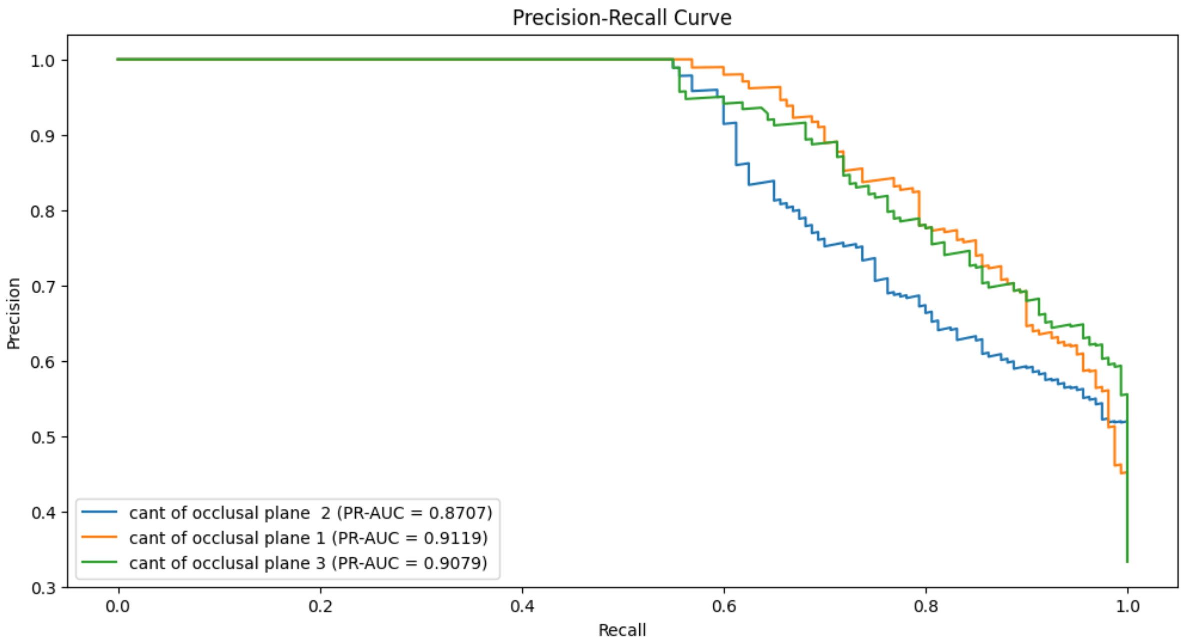

Table S1. 7 Classification Report for Cant of Occlusal Plane by ResNet101

Mean Absolute Error (MAE): 0.3521
Cohen's Kappa: 0.6438
Classification Report:

	precision	recall	f1-score	support
cant of occlusal plane 2	0.7746	0.6875	0.7285	160
cant of occlusal plane 1	0.9244	0.6875	0.7885	160
cant of occlusal plane 3	0.6667	0.9125	0.7704	160
accuracy			0.7625	480
macro avg	0.7886	0.7625	0.7625	480
weighted avg	0.7886	0.7625	0.7625	480

Figure S1. 35 The original and Grad-CAM Images for Cant of Occlusal Plane Generated by ResNet101

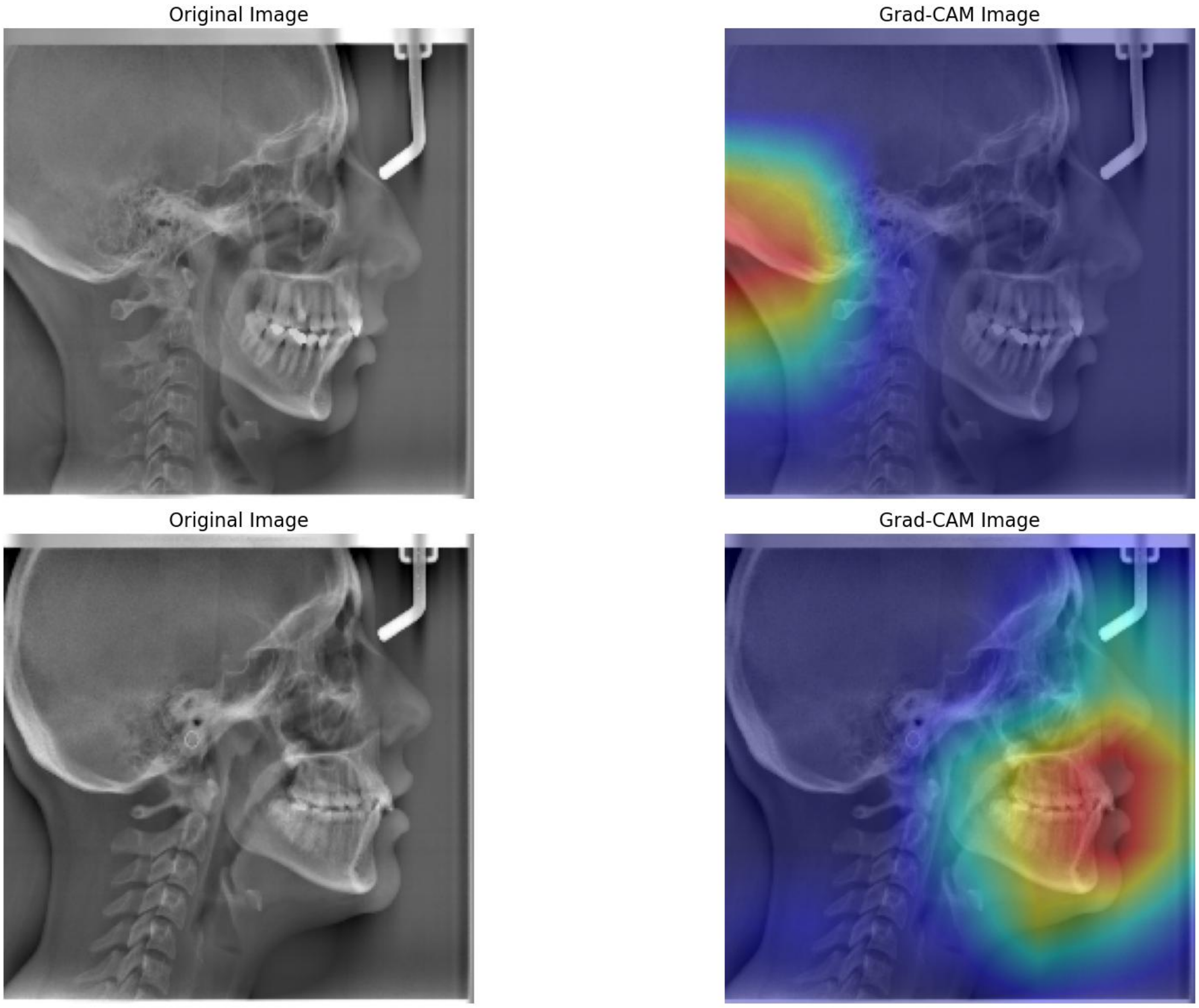

Figure S1. 36 Comparisons of Models According to Accuracy , Mean Absolute Error and Cohen's Kappa for Cant of Occlusal Plane

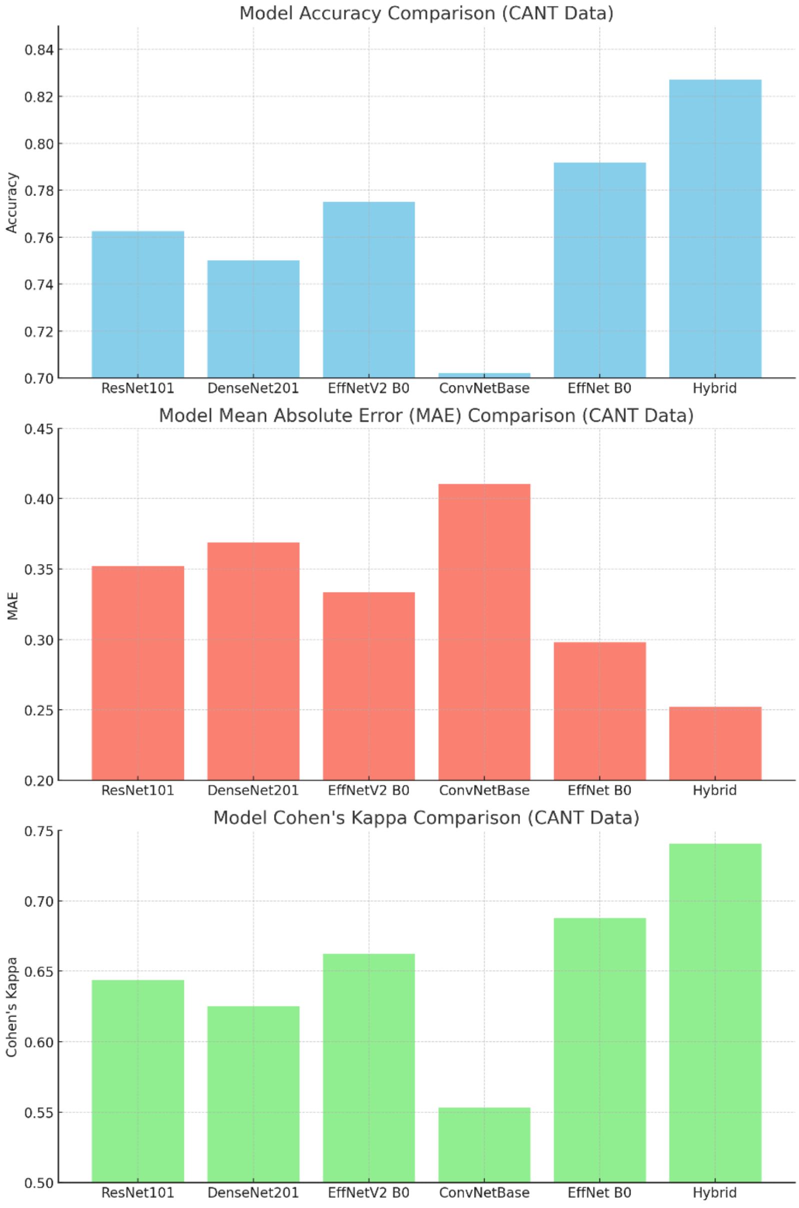

Supplement: Supplementary file 1 [file diagnostics-15-02240-s001.zip › 1 Cant of Occlusal Plane_Rev.pdf]
