# Supplementary material for: Classification Performance of Deep Learning Models for the Assessment of Vertical Dimension on Lateral Cephalometric Radiographs
Source: Diagnostics (Basel). 2025 Sep 3;15(17):2240. doi: 10.3390/diagnostics15172240 (PMC12428445; doi:10.3390/diagnostics15172240)

2.1 Classification of FMA by ConvNet

Figure S2. 1 Training and Testing Loss and Training and Testing Accuracy Graphs for ConvNet

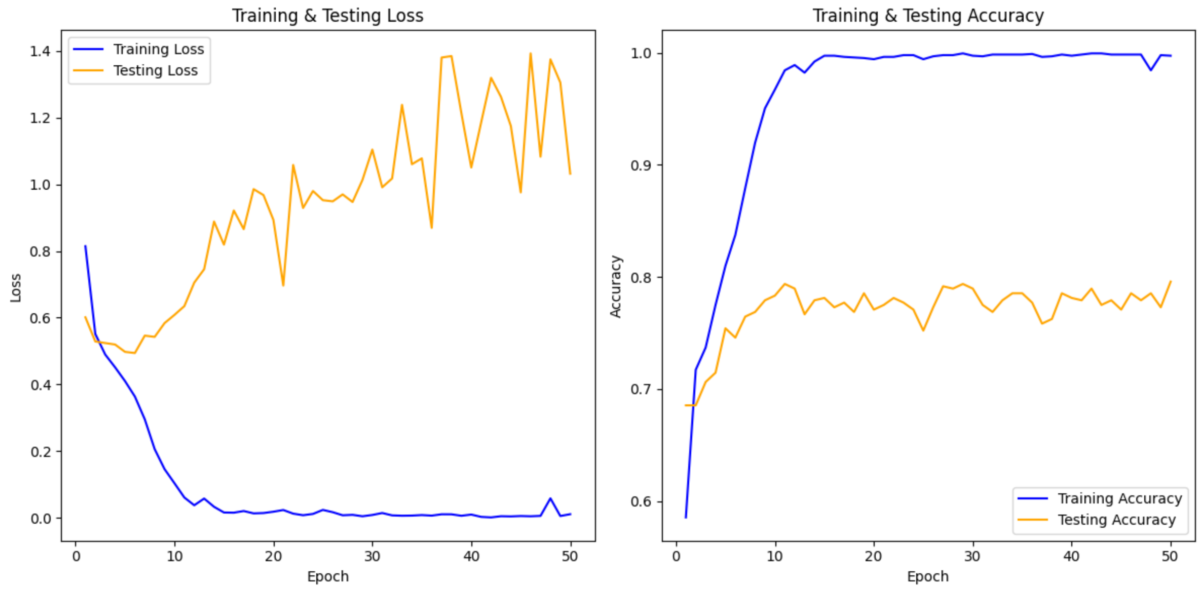

Figure S2. 2 Confusion Matrix for Actual and Predicted FMA values classified by ConvNet

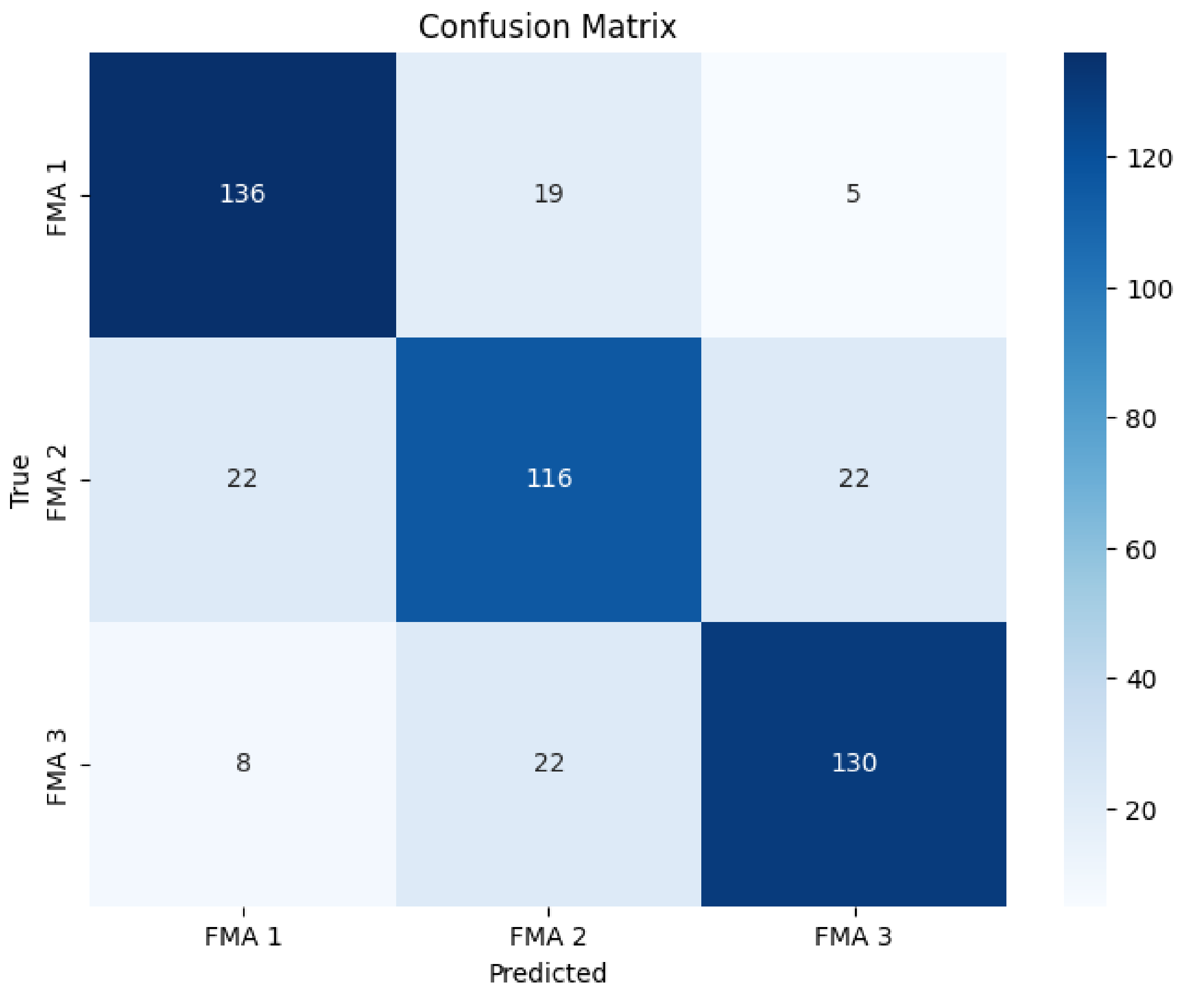

Figure S2. 3 AUC-ROC curve for FMA classified by ConvNet

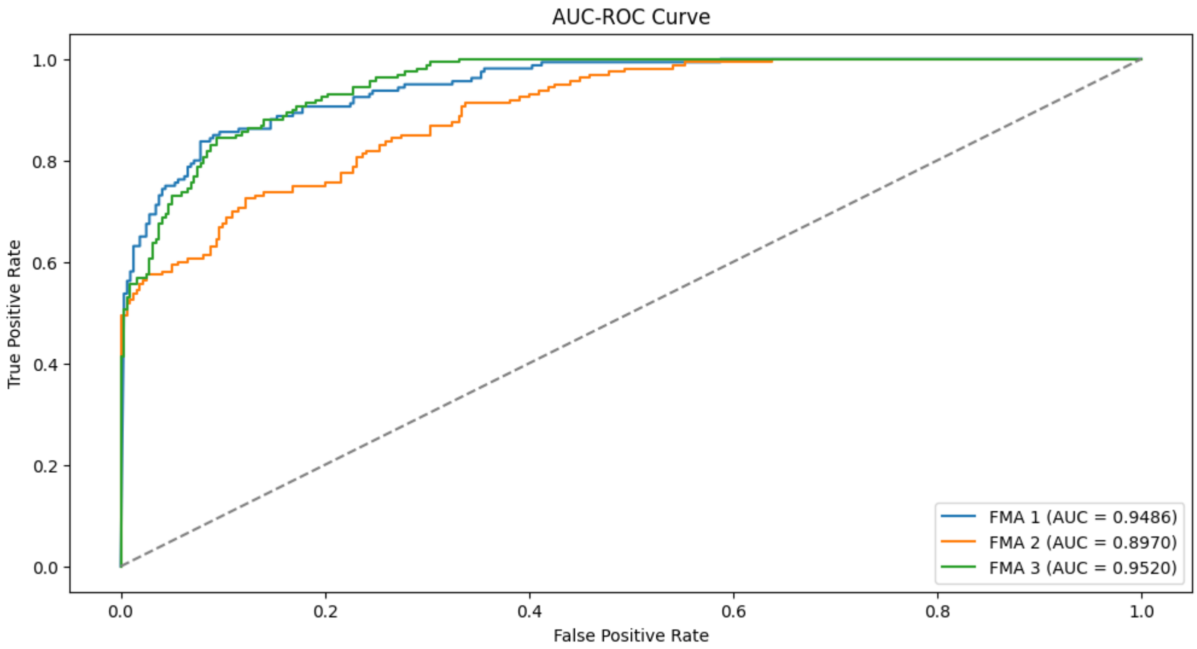

Figure S2. 4 Precision–recall curve for FMA classified by ConvNet

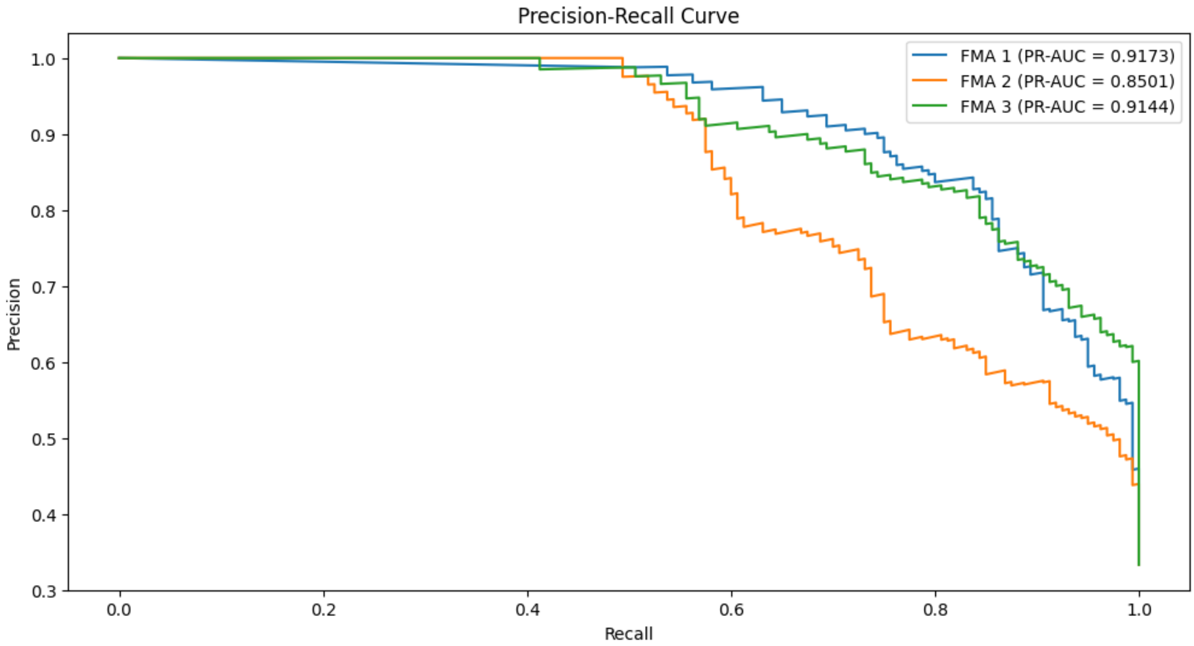

Table S2. 1 Classification Report for FMA by ConvNet

Mean Absolute Error (MAE): 0.2313
Cohen's Kappa: 0.6937
Classification Report:

	precision	recall	f1-score	support
FMA 1	0.8193	0.8500	0.8344	160
FMA 2	0.7389	0.7250	0.7319	160
FMA 3	0.8280	0.8125	0.8202	160
accuracy			0.7958	480
macro avg	0.7954	0.7958	0.7955	480
weighted avg	0.7954	0.7958	0.7955	480

Figure S2. 5 The original and Grad-CAM Images for FMA Generated by ConvNet

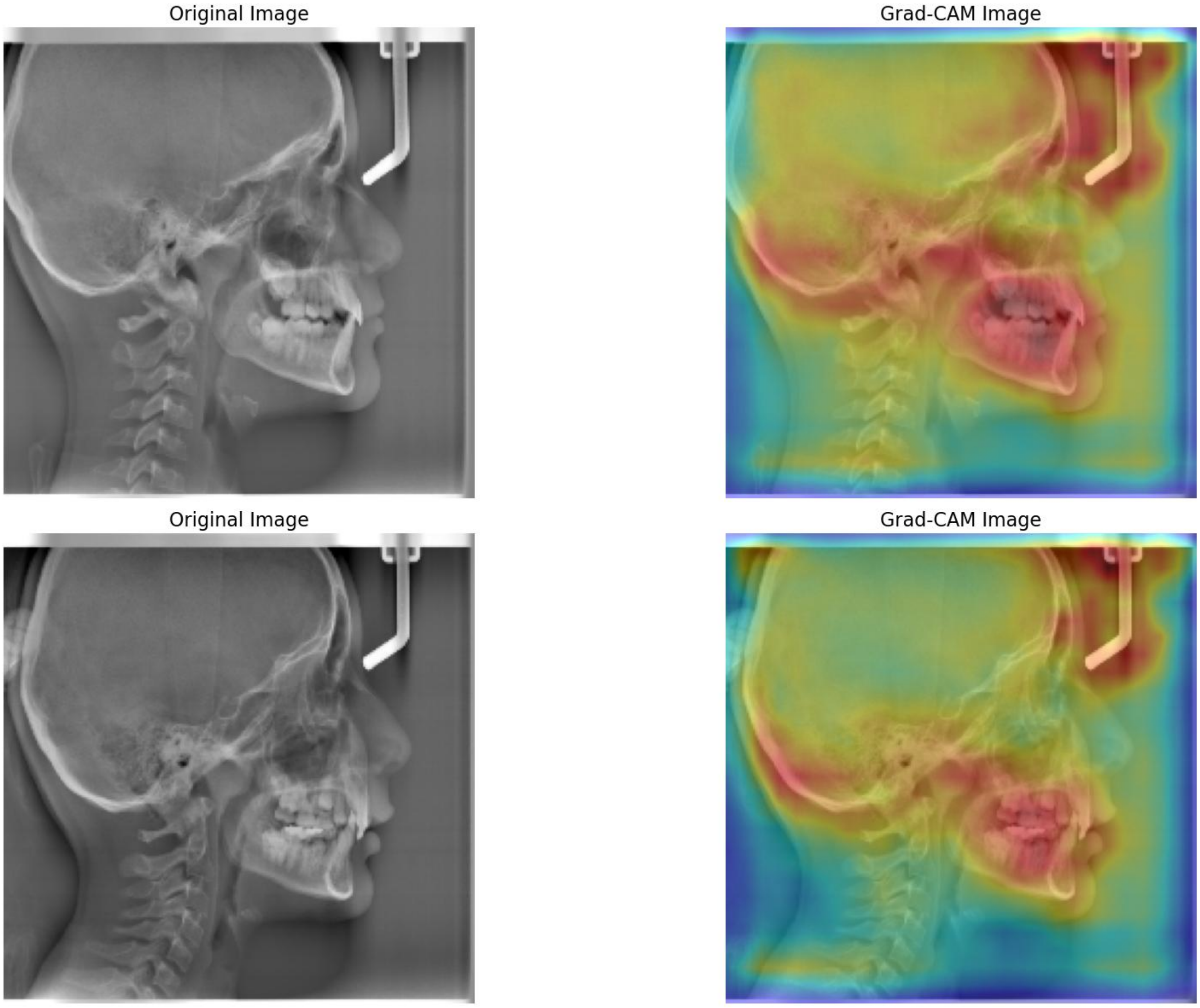

2.2 Classification of FMA by DenseNet201

Figure S2. 6 Training and Testing Loss and Training and Testing Accuracy Graphs for DenseNet201

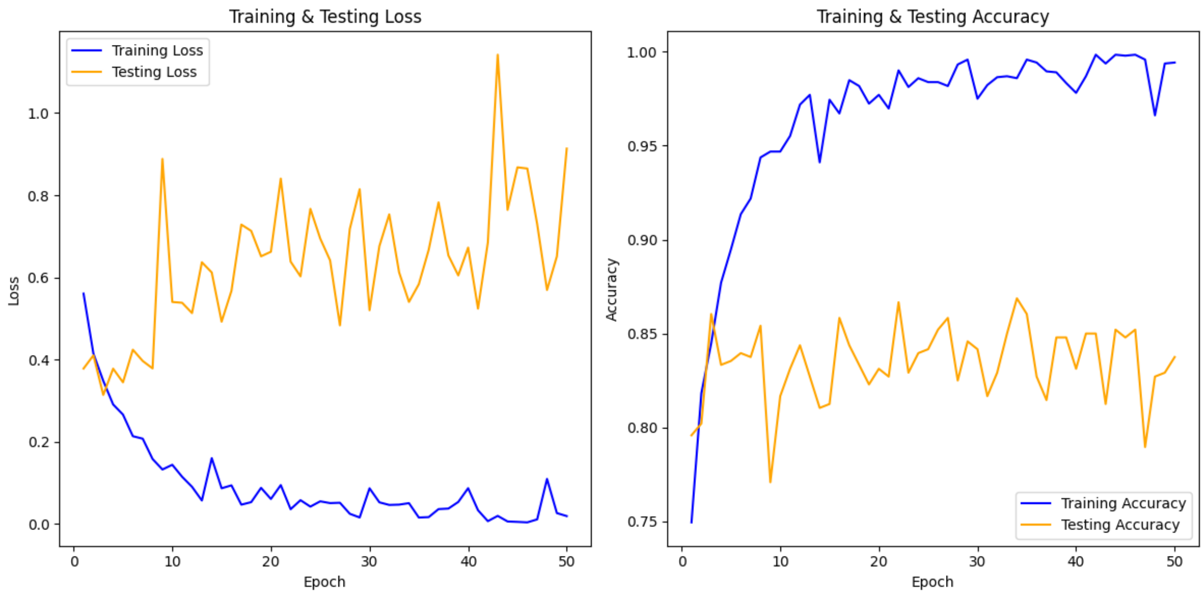

Figure S2. 7 Confusion Matrix for Actual and Predicted FMA values classified by DenseNet201

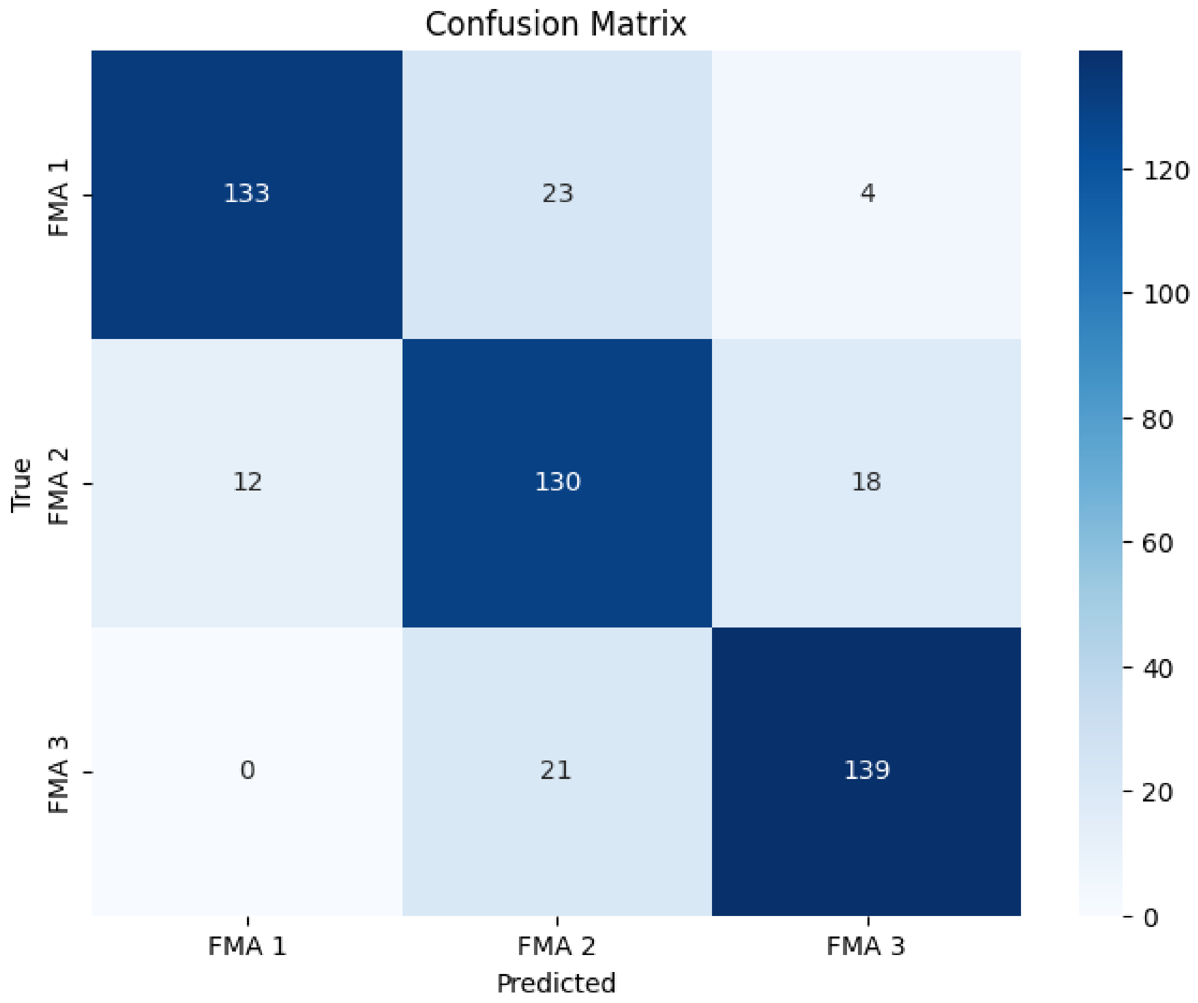

Figure S2. 8 AUC-ROC curve for FMA classified by DenseNet201

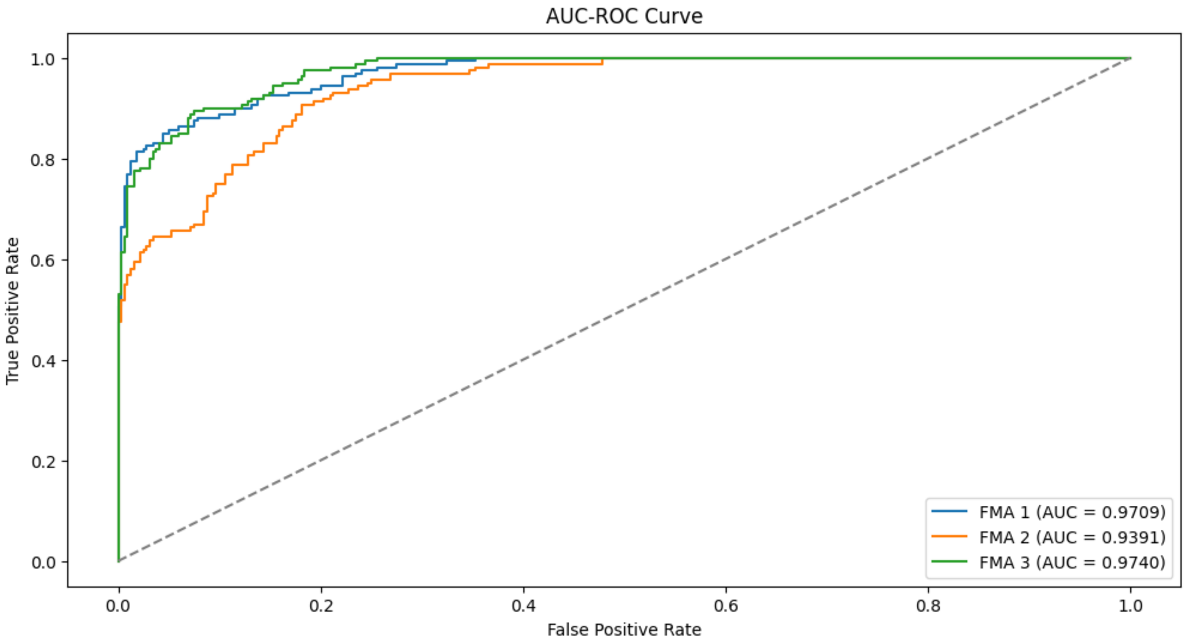

Figure S2. 9 Precision–recall curve for FMA classified by DenseNet201

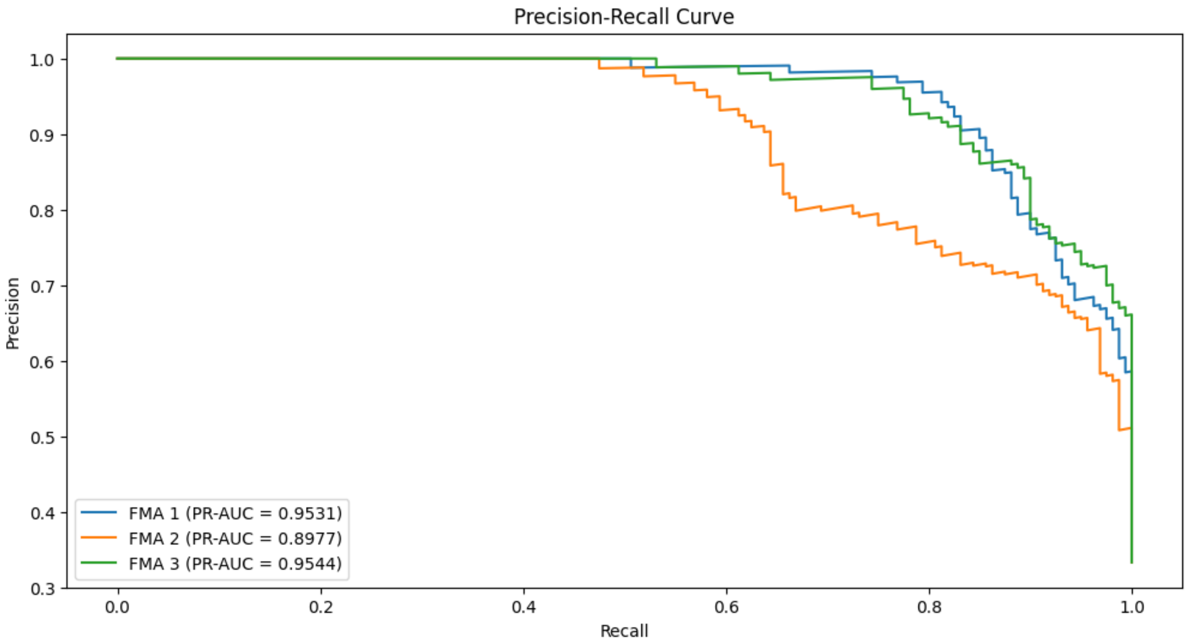

Table S2. 2 Classification Report for FMA by DenseNet201

```
Mean Absolute Error (MAE): 0.1708
Cohen's Kappa: 0.7562
Classification Report:
              precision    recall  f1-score   support

       FMA 1     0.9172    0.8313    0.8721       160
       FMA 2     0.7471    0.8125    0.7784       160
       FMA 3     0.8634    0.8688    0.8660       160

    accuracy                         0.8375       480
   macro avg     0.8426    0.8375    0.8389       480
weighted avg     0.8426    0.8375    0.8389       480
```

Figure S2. 10 The original and Grad-CAM Images for FMA Generated by DenseNet201

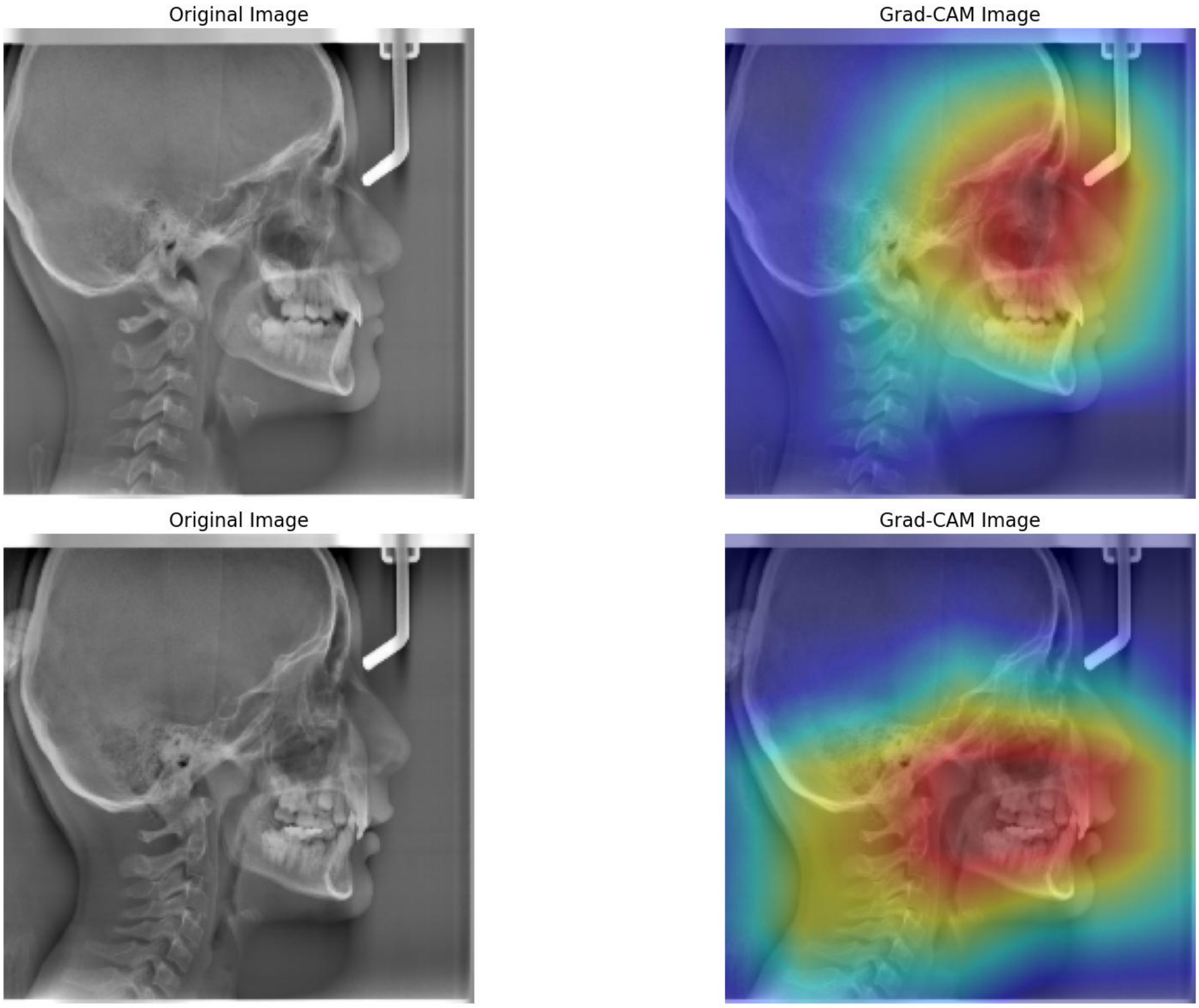

2.3 Classification of FMA by EfficientNet B0

Figure S2. 11 Training and Testing Loss and Training and Testing Accuracy Graphs for EfficientNet B0

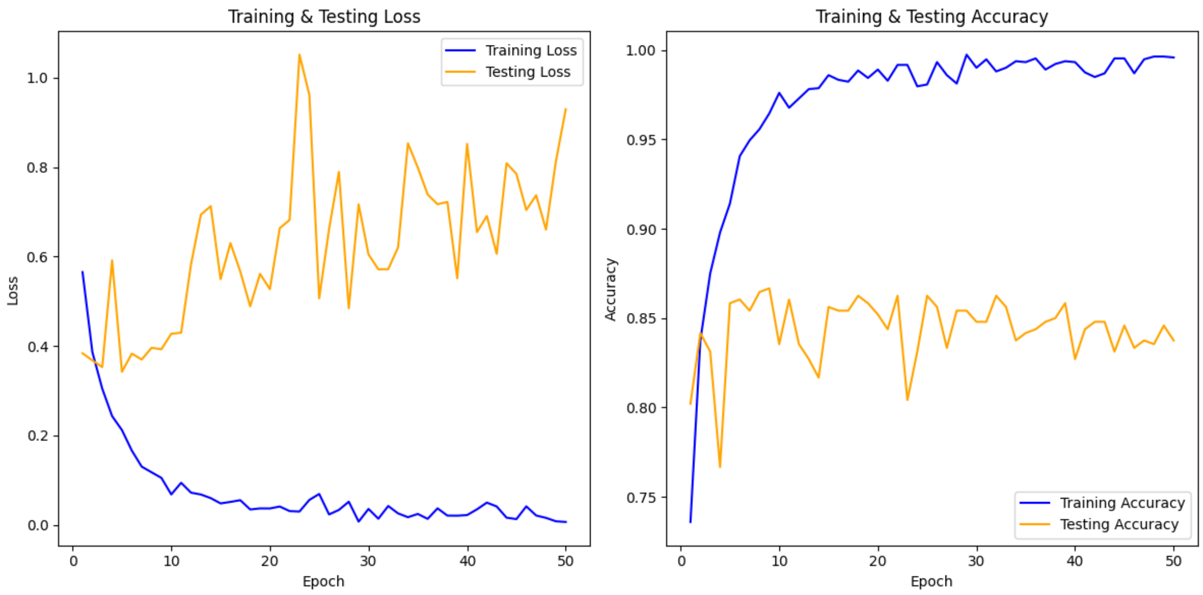

Figure S2. 12 Confusion Matrix for Actual and Predicted FMA values classified by EfficientNet B0

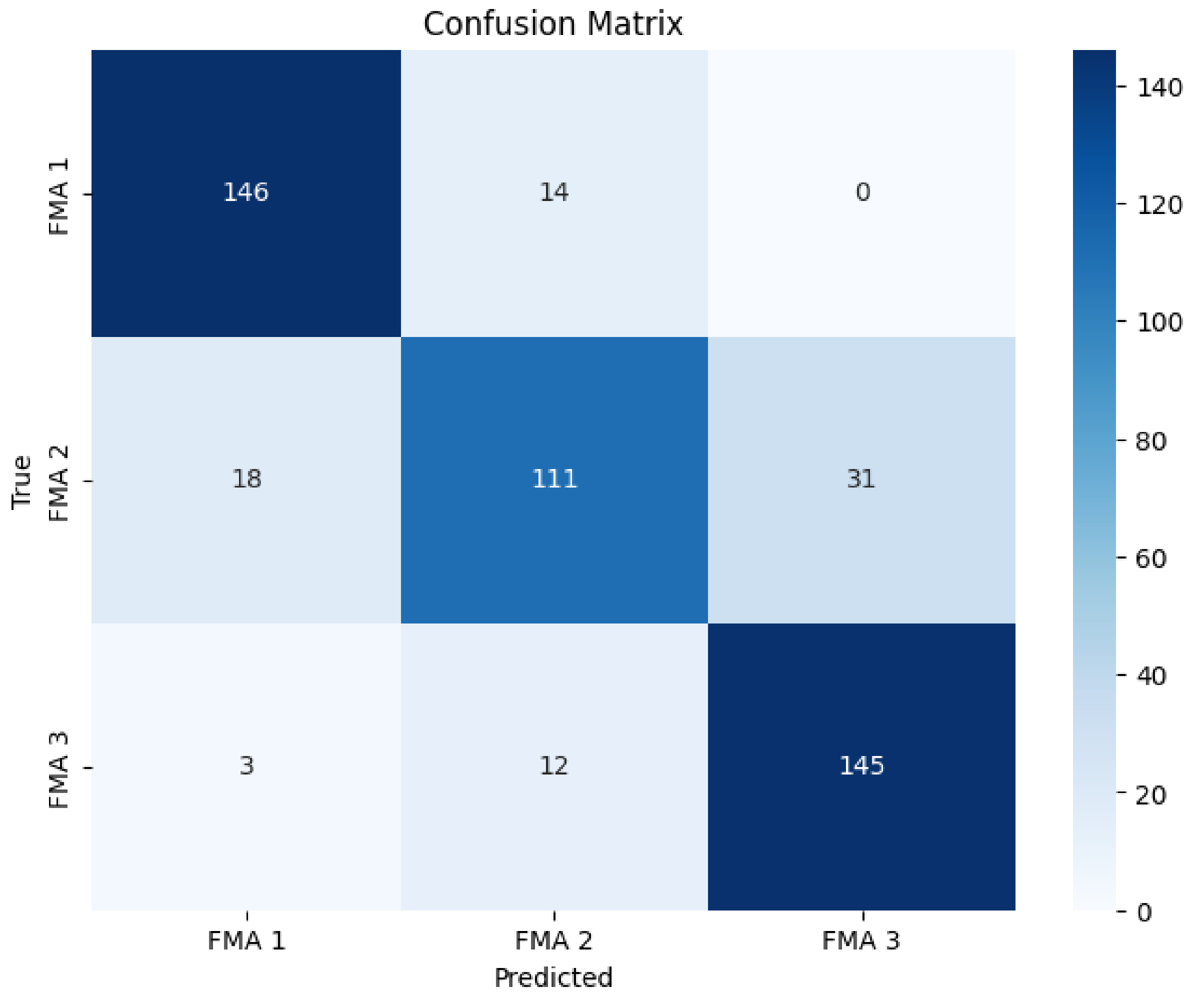

Figure S2. 13 AUC-ROC curve for FMA classified by EfficientNet B0

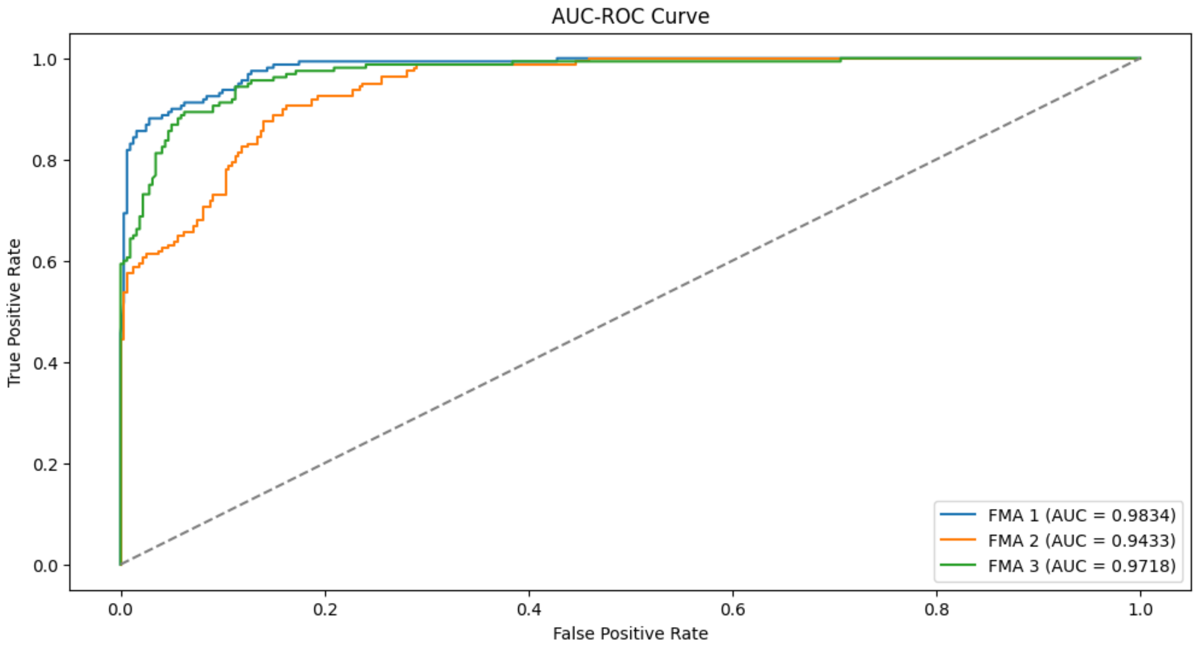

Figure S2. 14 Precision–recall curve for FMA classified by EfficientNet B0

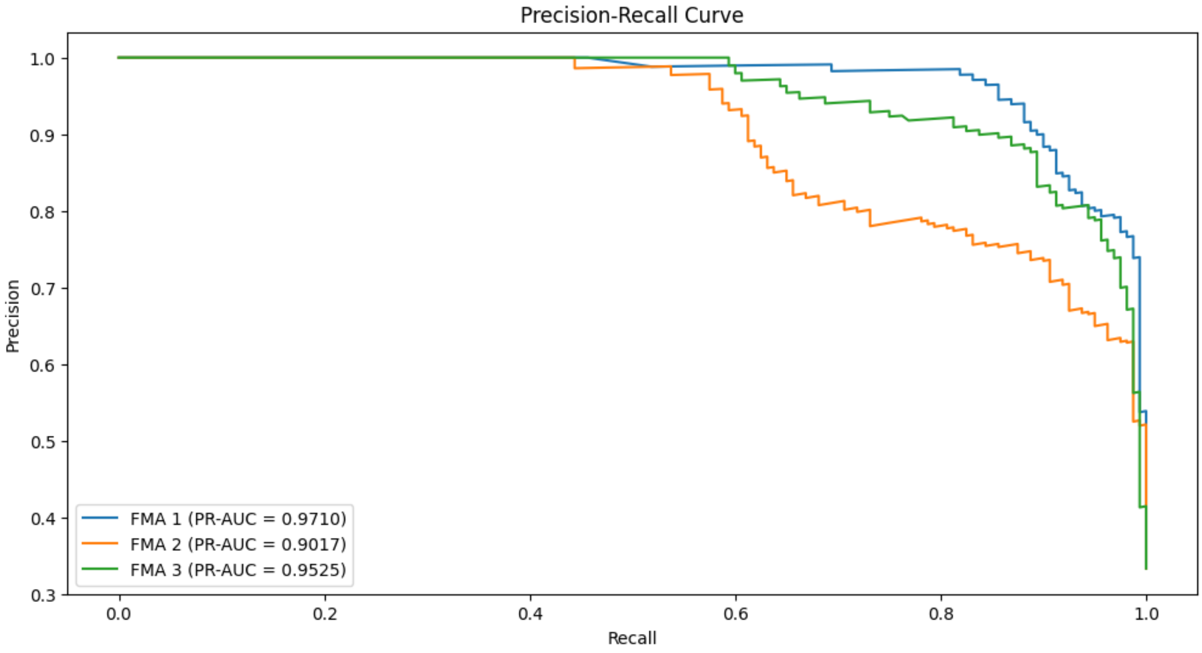

Table S2. 3 Classification Report for FMA by EfficientNet B0

Mean Absolute Error (MAE): 0.1688
Cohen's Kappa: 0.7562
Classification Report:

	precision	recall	f1-score	support
FMA 1	0.8743	0.9125	0.8930	160
FMA 2	0.8102	0.6937	0.7475	160
FMA 3	0.8239	0.9062	0.8631	160
accuracy			0.8375	480
macro avg	0.8361	0.8375	0.8345	480
weighted avg	0.8361	0.8375	0.8345	480

Figure S2. 15 The original and Grad-CAM Images for FMA Generated by EfficientNet B0

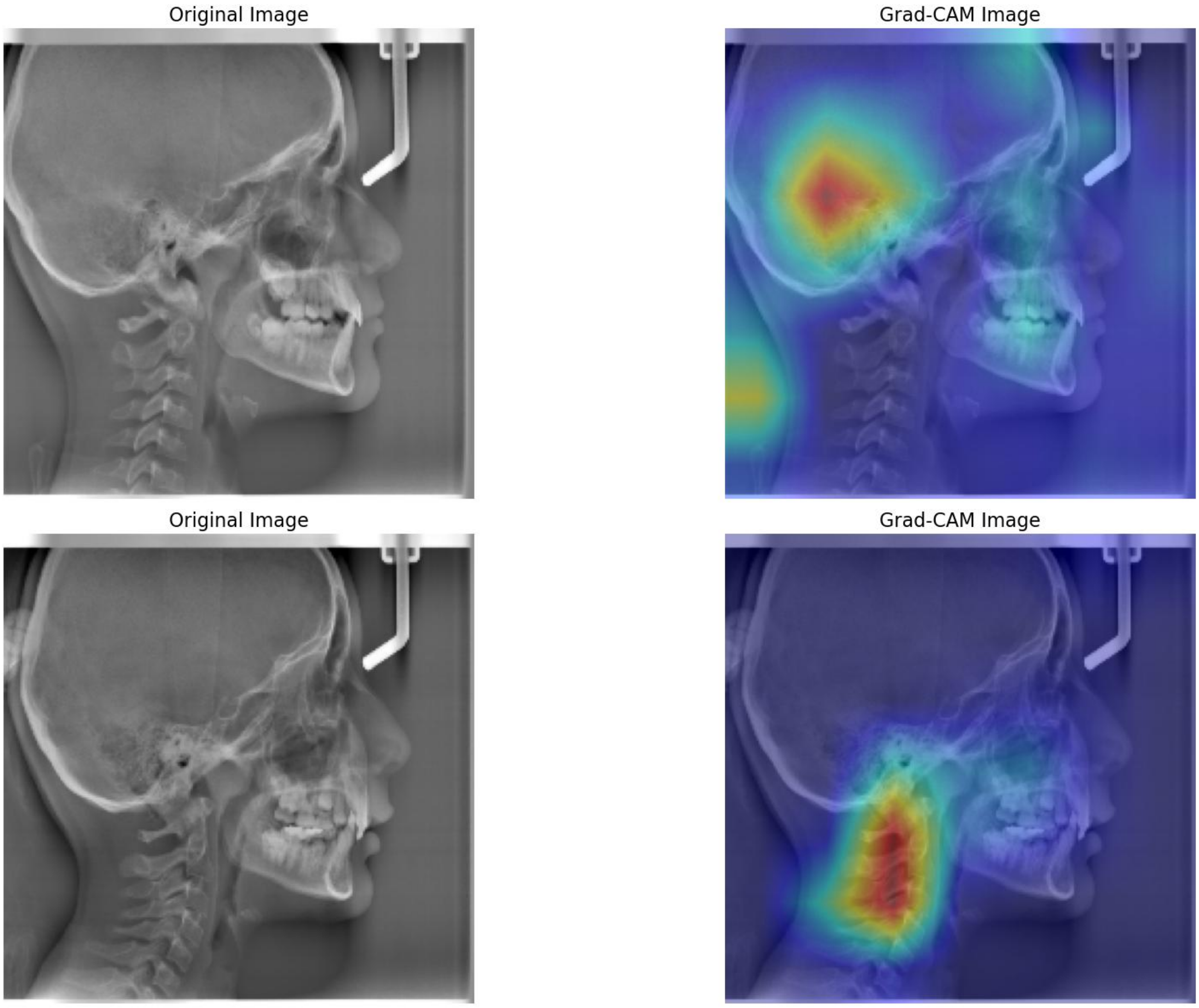

2.4 Classification of FMA by EfficientNet V2

Figure S2. 16 Training and Testing Loss and Training and Testing Accuracy Graphs for EfficientNet V2

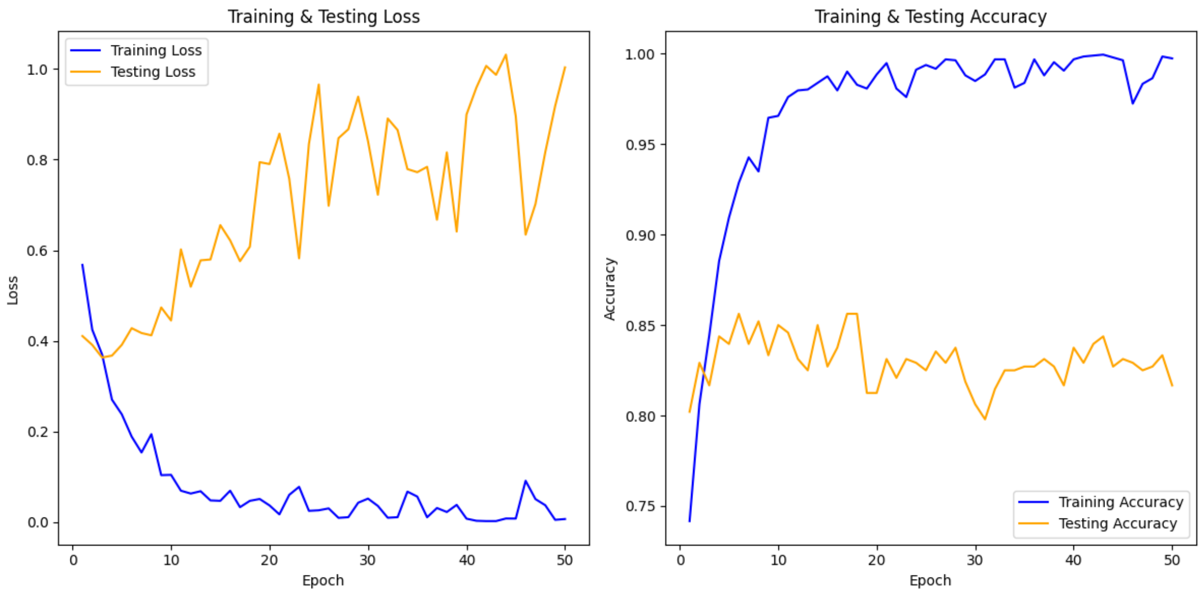

Figure S2. 17 Confusion Matrix for Actual and Predicted FMA values classified by EfficientNet V2

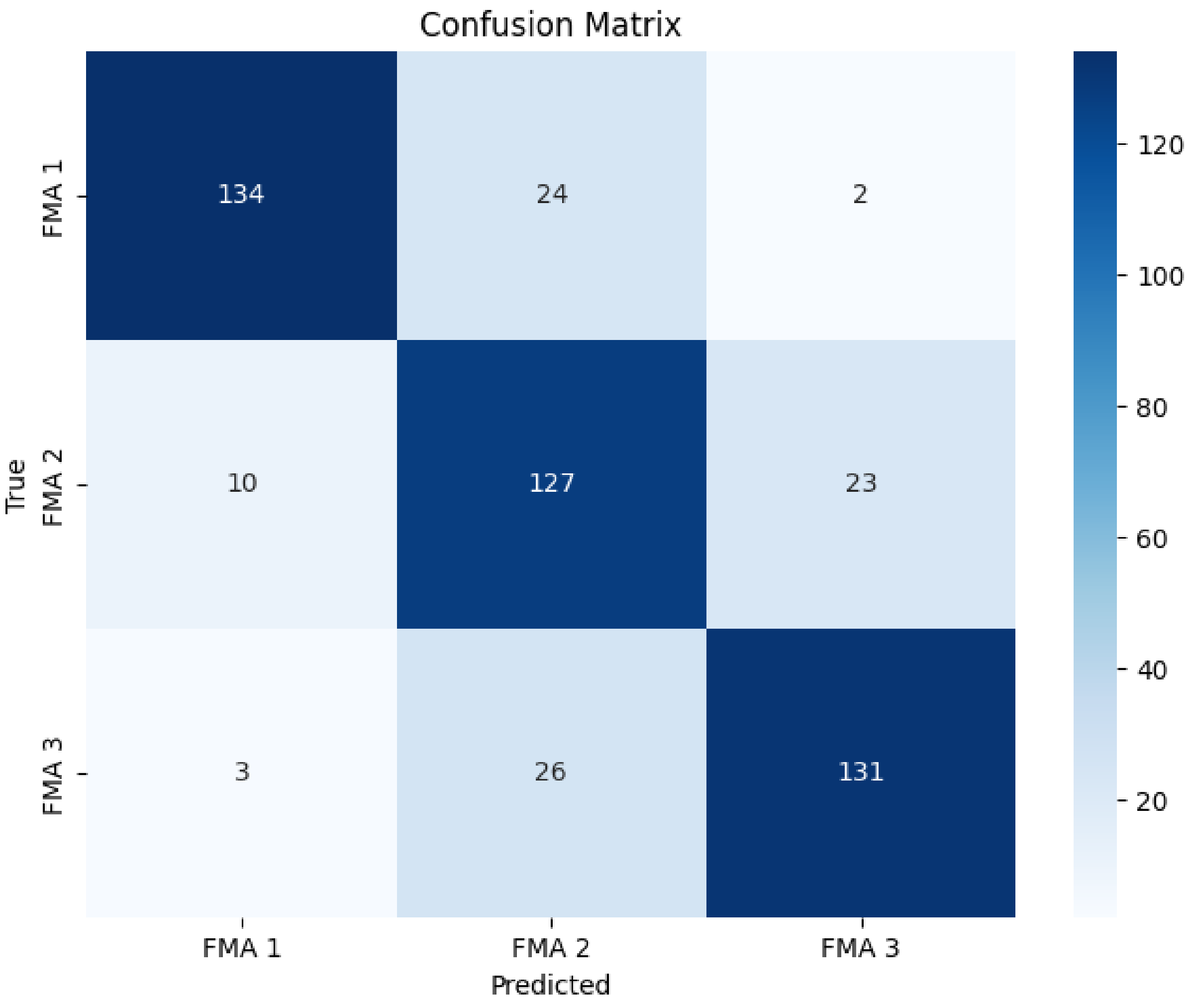

Figure S2. 18 AUC-ROC curve for FMA classified by EfficientNet V2

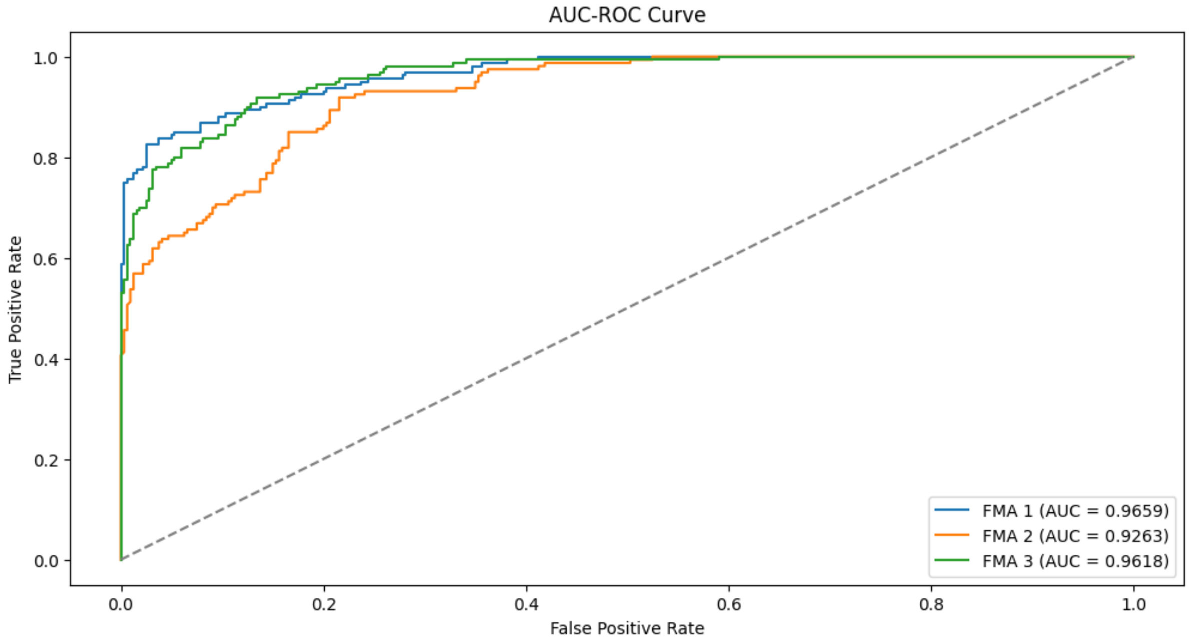

Figure S2. 19 Precision–recall curve for FMA classified by EfficientNet V2

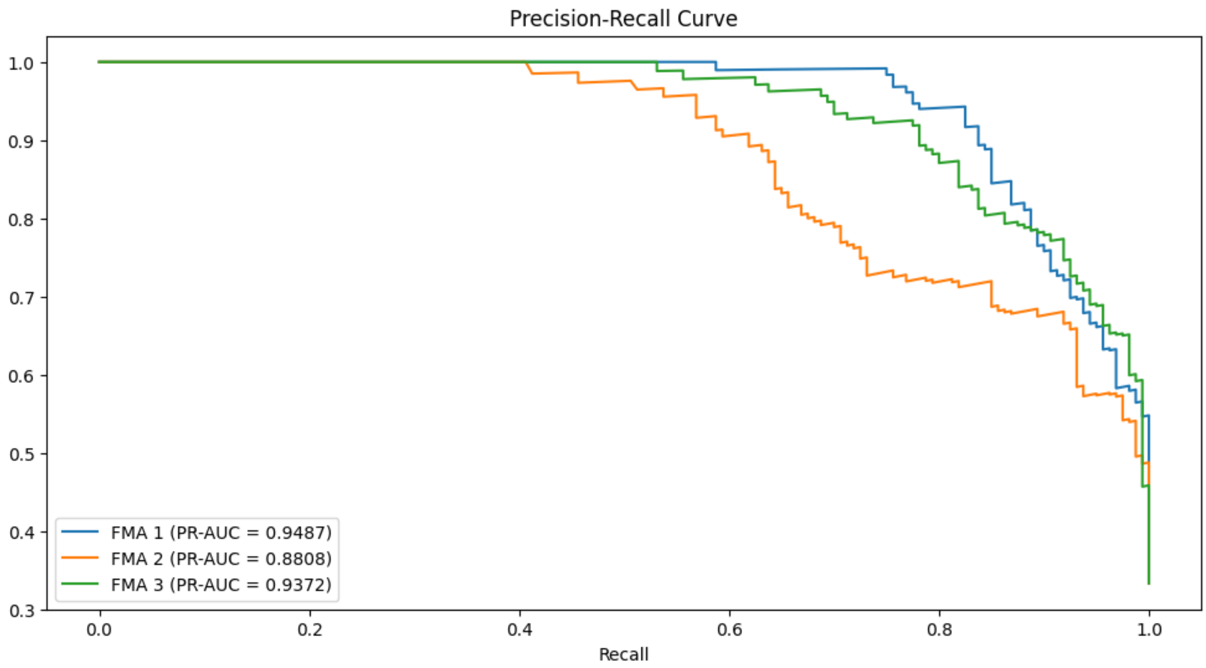

Table S2. 4 Classification Report for FMA by EfficientNet V2

Mean Absolute Error (MAE): 0.1938
Cohen's Kappa: 0.7250
Classification Report:

	precision	recall	f1-score	support
FMA 1	0.9116	0.8375	0.8730	160
FMA 2	0.7175	0.7937	0.7537	160
FMA 3	0.8397	0.8187	0.8291	160
accuracy			0.8167	480
macro avg	0.8229	0.8167	0.8186	480
weighted avg	0.8229	0.8167	0.8186	480

Figure S2. 20 The original and Grad-CAM Images for FMA Generated by EfficientNet V2

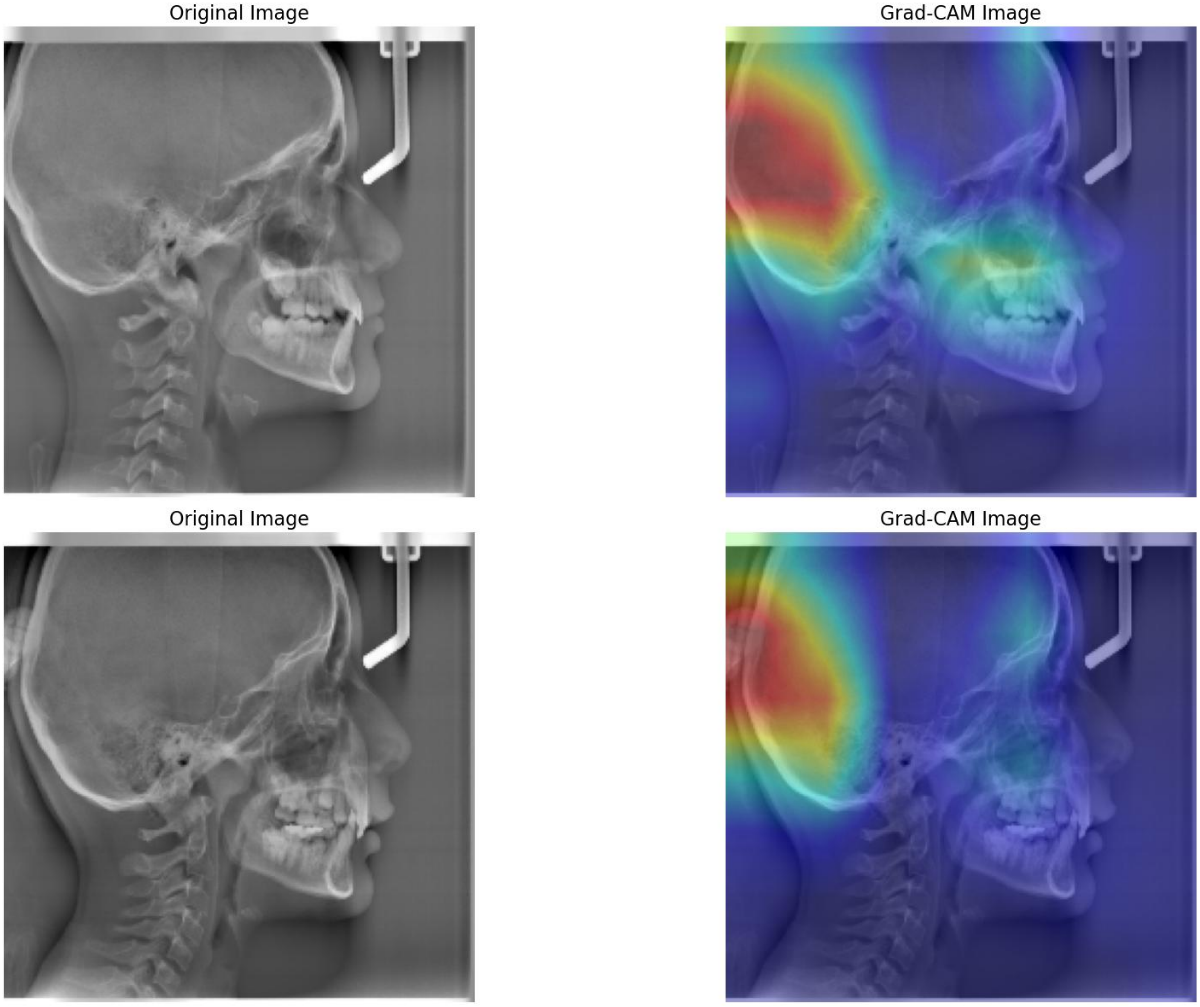

2.5 Classification of FMA by Hybrid Algorithm

Figure S2. 21 Training and Testing Loss and Training and Testing Accuracy Graphs for Hybrid Algorithm

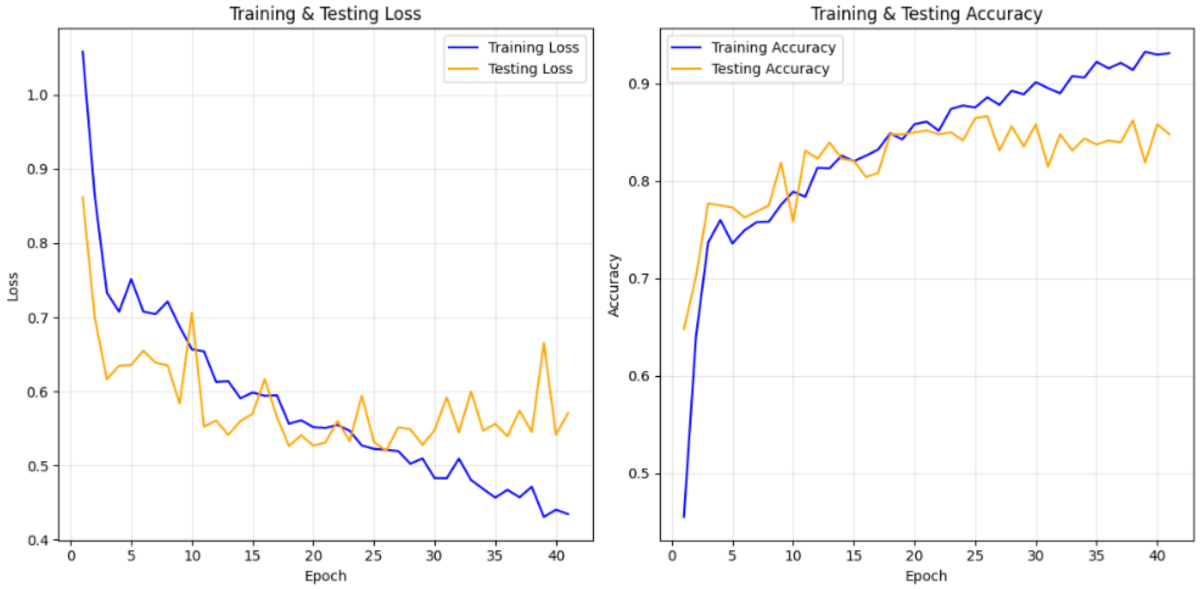

Figure S2. 22 Confusion Matrix for Actual and Predicted FMA values classified by Hybrid Algorithm

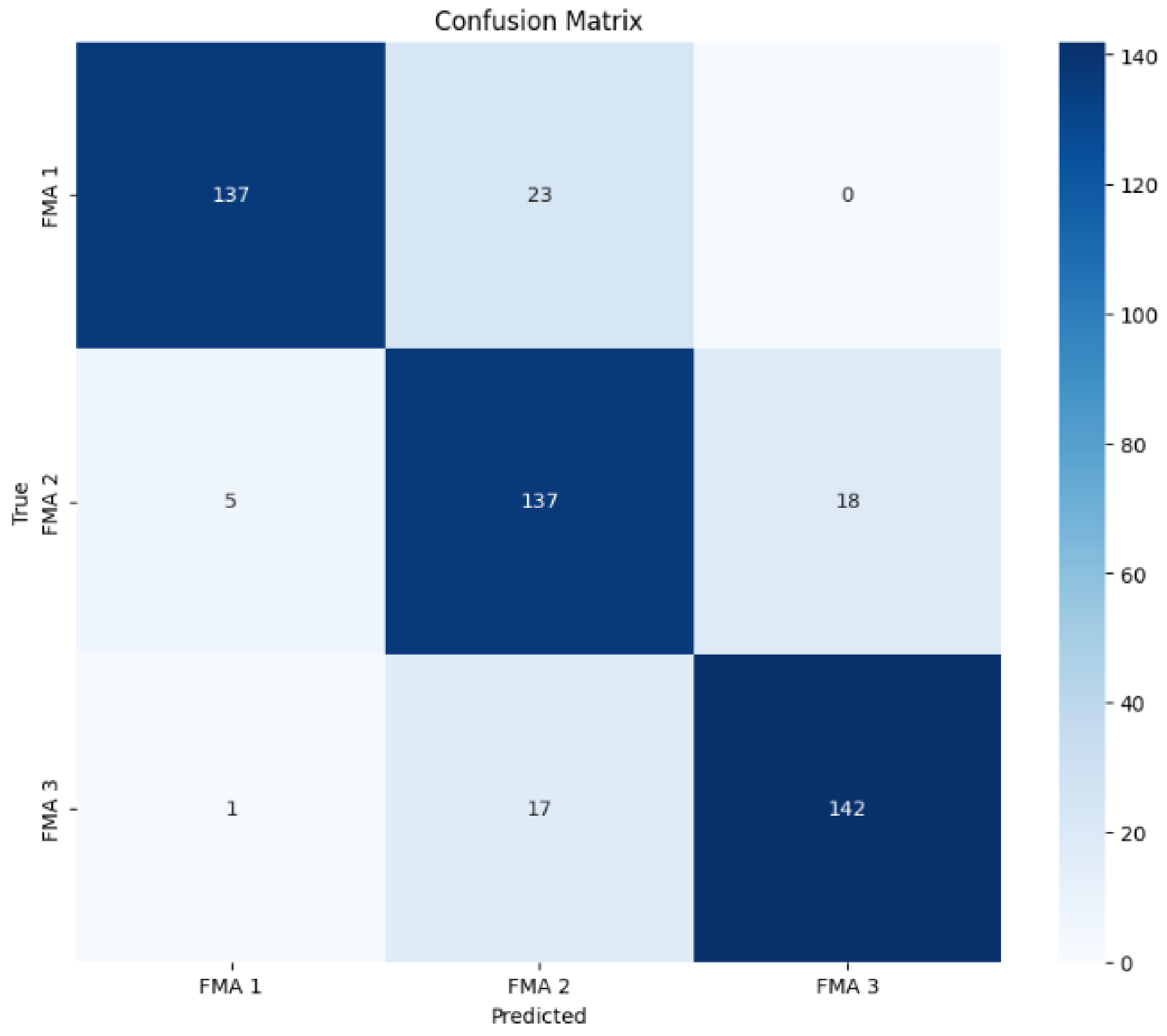

Figure S2. 23 AUC-ROC curve for FMA classified by Hybrid Algorithm

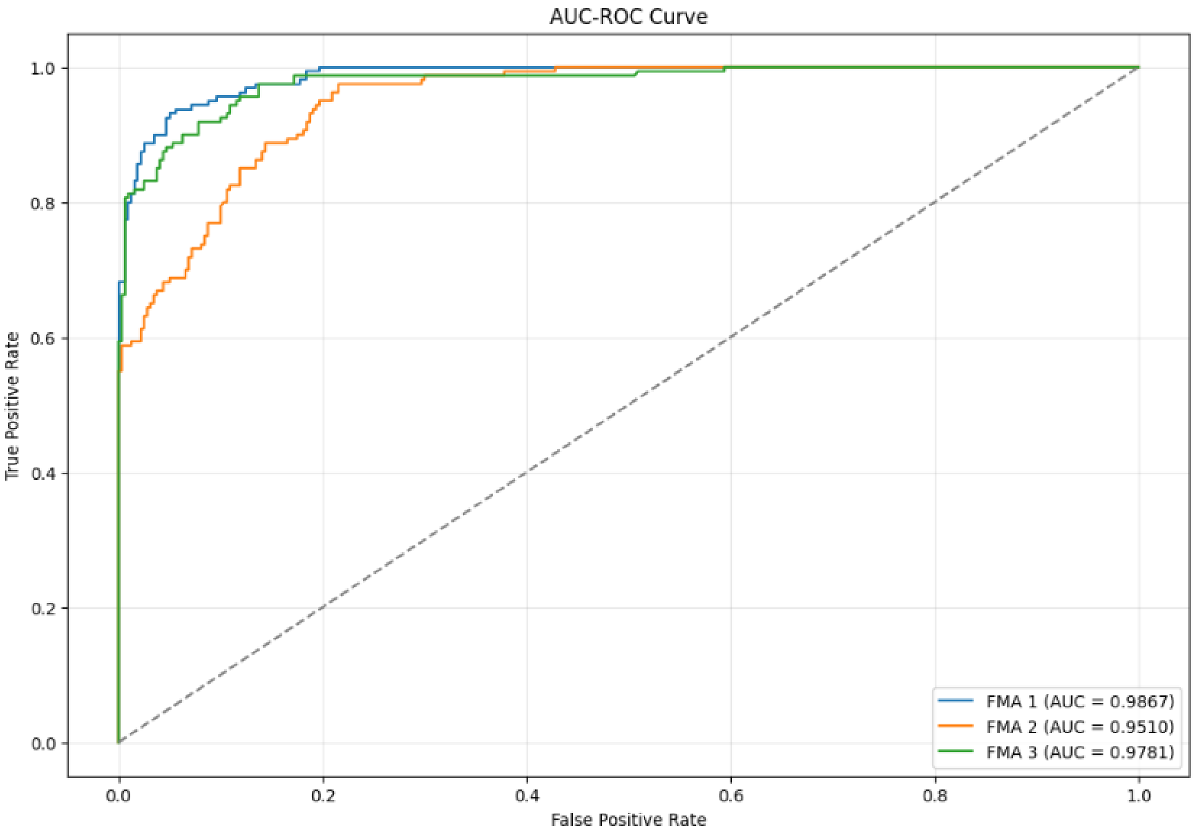

Figure S2. 24 Precision–recall curve for FMA classified by Hybrid Algorithm

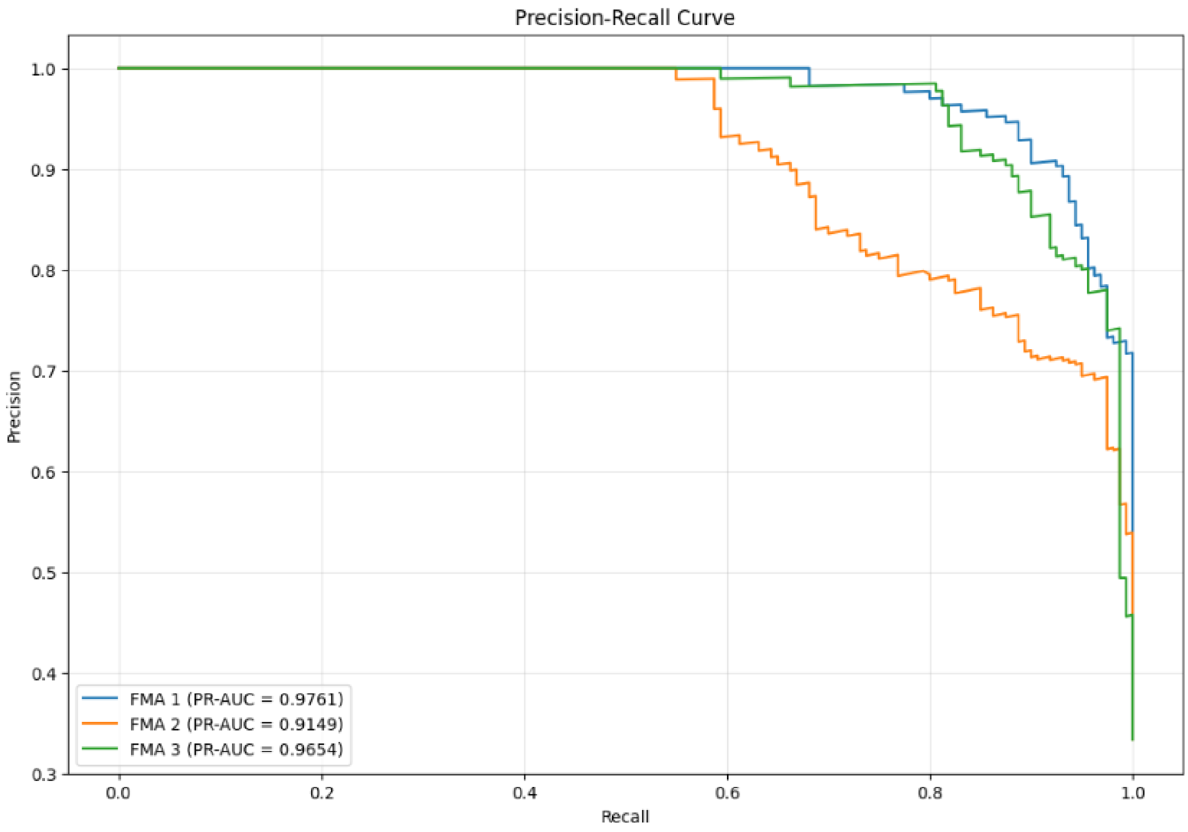

Table S2. 5 Classification Report for FMA by Hybrid Algorithm

Mean Absolute Error (MAE): 0.1354
Cohen's Kappa: 0.8000
Classification Report:

	precision	recall	f1-score	support
FMA 1	0.9580	0.8562	0.9043	160
FMA 2	0.7740	0.8562	0.8131	160
FMA 3	0.8875	0.8875	0.8875	160
accuracy			0.8667	480
macro avg	0.8732	0.8667	0.8683	480
weighted avg	0.8732	0.8667	0.8683	480

Figure S2. 25 The original and Grad-CAM Images for FMA Generated by Hybrid Algorithm

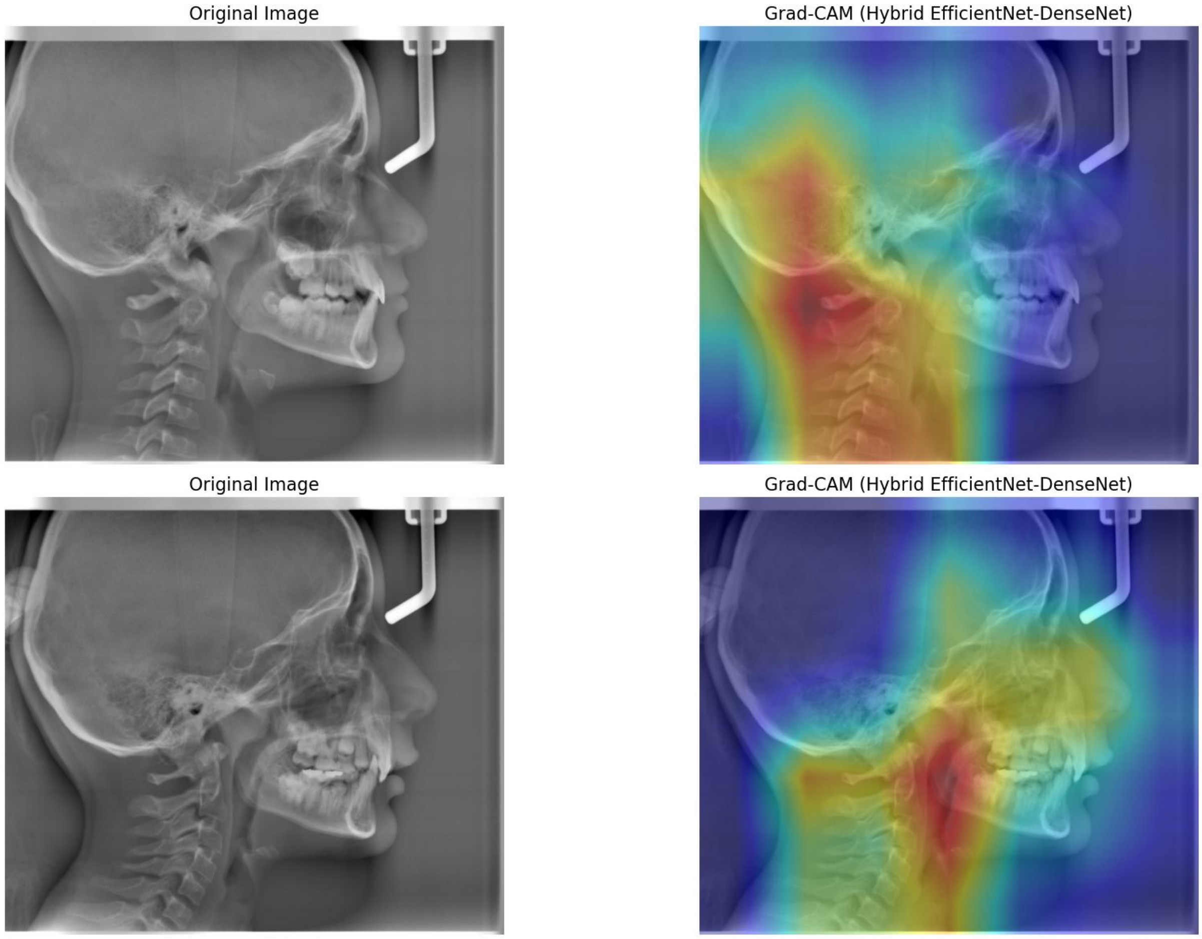

2.6 Classification of FMA by MobileNetV2

Figure S2. 26 Training and Testing Loss and Training and Testing Accuracy Graphs for MobileNetV2

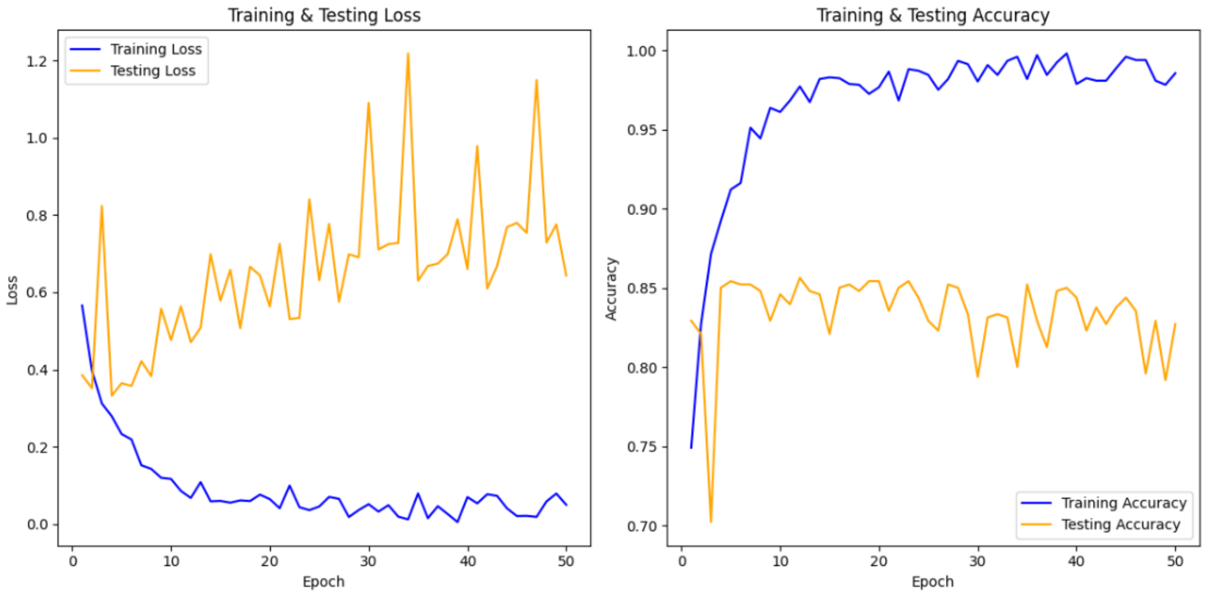

Figure S2. 27 Confusion Matrix for Actual and Predicted FMA values classified by MobileNetV2

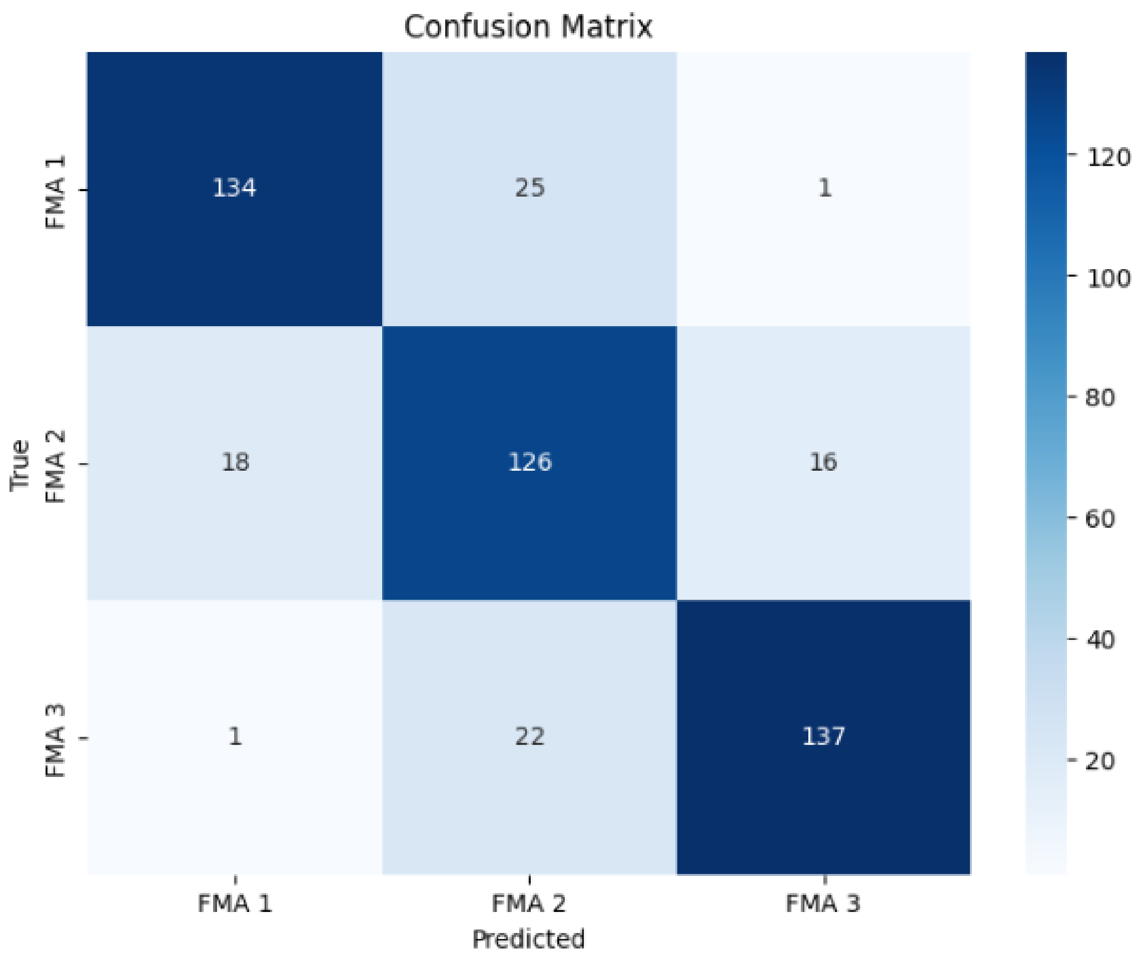

Figure S2. 28 AUC-ROC curve for FMA classified by MobileNetV2

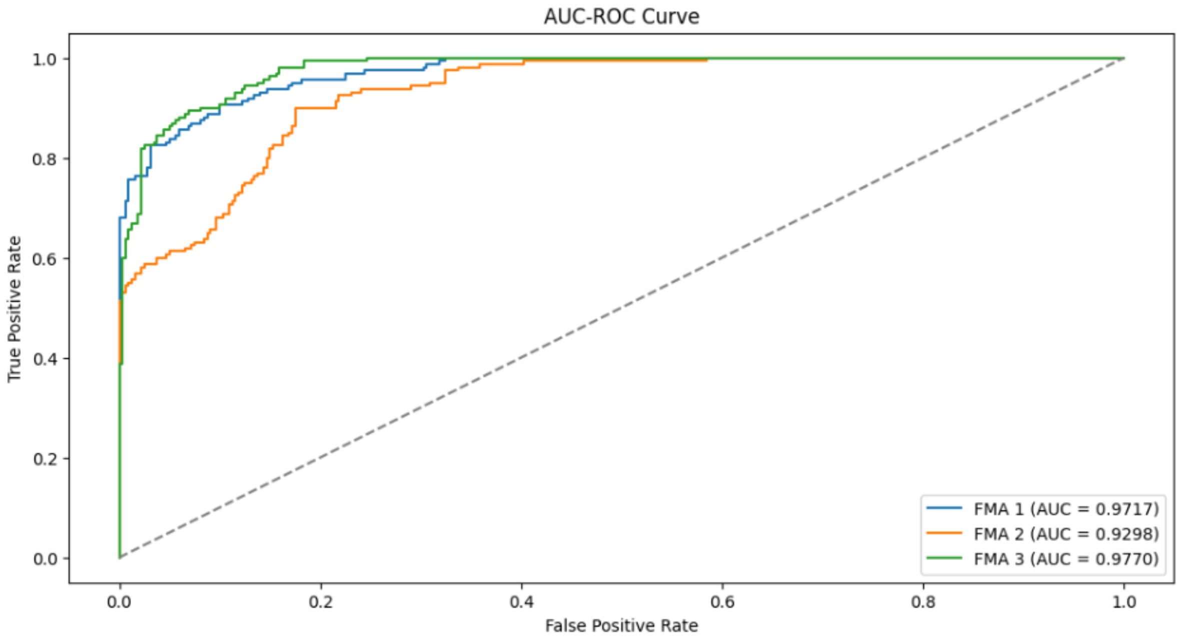

Figure S2. 29 Precision–recall curve for FMA classified by MobileNetV2

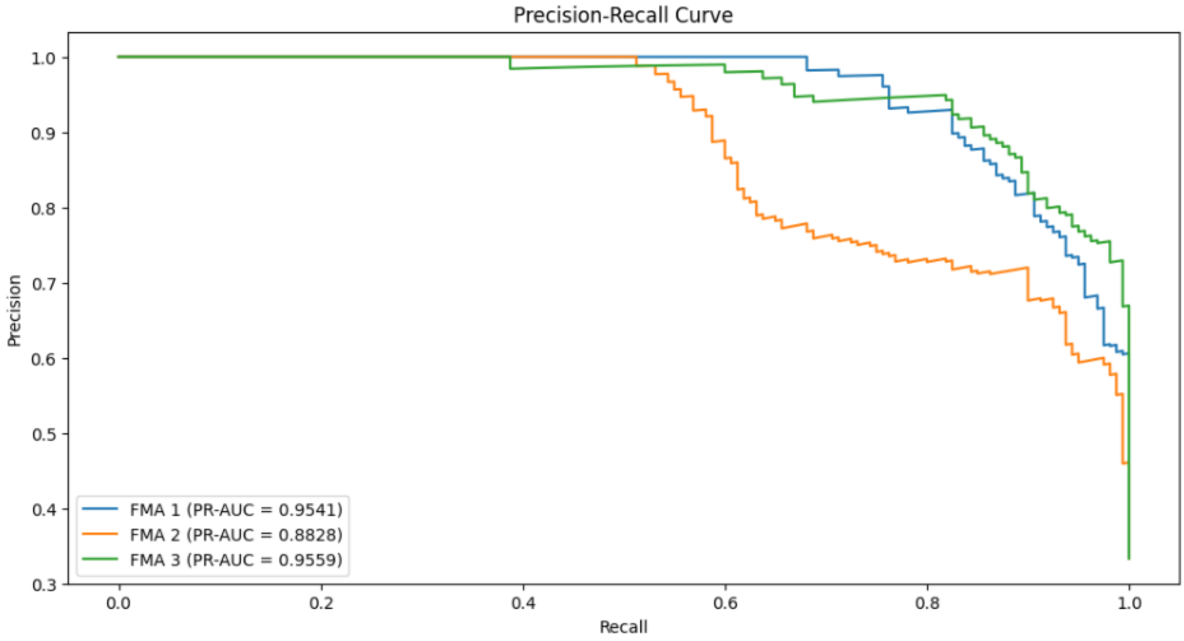

Table S2. 6 Classification Report for FMA by MobileNetV2

```
Mean Absolute Error (MAE): 0.1771
Cohen's Kappa: 0.7406
Classification Report:
              precision    recall  f1-score   support

       FMA 1     0.8758    0.8375    0.8562       160
       FMA 2     0.7283    0.7875    0.7568       160
       FMA 3     0.8896    0.8562    0.8726       160

    accuracy                         0.8271       480
   macro avg     0.8313    0.8271    0.8285       480
weighted avg     0.8313    0.8271    0.8285       480
```

Figure S1. 30 The original and Grad-CAM Images for FMA Plane Generated by MobileNetV2

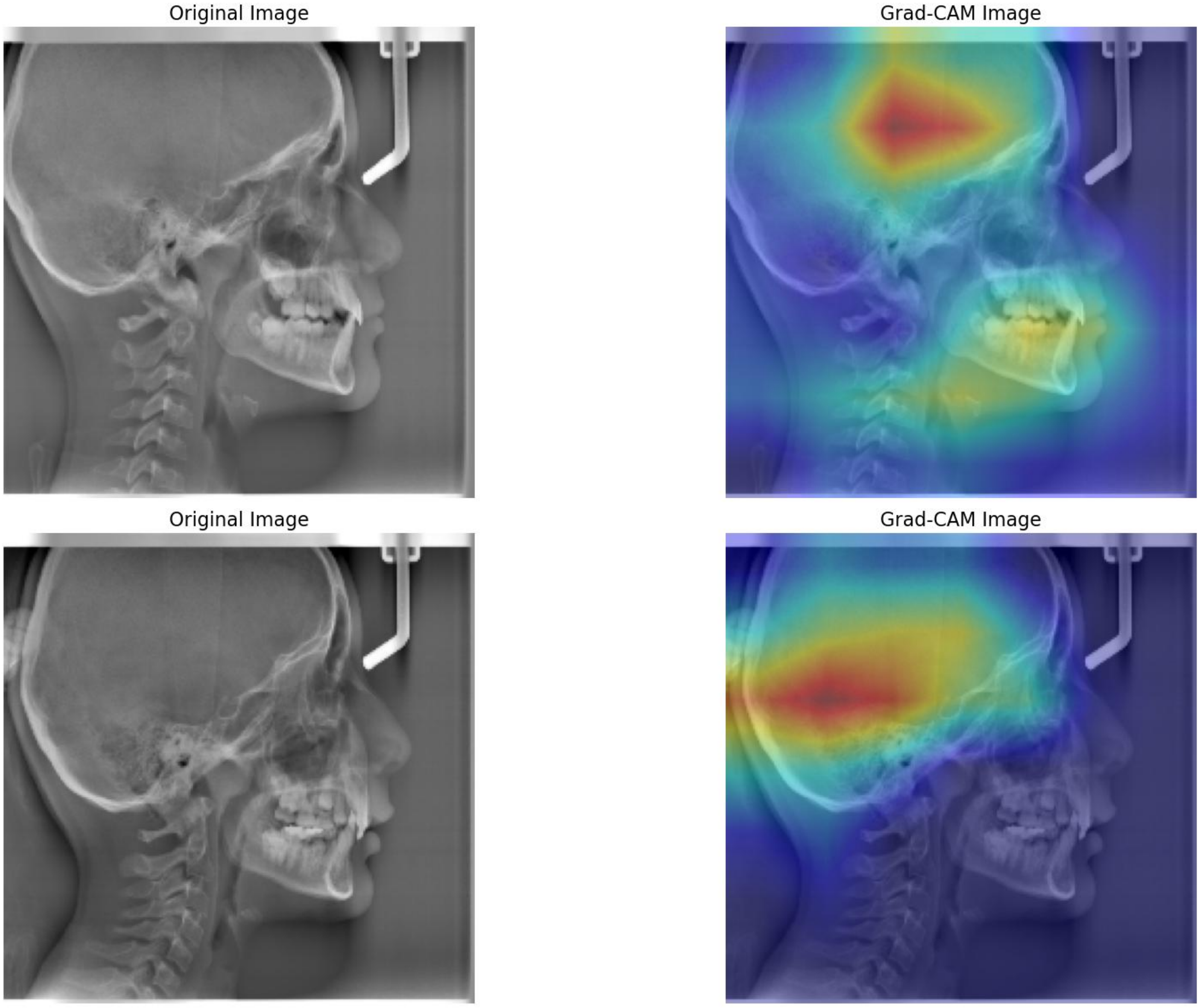

2.7 Classification of FMA by ResNet101

Figure S2. 31 Training and Testing Loss and Training and Testing Accuracy Graphs for ResNet101

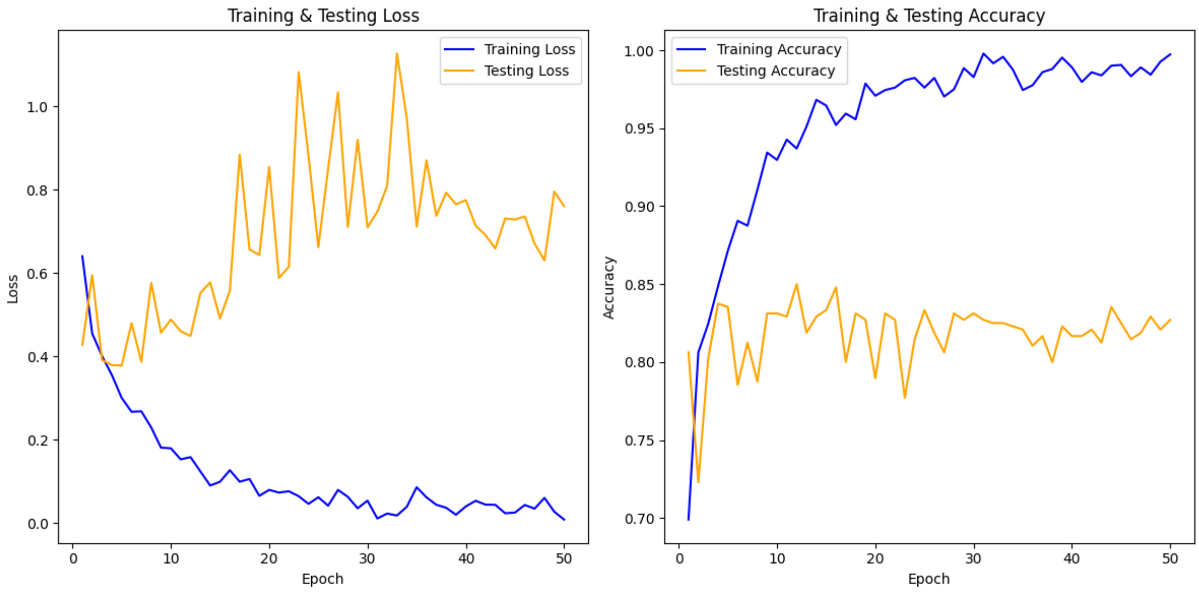

Figure S2. 32 Confusion Matrix for Actual and Predicted FMA values classified by ResNet101

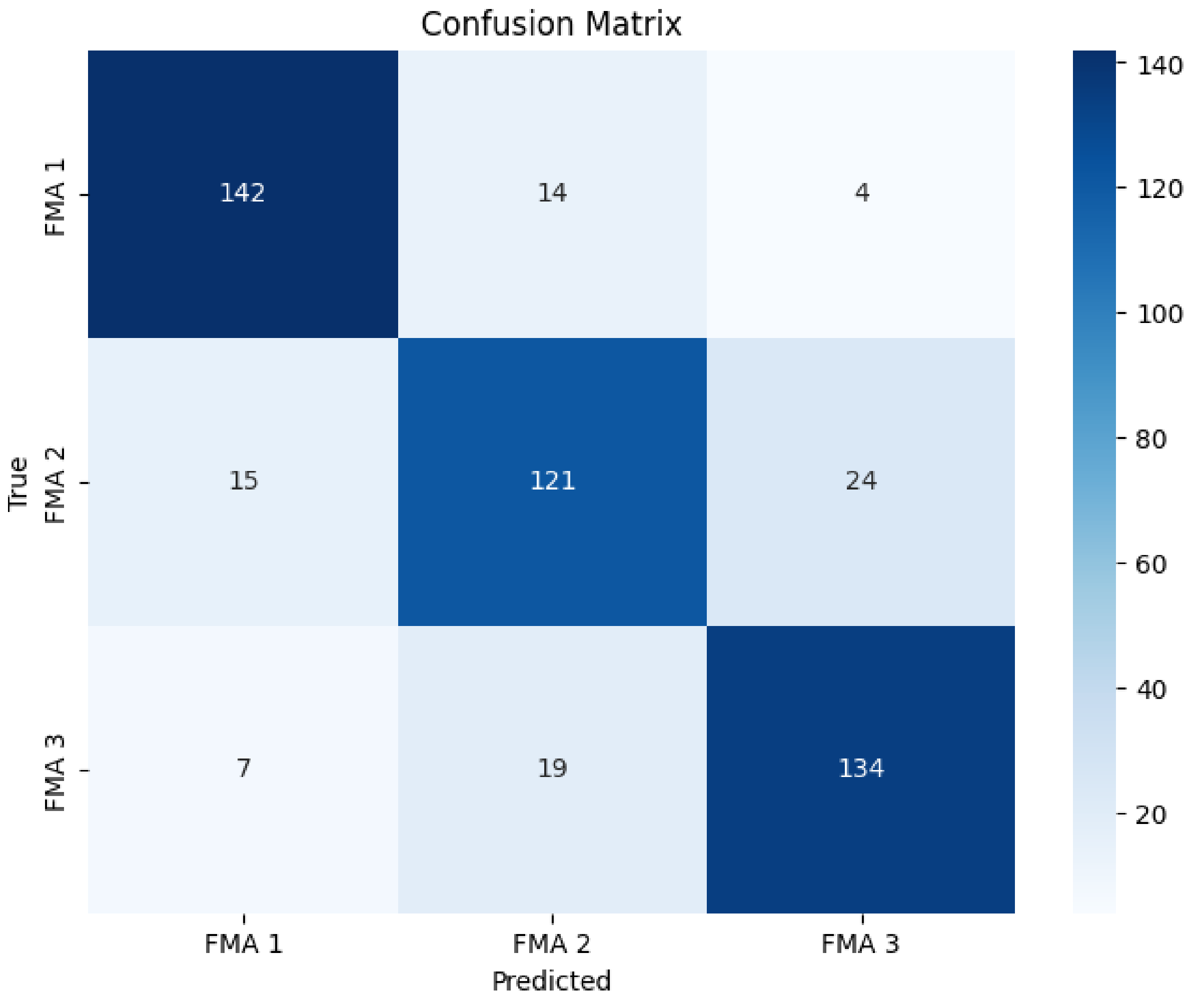

Figure S2. 33 AUC-ROC curve for FMA classified by ResNet101

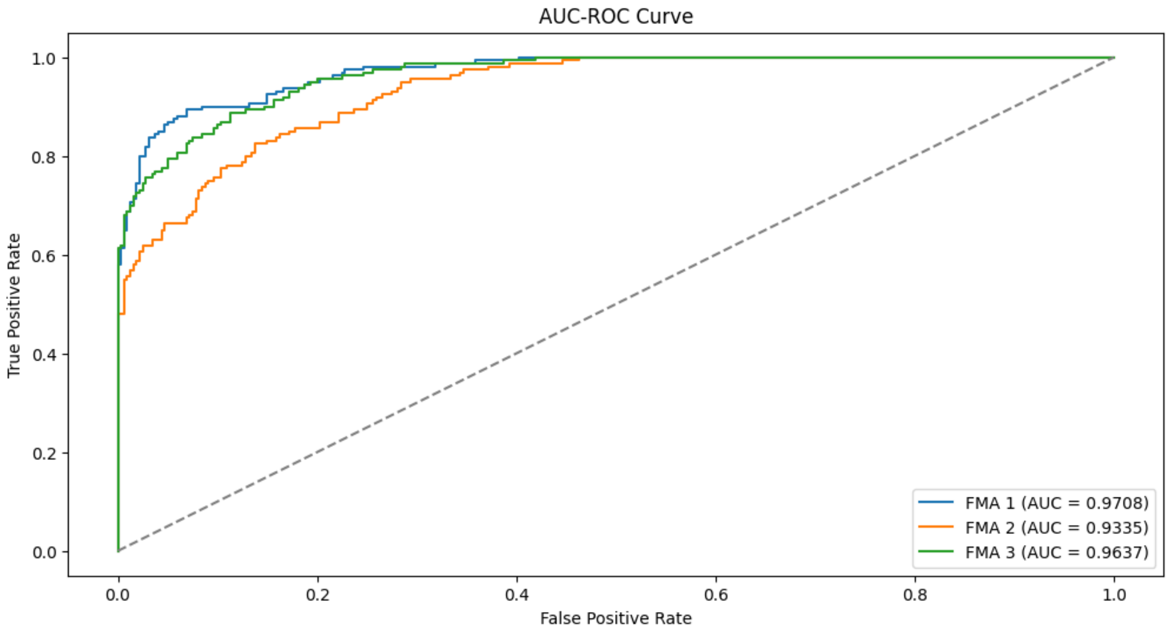

Figure S2. 34 Precision–recall curve for FMA classified by ResNet101

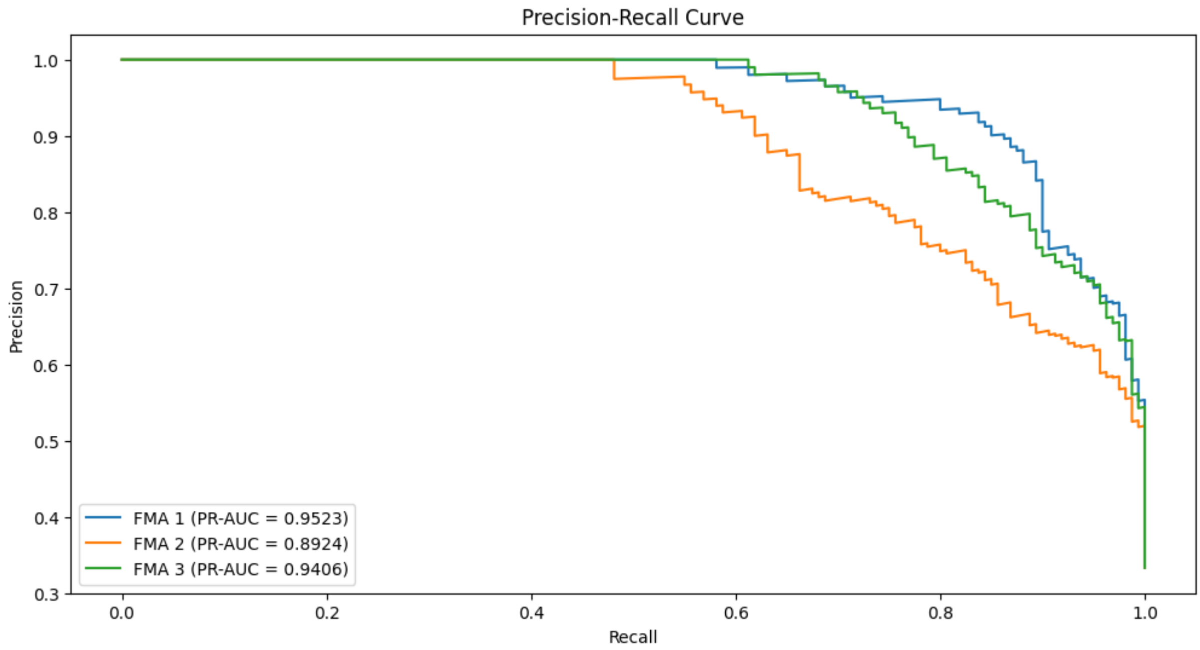

Table S2. 7 Classification Report for FMA by ResNet101

Mean Absolute Error (MAE): 0.1958
Cohen's Kappa: 0.7406
Classification Report:

	precision	recall	f1-score	support
FMA 1	0.8659	0.8875	0.8765	160
FMA 2	0.7857	0.7562	0.7707	160
FMA 3	0.8272	0.8375	0.8323	160
accuracy			0.8271	480
macro avg	0.8262	0.8271	0.8265	480
weighted avg	0.8262	0.8271	0.8265	480

Figure S2. 35 The original and Grad-CAM Images for FMA Generated by ResNet101

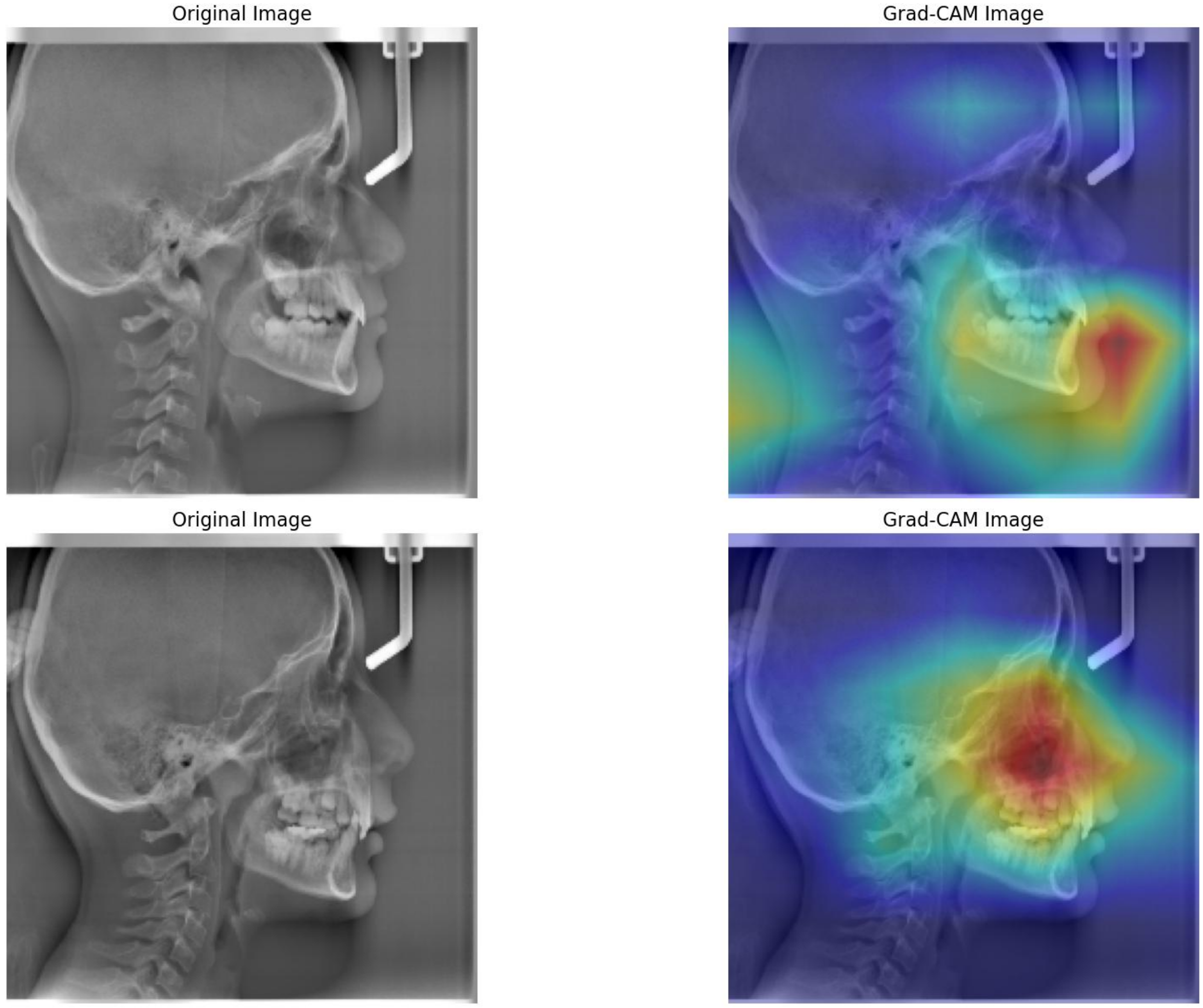

Figure S2. 36 Comparisons of Models According to Accuracy , Mean Absolute Error and Cohen's Kappa for FMA

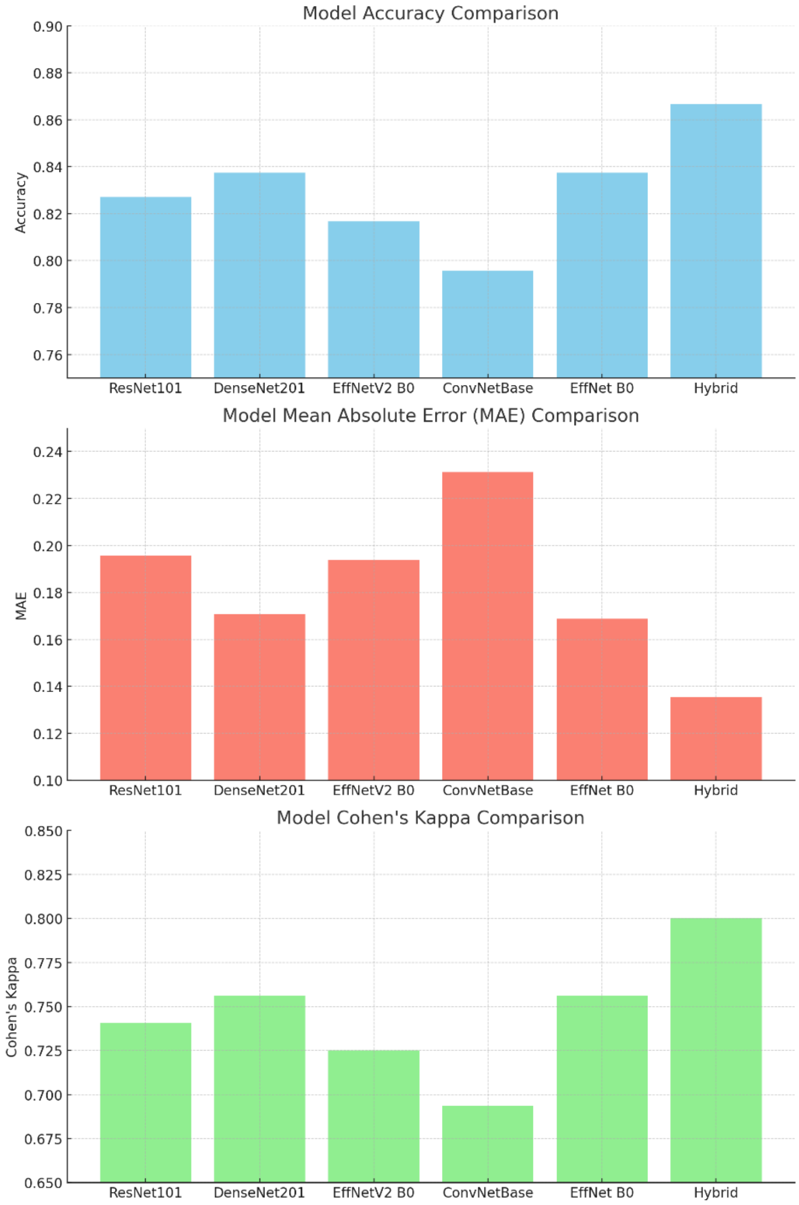

Supplement: Supplementary file 1 [file diagnostics-15-02240-s001.zip › 2 FMA.pdf]
