# Supplementary material for: Classification Performance of Deep Learning Models for the Assessment of Vertical Dimension on Lateral Cephalometric Radiographs
Source: Diagnostics (Basel). 2025 Sep 3;15(17):2240. doi: 10.3390/diagnostics15172240 (PMC12428445; doi:10.3390/diagnostics15172240)

3.1 Classification of SN-GoGn by ConvNet

Figure S3. 1 Training and Testing Loss and Training and Testing Accuracy Graphs for ConvNet

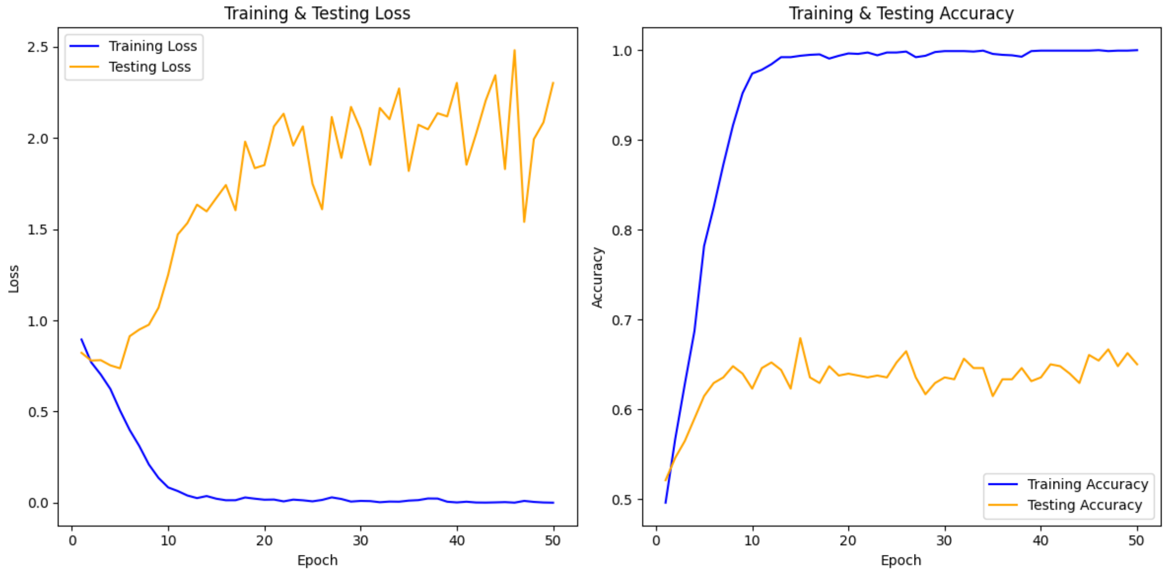

Figure S3. 2 Confusion Matrix for Actual and Predicted SN-GoGn values classified by ConvNet

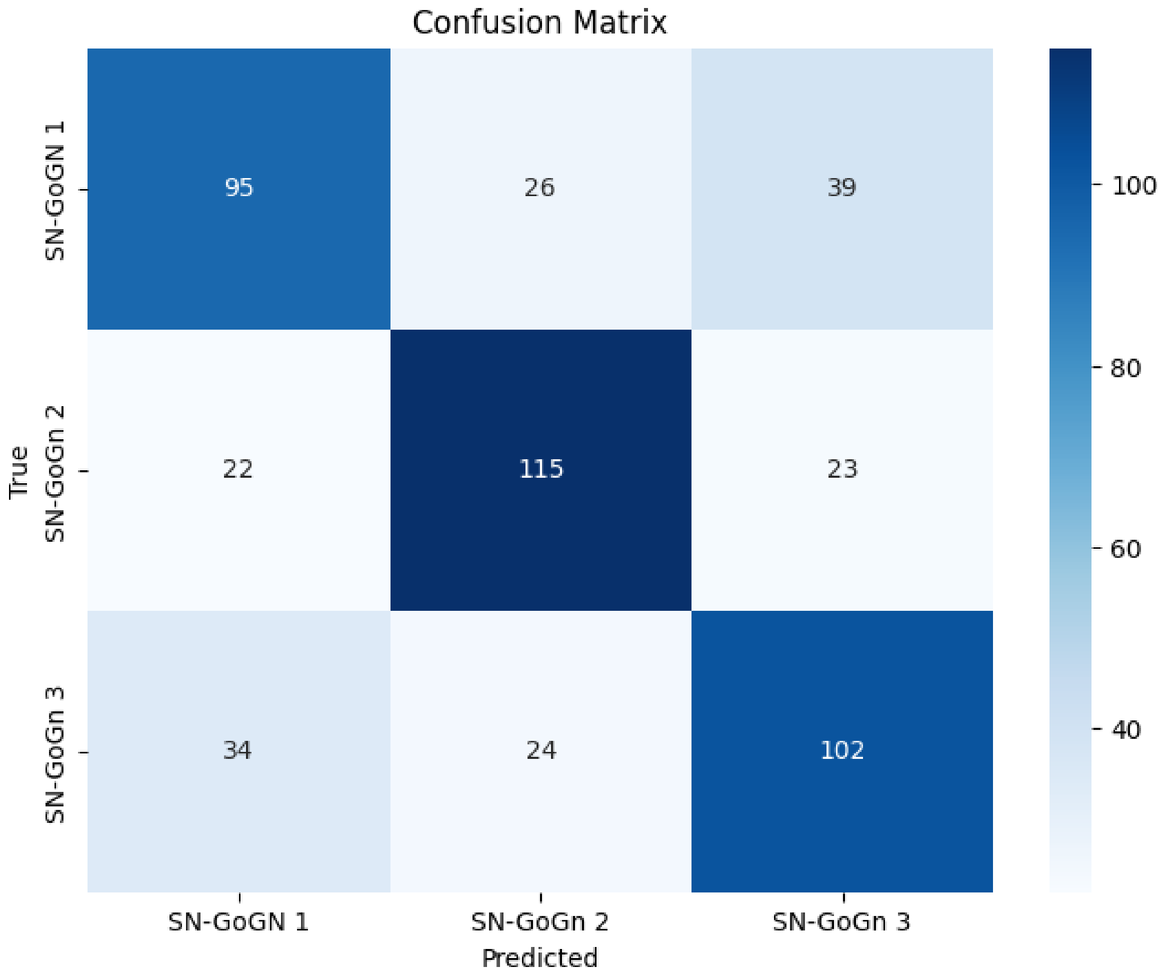

Figure S3. 3 AUC-ROC curve for SN-GoGn classified by ConvNet

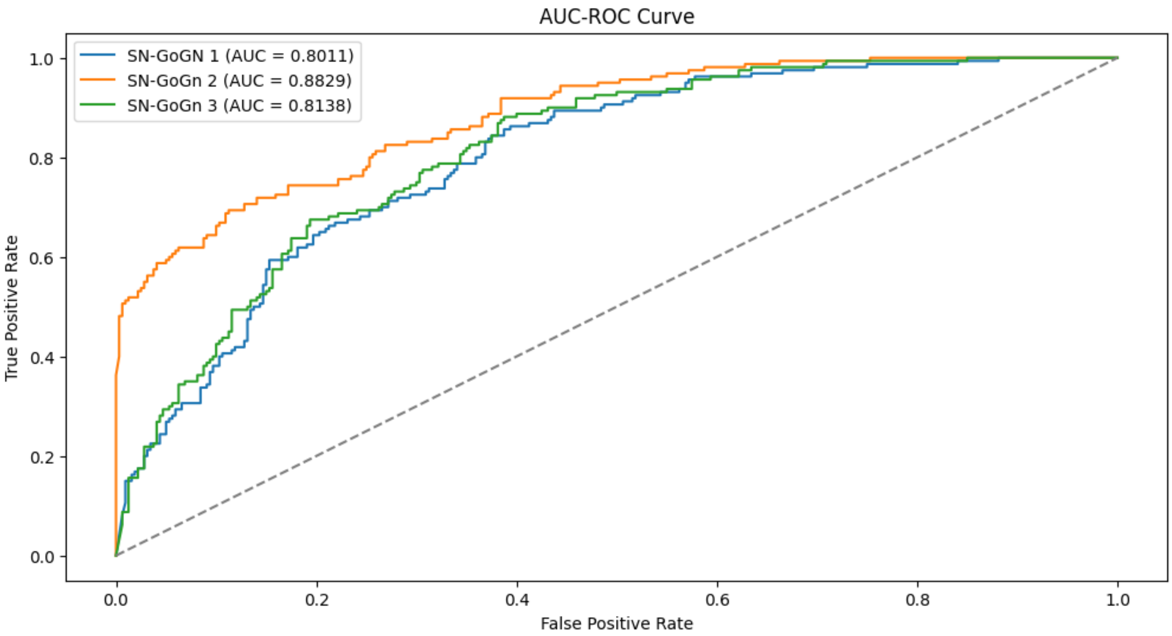

Figure S3. 4 Precision–recall curve for SN-GoGn classified by ConvNet

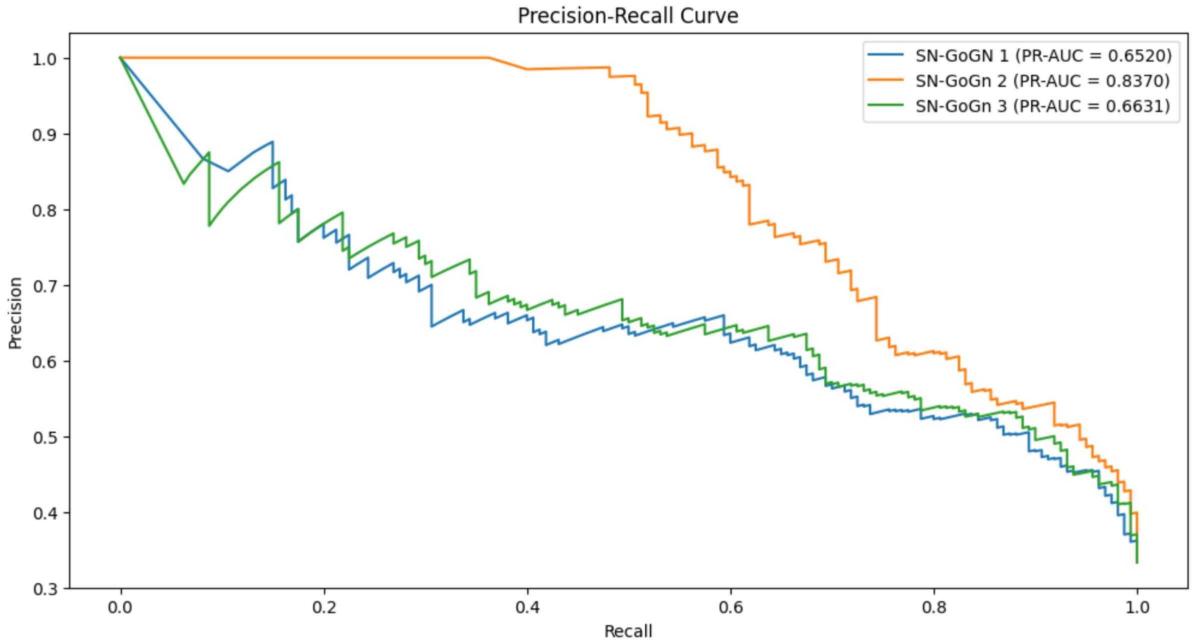

Table S3. 1 Classification Report for SN-GoGn by ConvNet

```
Mean Absolute Error (MAE): 0.5021
Cohen's Kappa: 0.4750
Classification Report:
```

	precision	recall	f1-score	support
SN-GoGN 1	0.6291	0.5938	0.6109	160
SN-GoGn 2	0.6970	0.7188	0.7077	160
SN-GoGn 3	0.6220	0.6375	0.6296	160
accuracy			0.6500	480
macro avg	0.6494	0.6500	0.6494	480
weighted avg	0.6494	0.6500	0.6494	480

Figure S3. 5 The original and Grad-CAM Images for SN-GoGn Generated by ConvNet

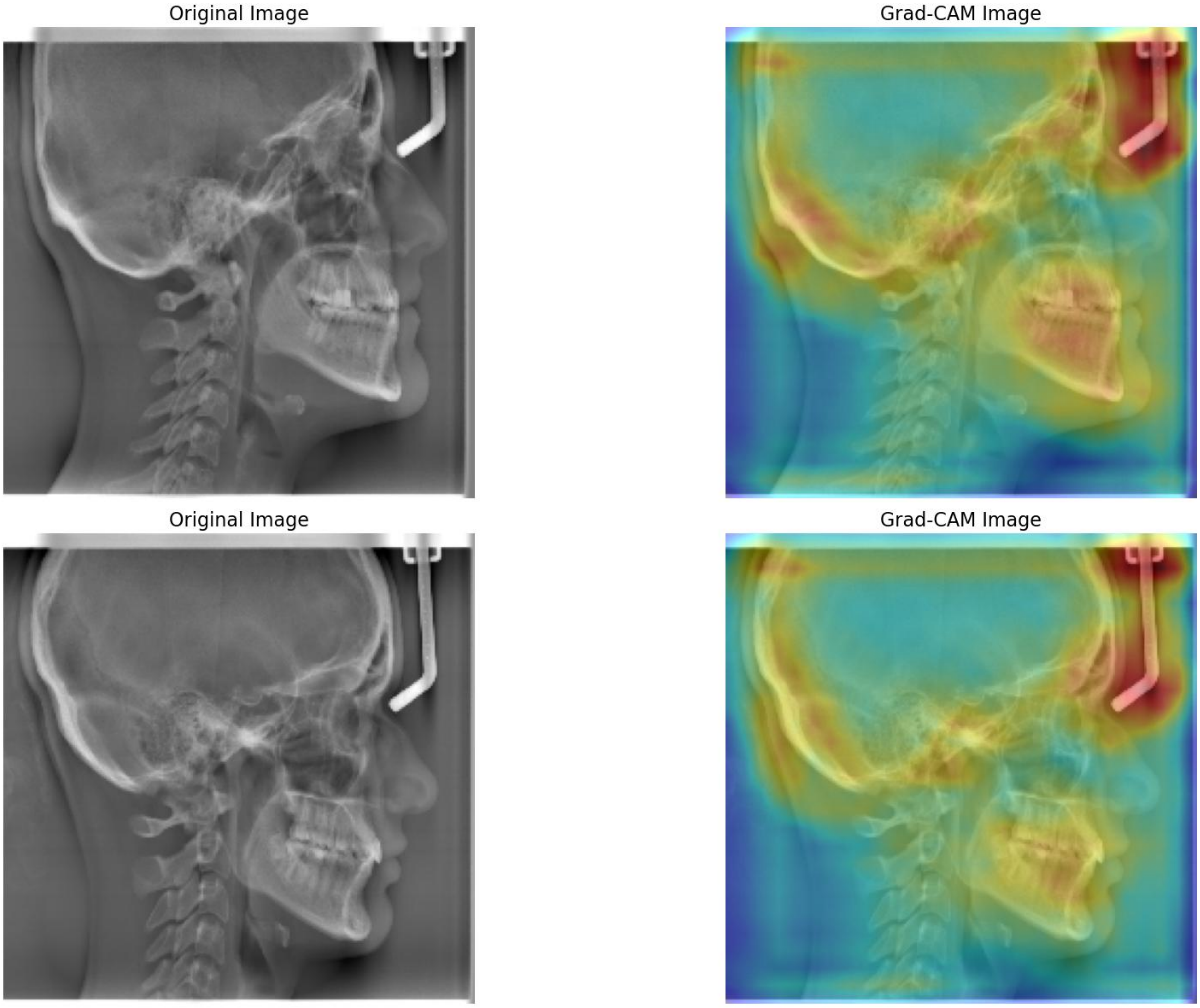

3.2 Classification of SN-GoGn by DenseNet201

Figure S3. 6 Training and Testing Loss and Training and Testing Accuracy Graphs for DenseNet201

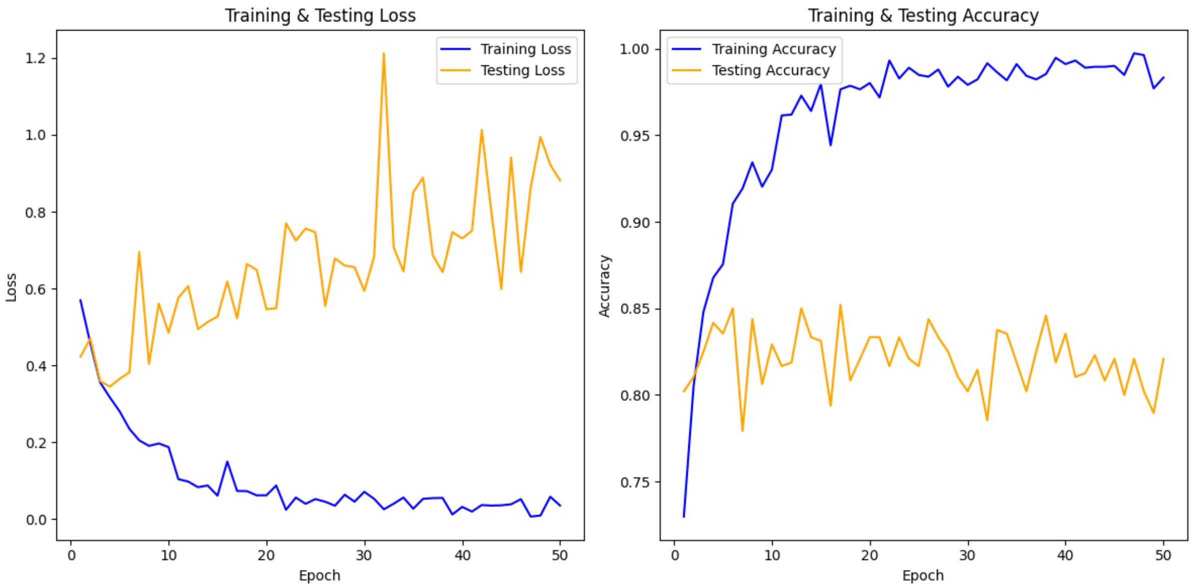

Figure S3. 7 Confusion Matrix for Actual and Predicted SN-GoGn values classified by DenseNet201

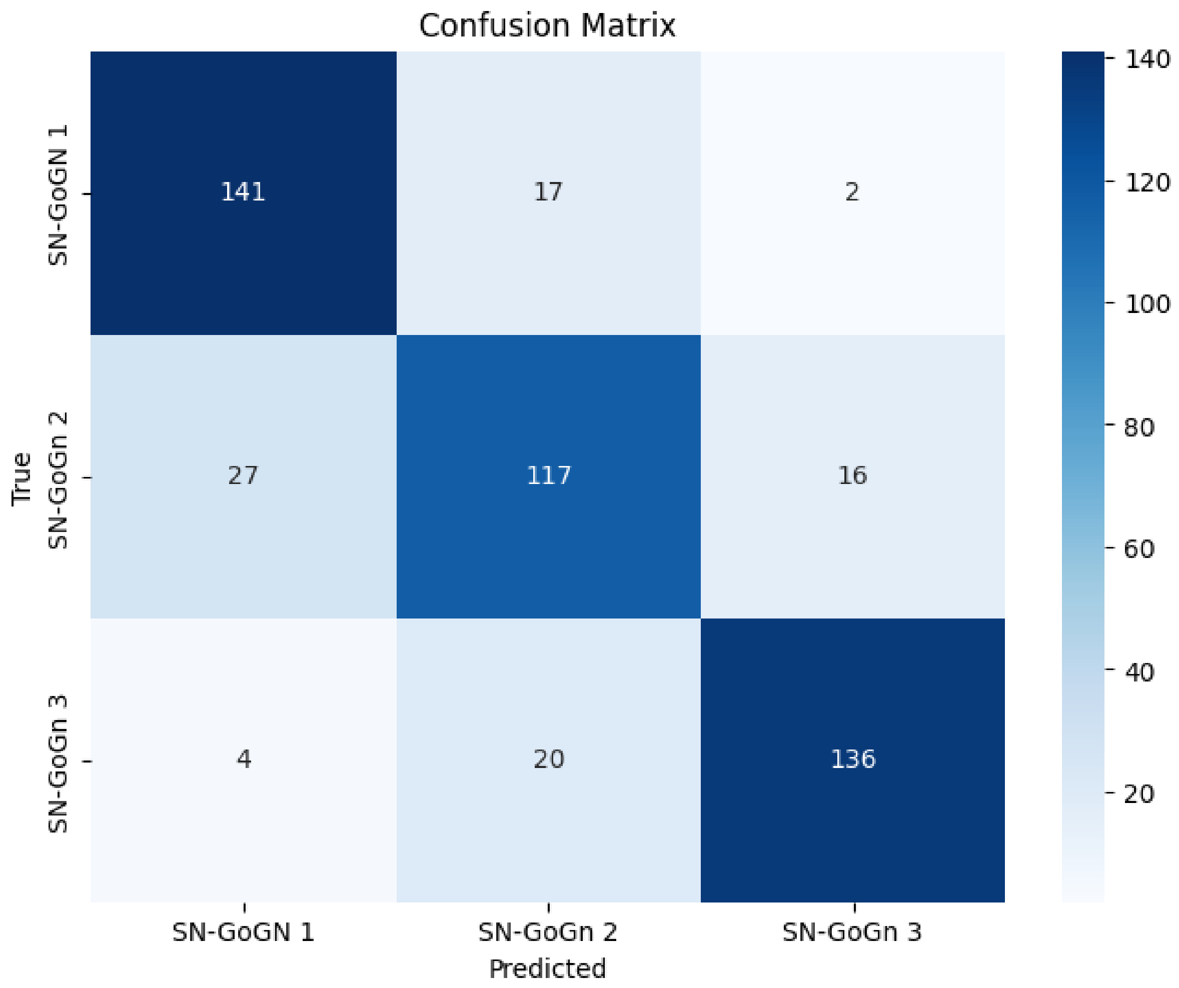

Figure S3. 8 AUC-ROC curve for SN-GoGn classified by DenseNet201

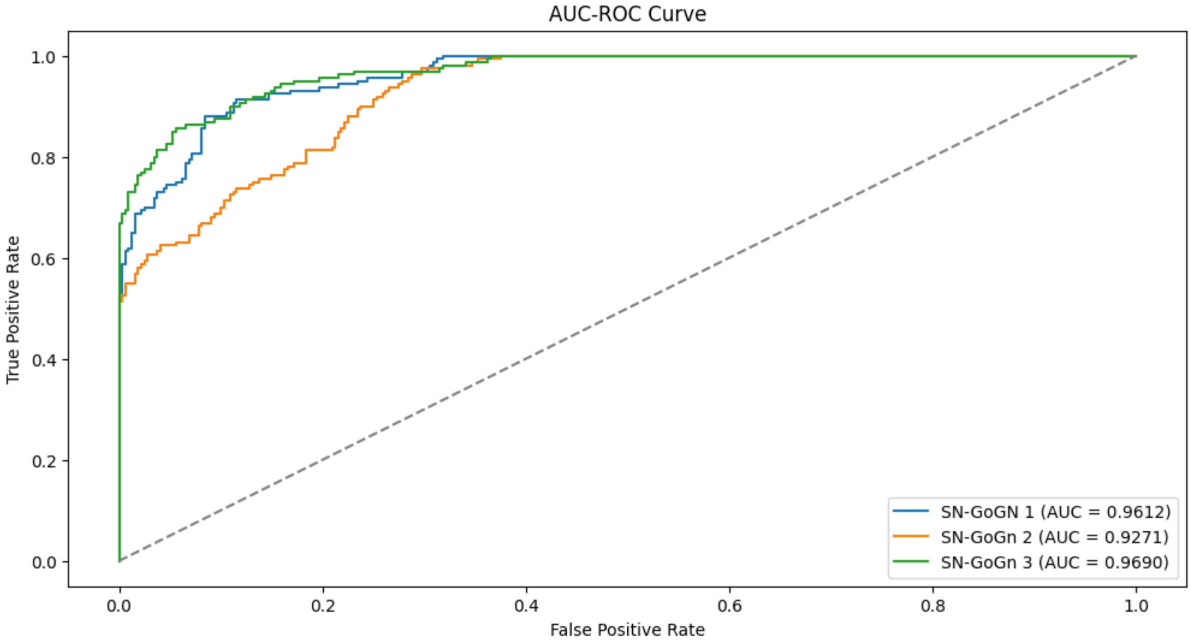

Figure S3. 9 Precision–recall curve for SN-GoGn classified by DenseNet201

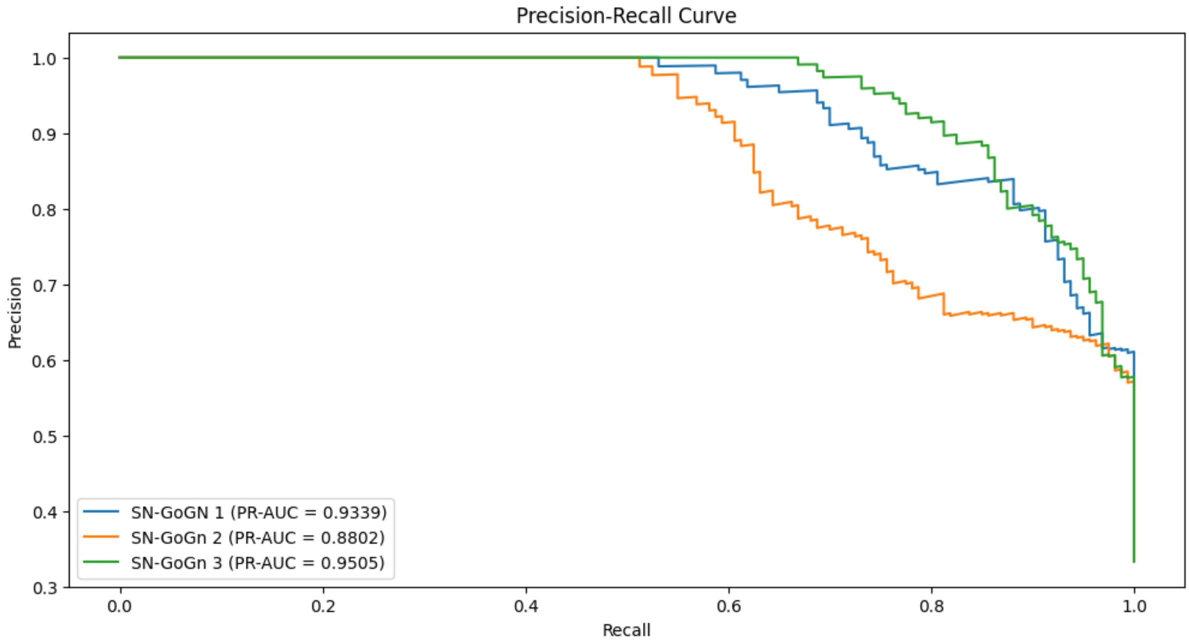

Table S3. 2 Classification Report for SN-GoGn by DenseNet201

Mean Absolute Error (MAE): 0.1917
Cohen's Kappa: 0.7312
Classification Report:

	precision	recall	f1-score	support
SN-GoGN 1	0.8198	0.8812	0.8494	160
SN-GoGn 2	0.7597	0.7312	0.7452	160
SN-GoGn 3	0.8831	0.8500	0.8662	160
accuracy			0.8208	480
macro avg	0.8209	0.8208	0.8203	480
weighted avg	0.8209	0.8208	0.8203	480

Figure S3. 10 The original and Grad-CAM Images for SN-GoGn Generated by DenseNet201

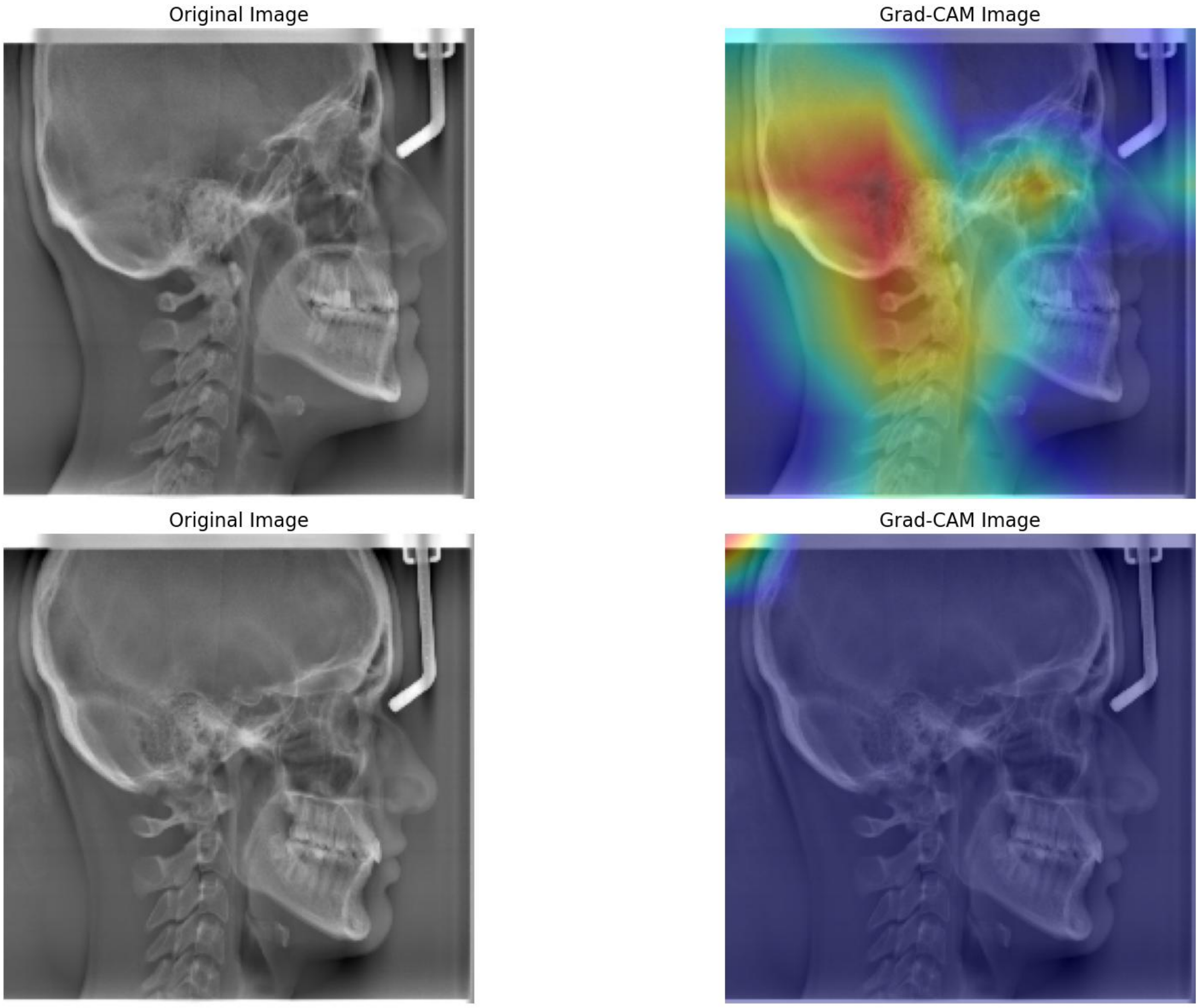

3.3 Classification of SN-GoGn by EfficientNet B0

Figure S3. 11 Training and Testing Loss and Training and Testing Accuracy Graphs for EfficientNet B0

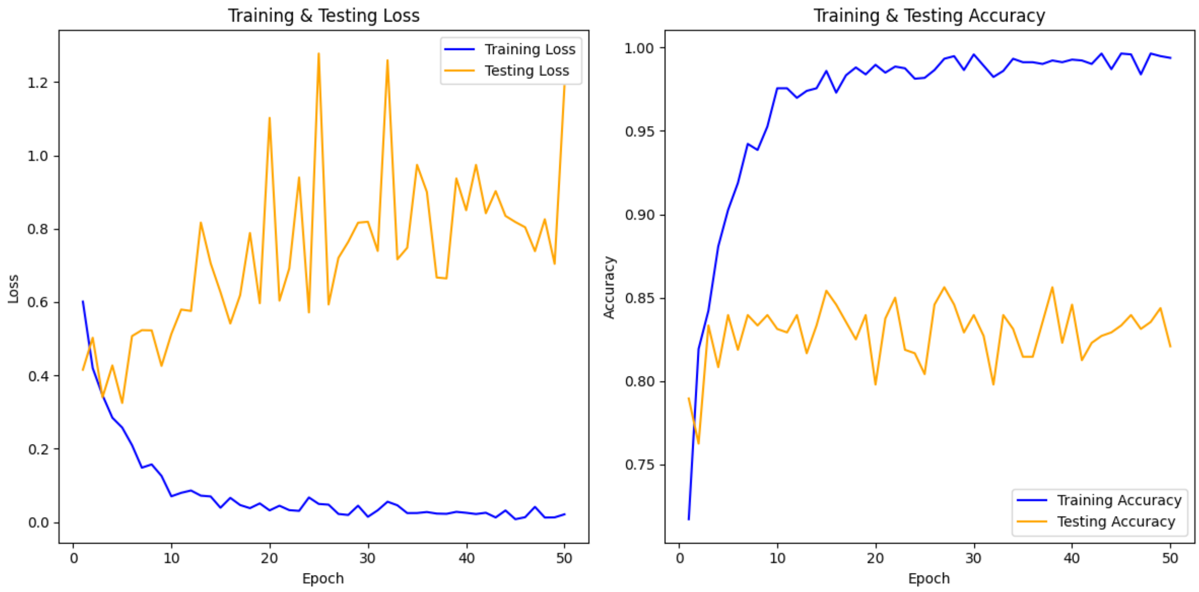

Figure S3. 12 Confusion Matrix for Actual and Predicted SN-GoGn values classified by EfficientNet B0

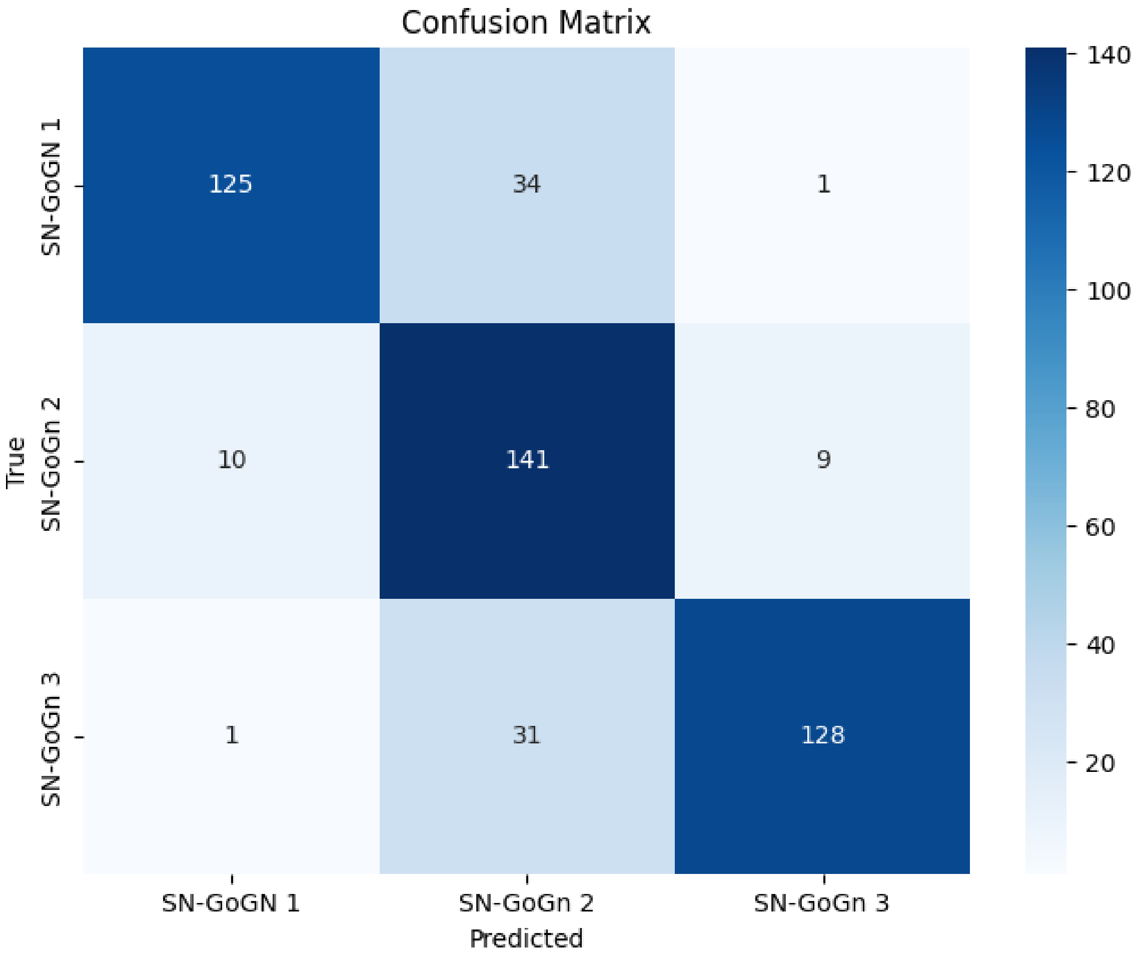

Figure S3. 13 AUC-ROC curve for SN-GoGn classified by EfficientNet B0

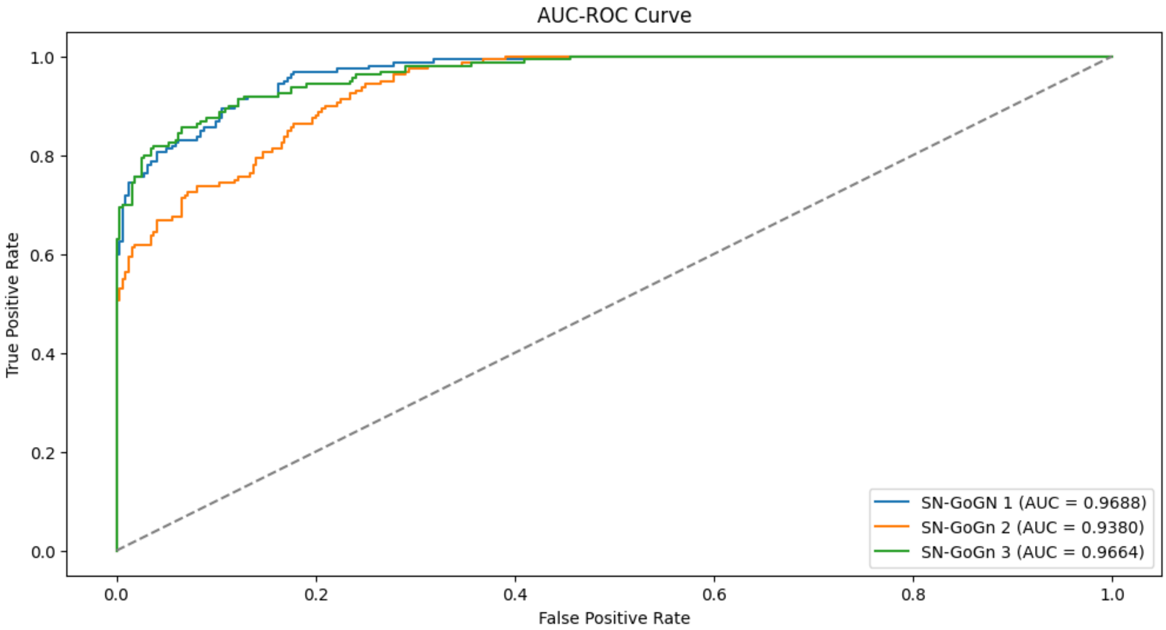

Figure S3. 14 Precision–recall curve for SN-GoGn classified by EfficientNet B0

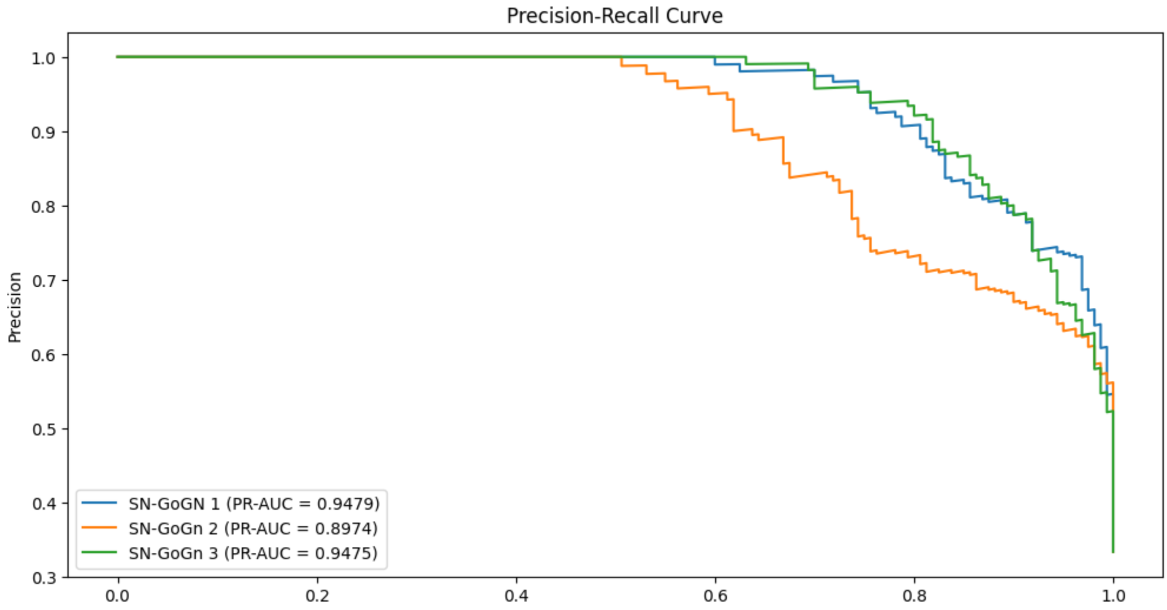

Table S3. 3 Classification Report for SN-GoGn by EfficientNet B0

Mean Absolute Error (MAE): 0.1833
Cohen's Kappa: 0.7312
Classification Report:

	precision	recall	f1-score	support
SN-GoGN 1	0.9191	0.7812	0.8446	160
SN-GoGn 2	0.6845	0.8812	0.7705	160
SN-GoGn 3	0.9275	0.8000	0.8591	160
accuracy			0.8208	480
macro avg	0.8437	0.8208	0.8247	480
weighted avg	0.8437	0.8208	0.8247	480

Figure S3. 15 The original and Grad-CAM Images for SN-GoGn Generated by EfficientNet B0

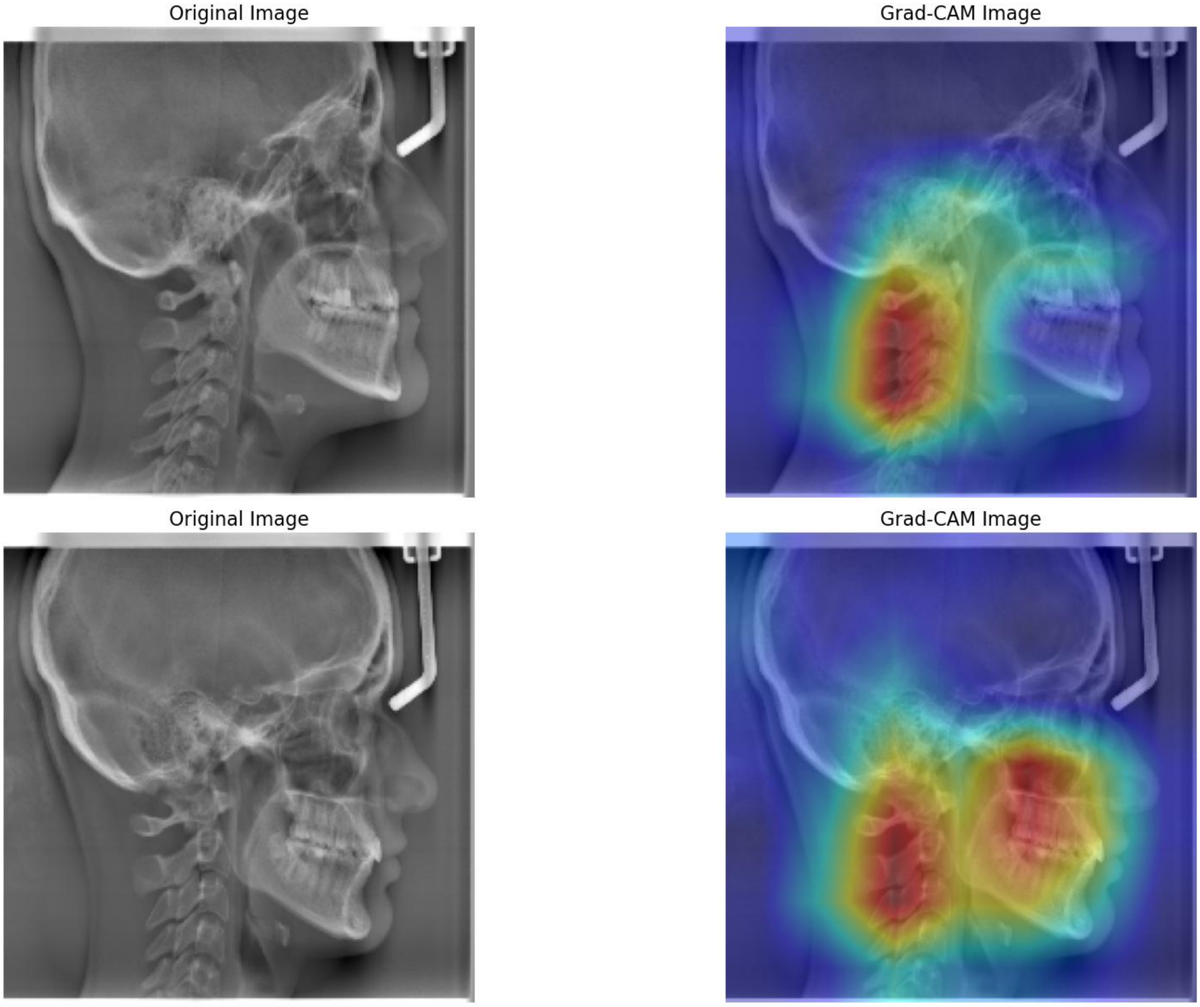

3.4 Classification of SN-GoGn by EfficientNet V2

Figure S3. 16 Training and Testing Loss and Training and Testing Accuracy Graphs for EfficientNet V2

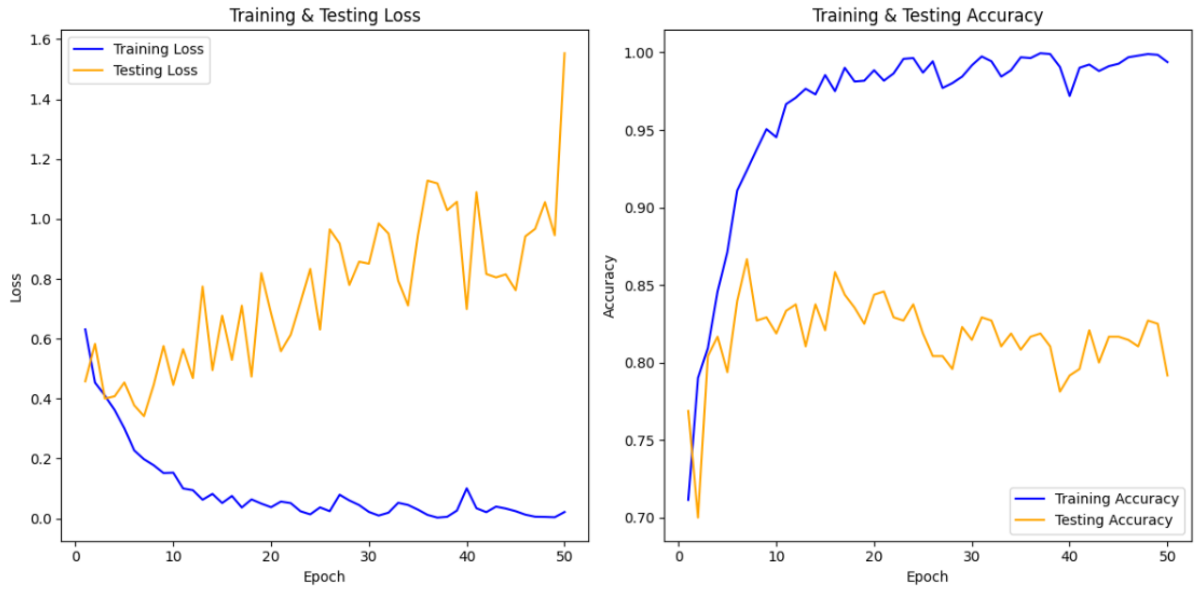

Figure S3. 17 Confusion Matrix for Actual and Predicted SN-GoGn values classified by EfficientNet V2

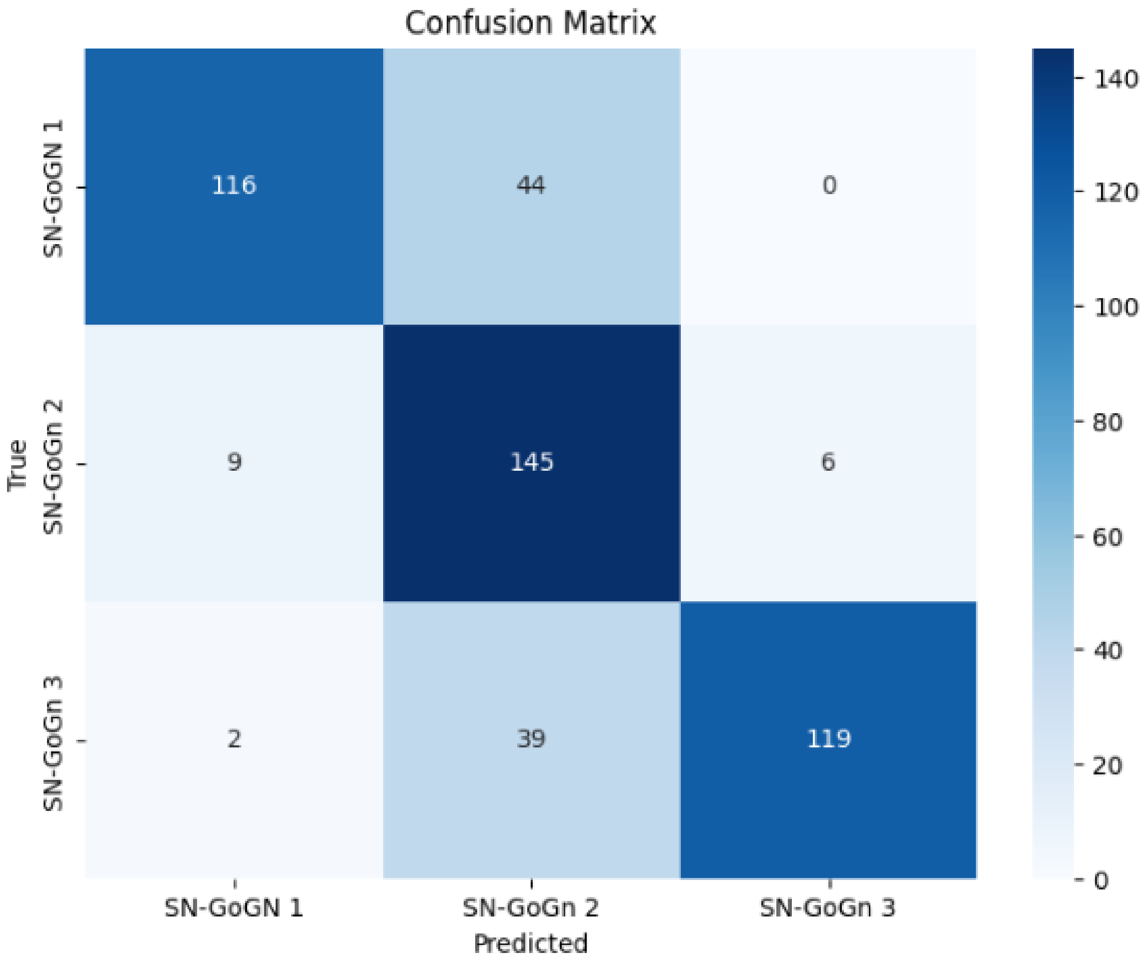

Figure S3. 18 AUC-ROC curve for SN-GoGn classified by EfficientNet V2

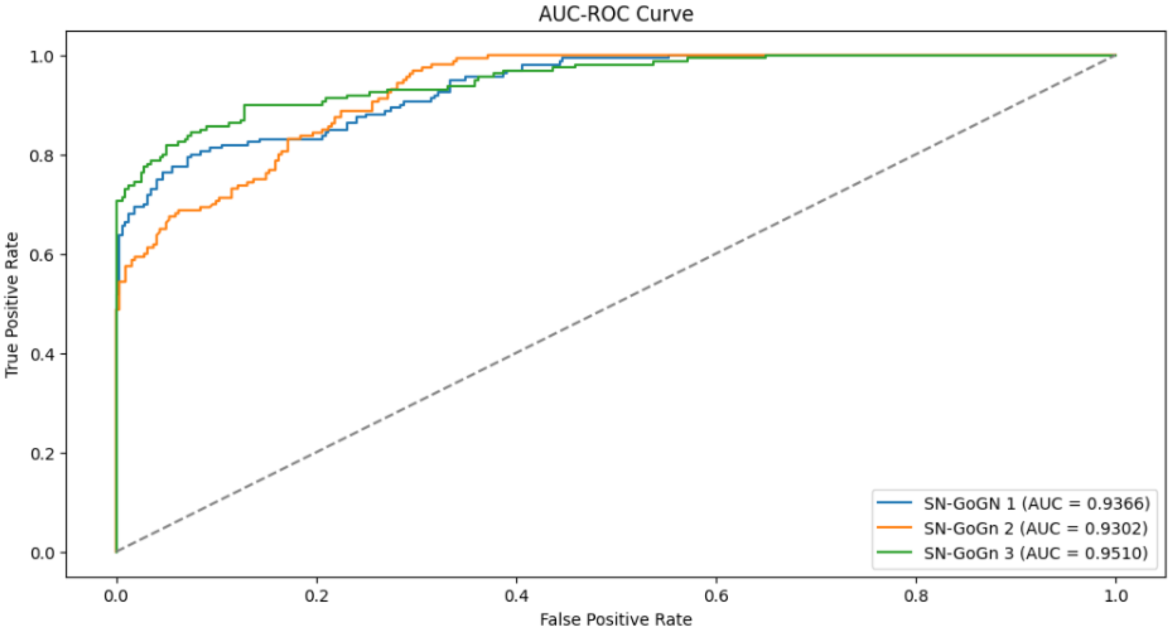

Figure S3. 19 Precision–recall curve for SN-GoGn classified by EfficientNet V2

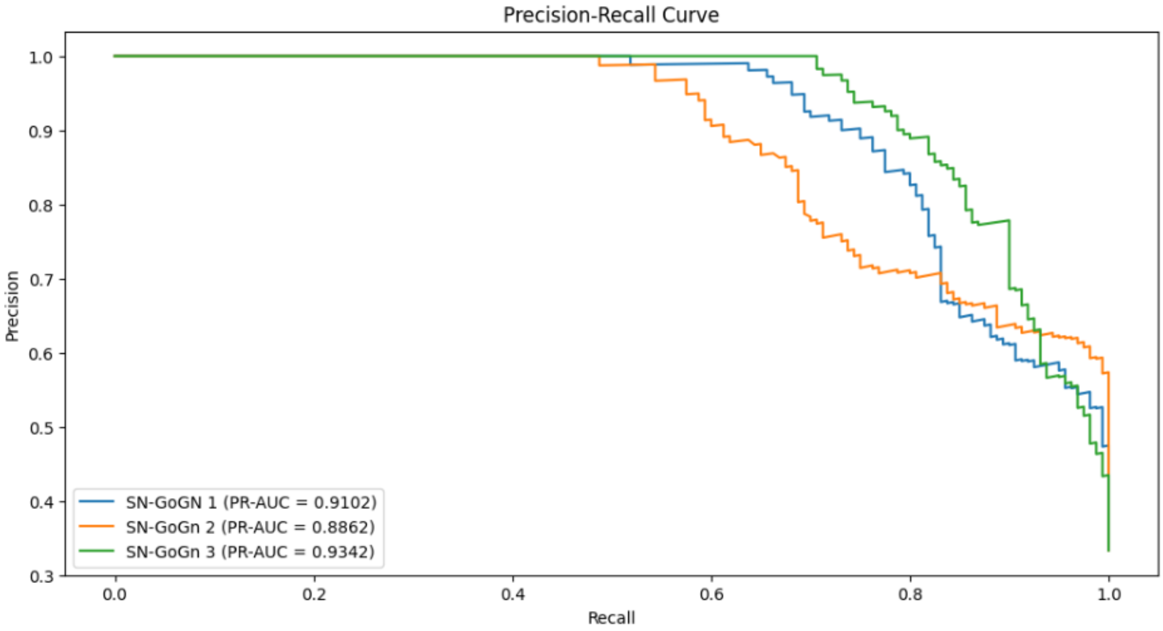

Table S3. 4 Classification Report for SN-GoGn by EfficientNet V2

Mean Absolute Error (MAE): 0.2125
Cohen's Kappa: 0.6875
Classification Report:

	precision	recall	f1-score	support
SN-GoGN 1	0.9134	0.7250	0.8084	160
SN-GoGn 2	0.6360	0.9062	0.7474	160
SN-GoGn 3	0.9520	0.7438	0.8351	160
accuracy			0.7917	480
macro avg	0.8338	0.7917	0.7970	480
weighted avg	0.8338	0.7917	0.7970	480

Figure S3. 20 The original and Grad-CAM Images for SN-GoGn Generated by EfficientNet V2

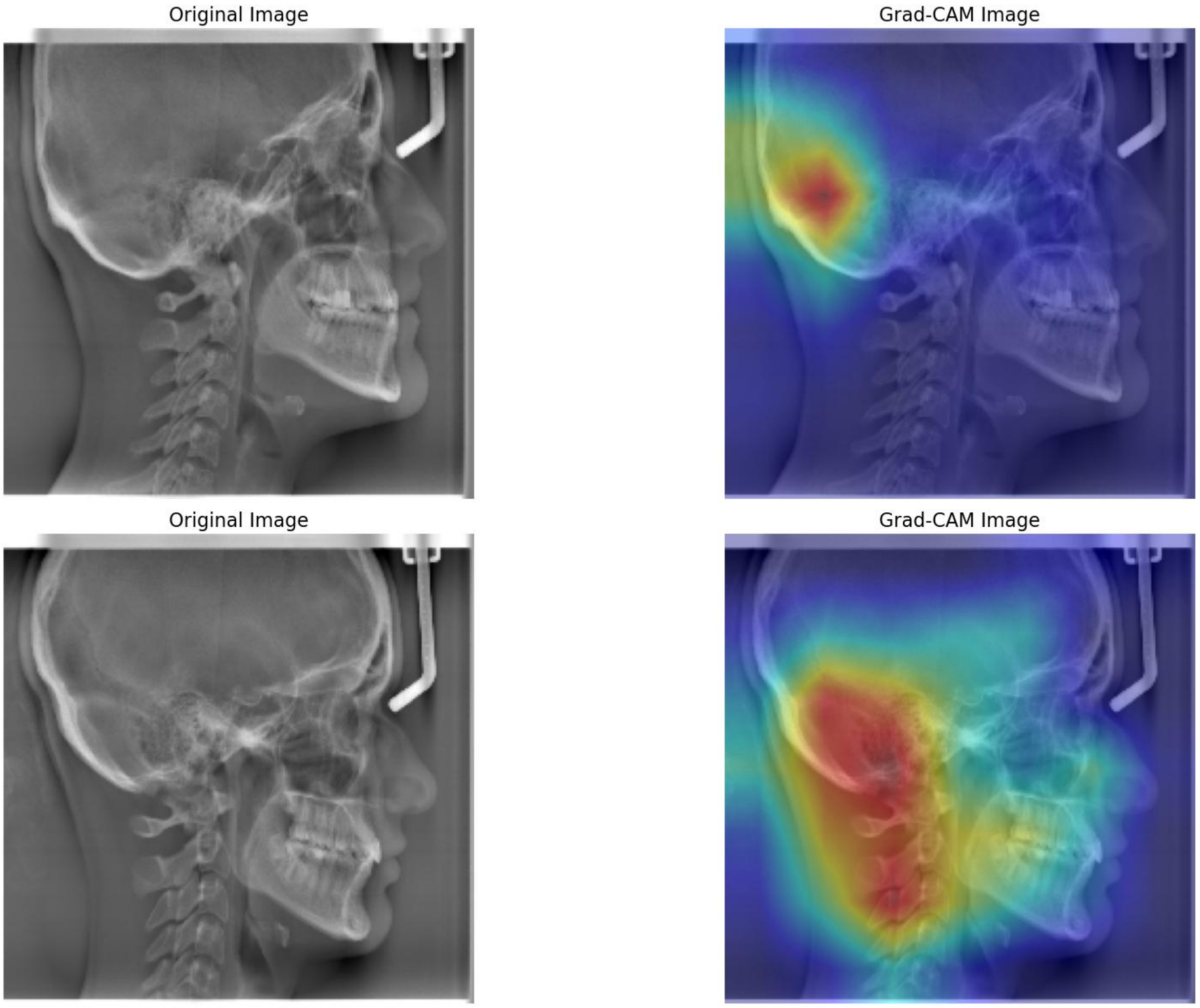

3.5 Classification of SN-GoGn by Hybrid Algorithm

Figure S3. 21 Training and Testing Loss and Training and Testing Accuracy Graphs for Hybrid Algorithm

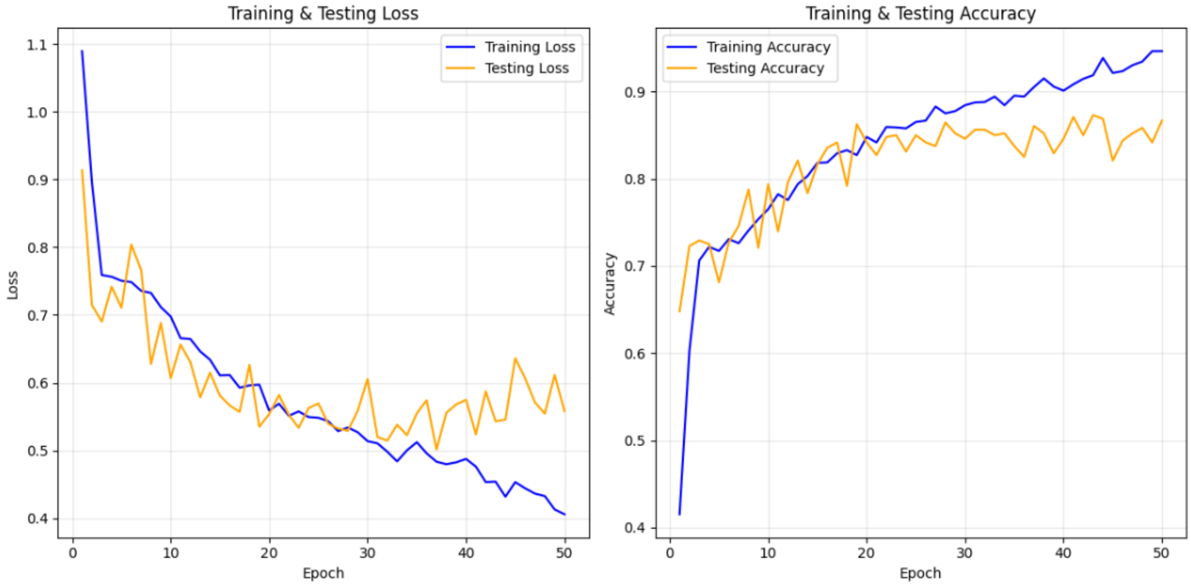

Figure S3. 22 Confusion Matrix for Actual and Predicted SN-GoGn values classified by Hybrid Algorithm

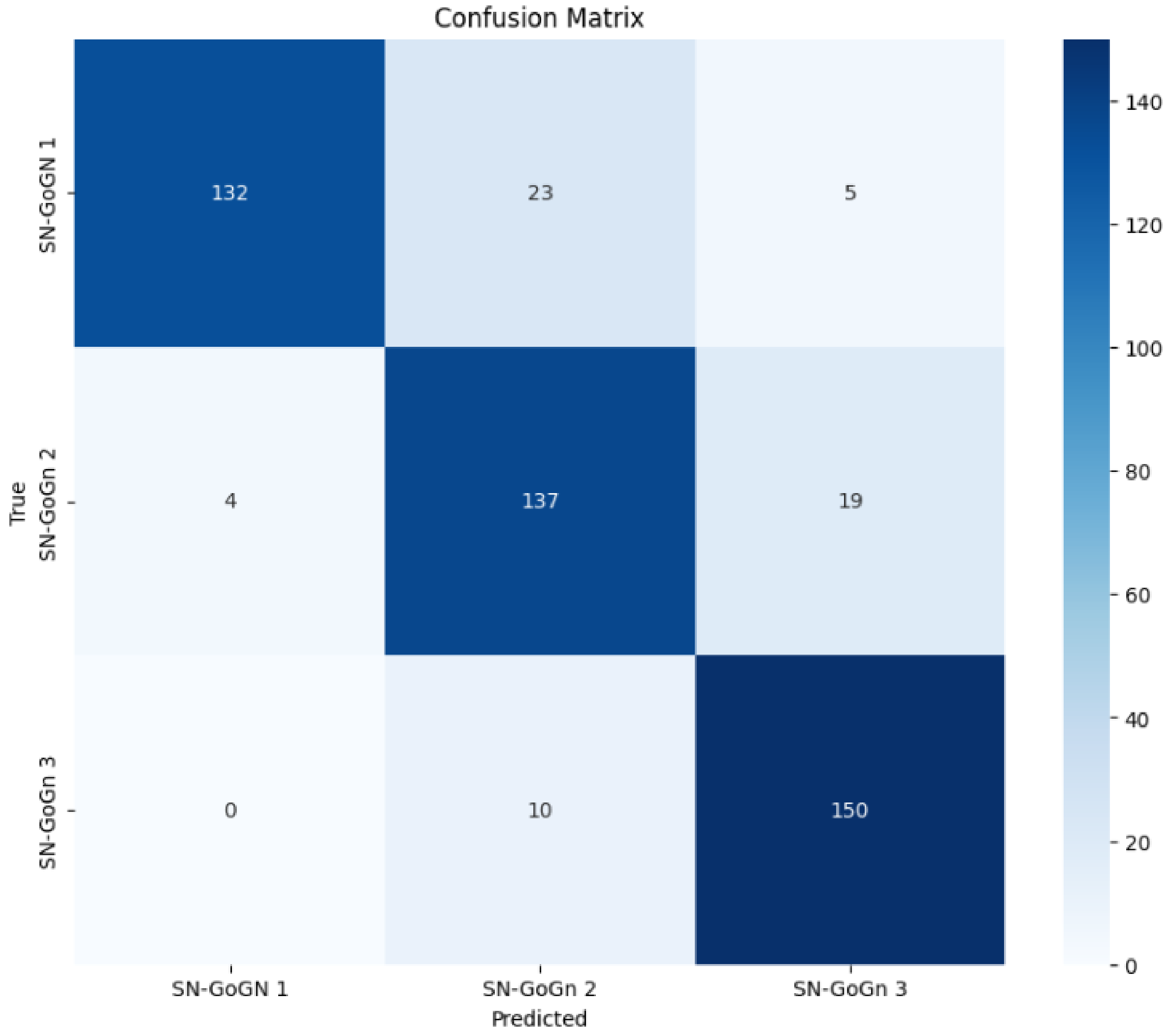

Figure S3. 23 AUC-ROC curve for SN-GoGn classified by Hybrid Algorithm

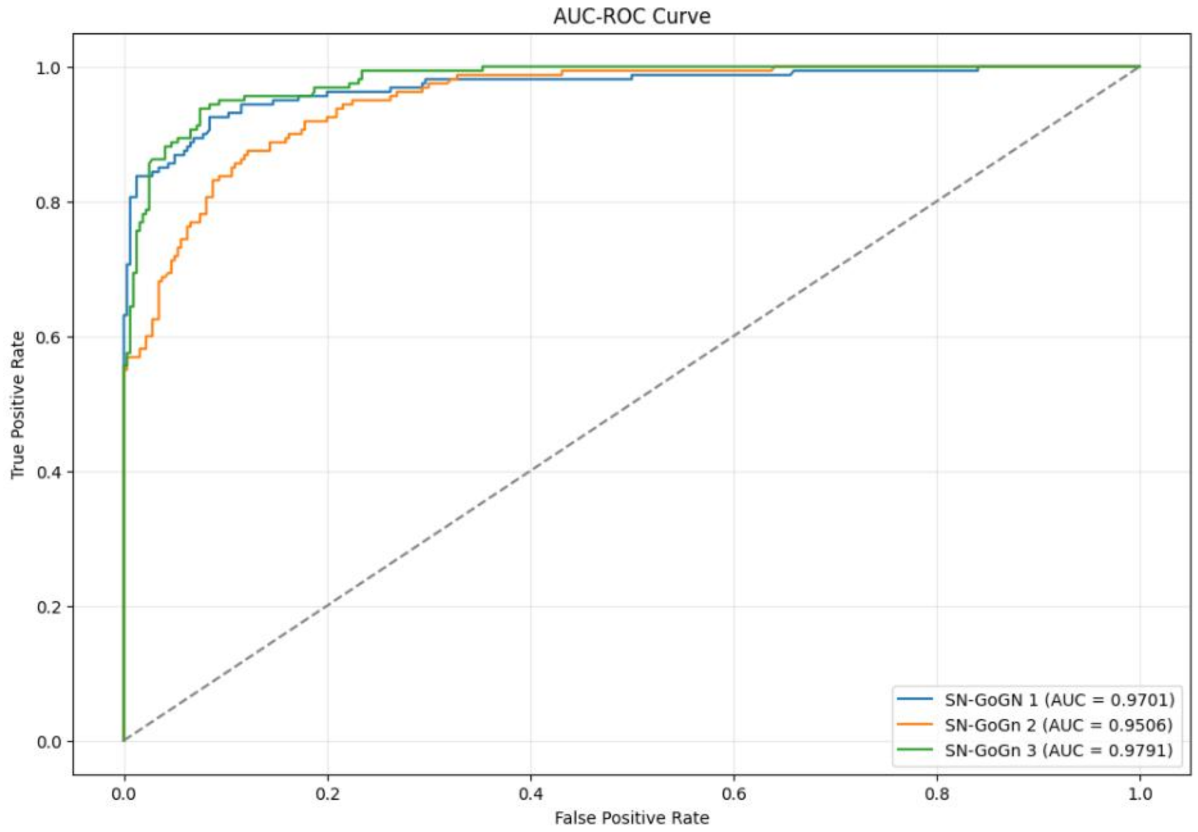

Figure S3. 24 Precision–recall curve for SN-GoGn classified by Hybrid Algorithm

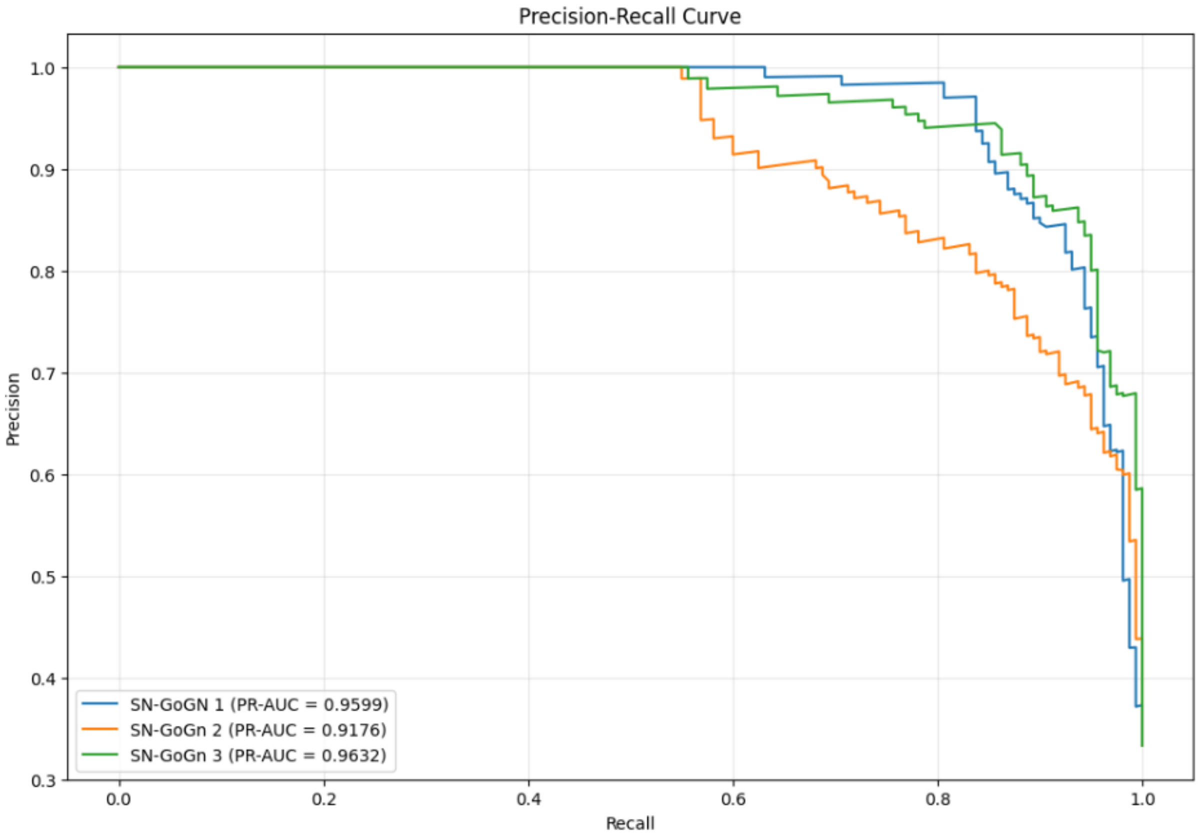

Table S3. 5 Classification Report for SN-GoGn by Hybrid Algorithm

Mean Absolute Error (MAE): 0.1375
Cohen's Kappa: 0.8094
Classification Report:

	precision	recall	f1-score	support
SN-GoGN 1	0.9706	0.8250	0.8919	160
SN-GoGn 2	0.8059	0.8562	0.8303	160
SN-GoGn 3	0.8621	0.9375	0.8982	160
accuracy			0.8729	480
macro avg	0.8795	0.8729	0.8735	480
weighted avg	0.8795	0.8729	0.8735	480

Figure S3. 25 The original and Grad-CAM Images for SN-GoGn Generated by Hybrid Algorithm

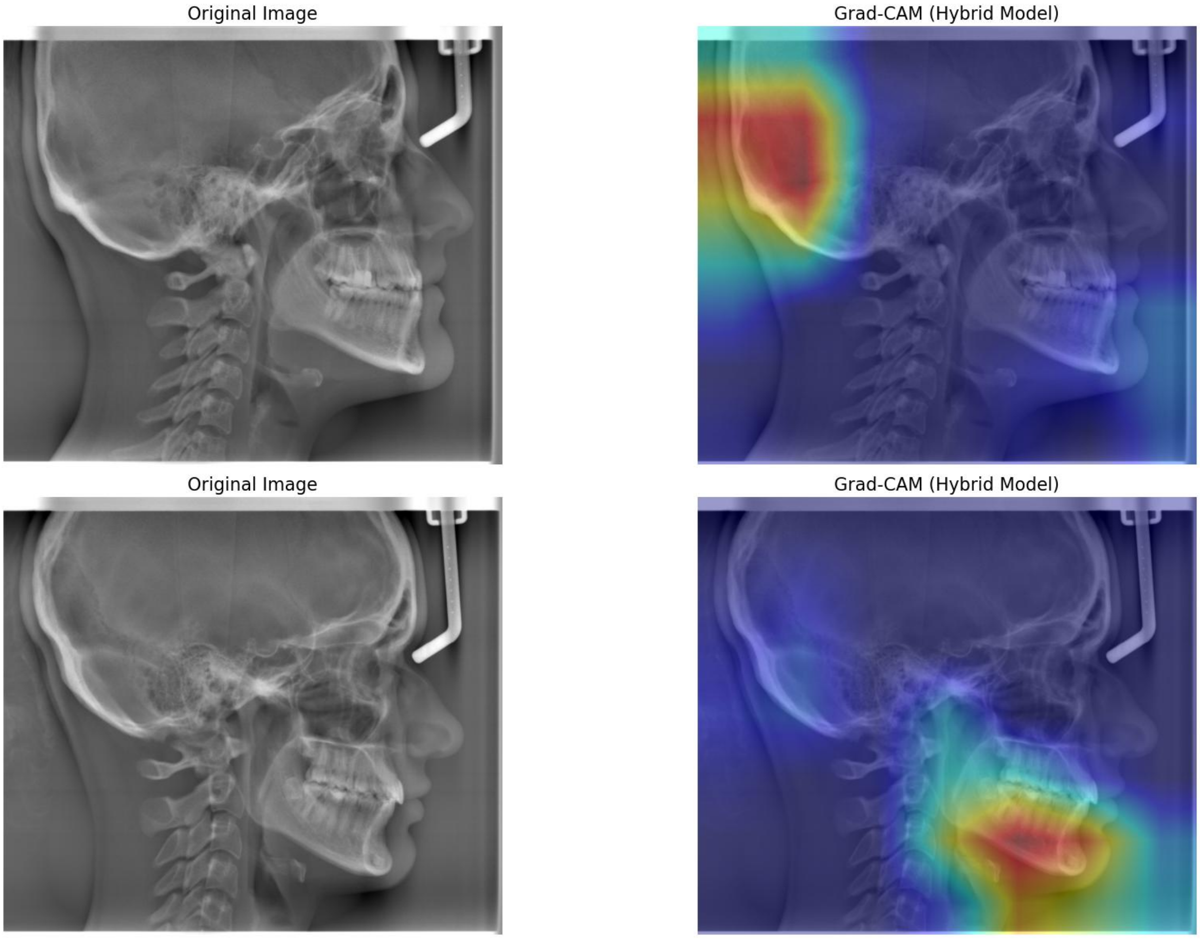

3.6 Classification of SN-GoGn by MobileNetV2

Figure S3. 26 Training and Testing Loss and Training and Testing Accuracy Graphs for MobileNetV2

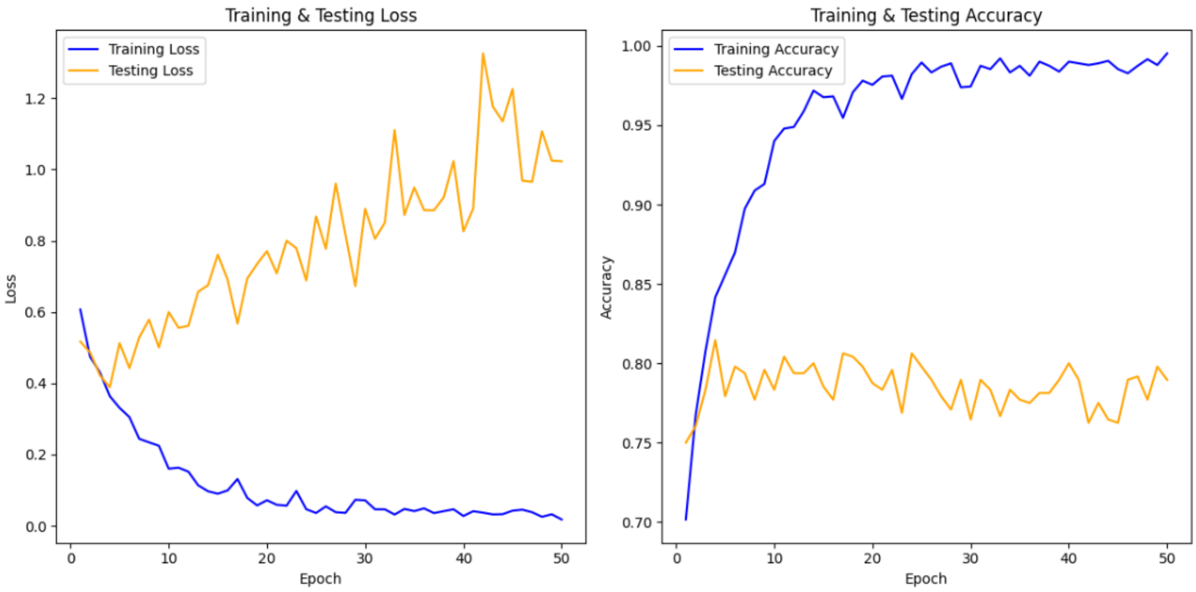

Figure S3. 27 Confusion Matrix for Actual and Predicted SN-GoGn values classified by MobileNetV2

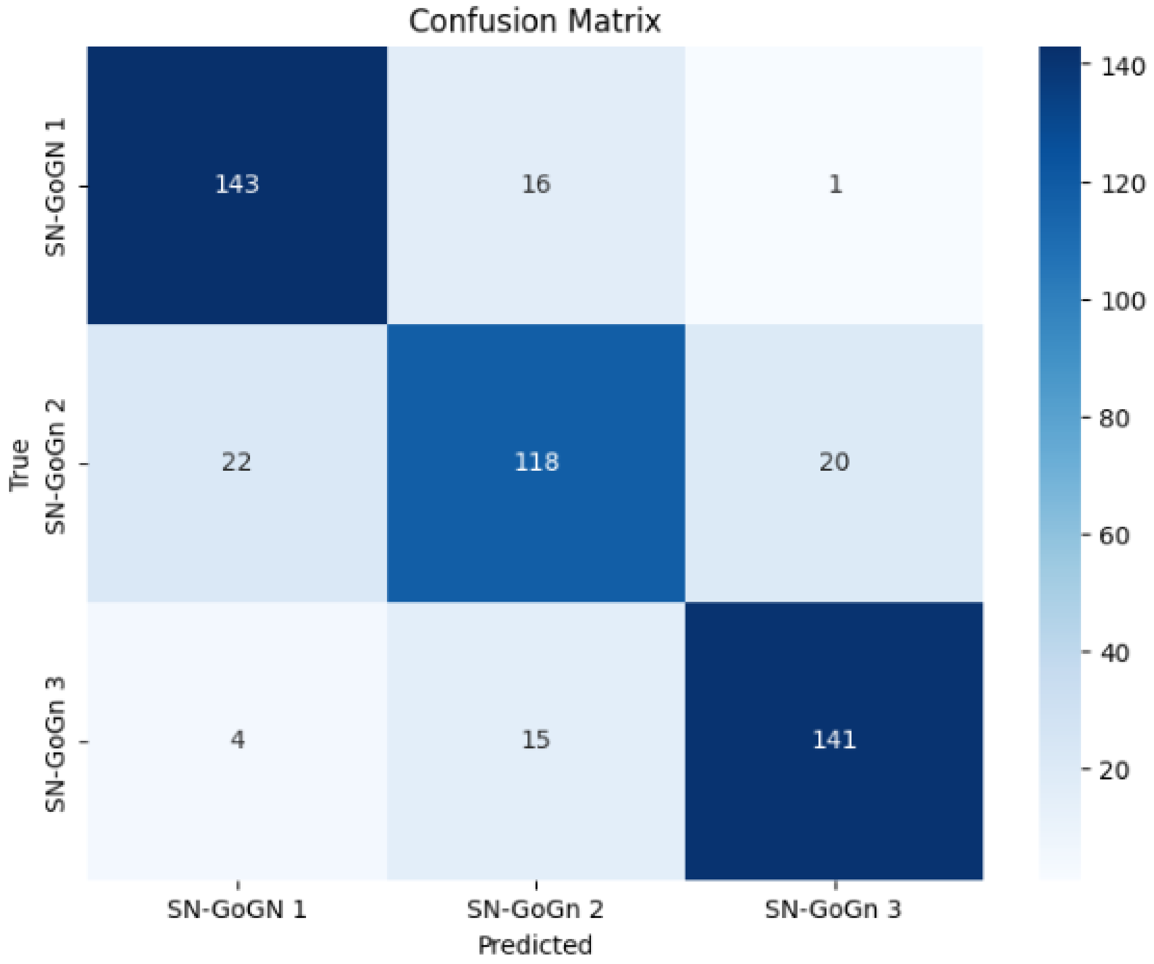

Figure S3. 28 AUC-ROC curve for SN-GoGn classified by MobileNetV2

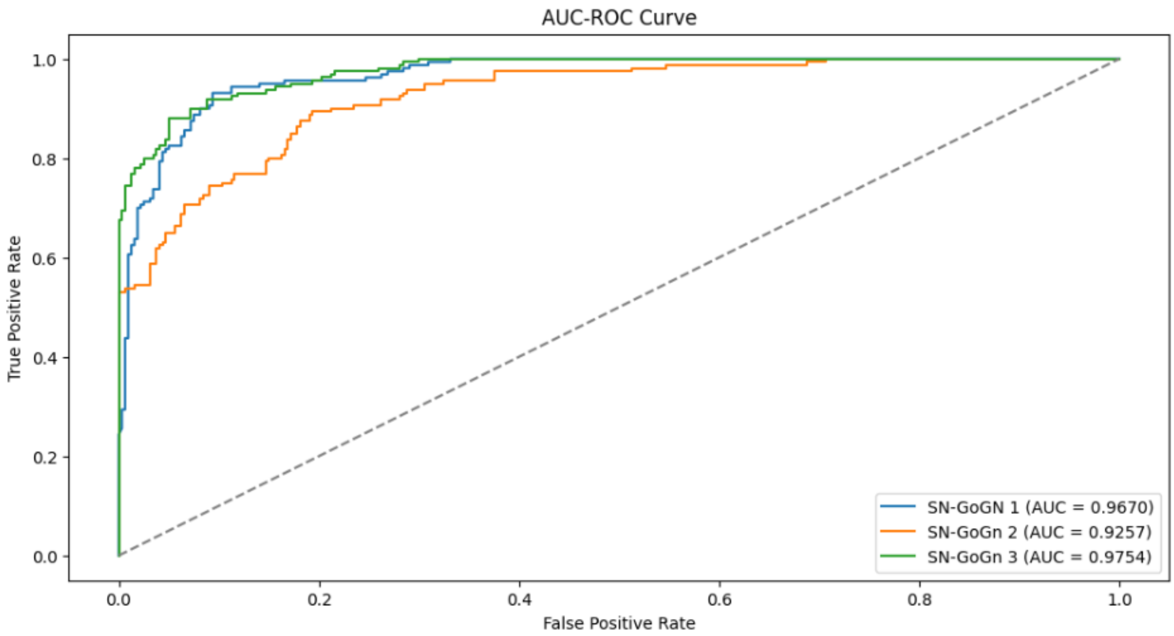

Figure S3. 29 Precision–recall curve for SN-GoGn classified by MobileNetV2

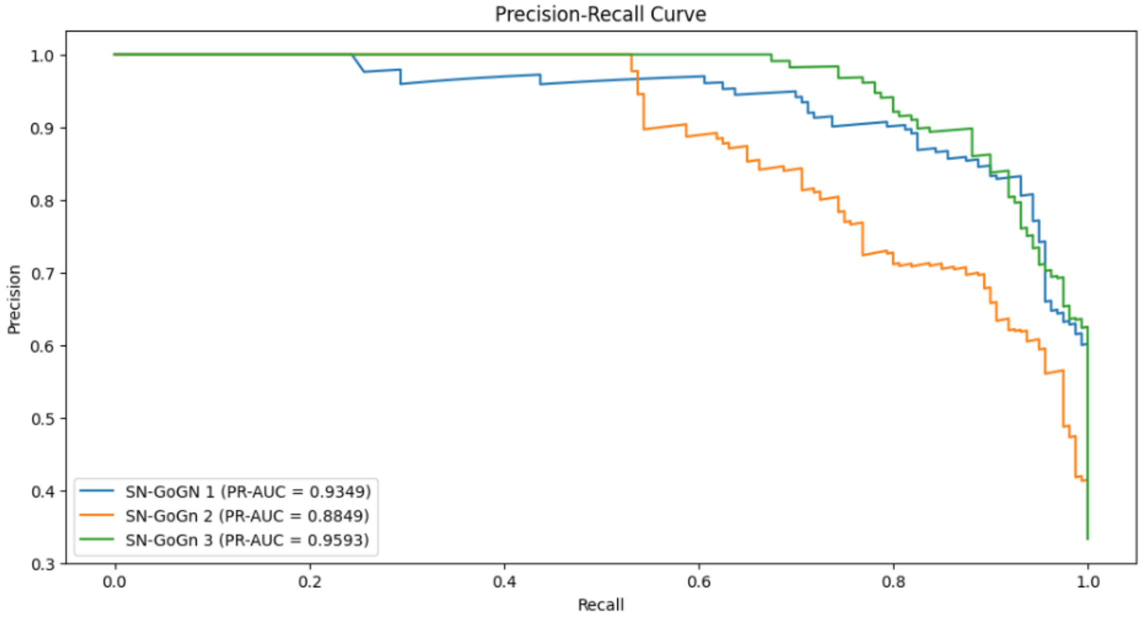

Table S3. 6 Classification Report for SN-GoGn by MobileNetV2

Mean Absolute Error (MAE): 0.1729
Cohen's Kappa: 0.7562
Classification Report:

	precision	recall	f1-score	support
SN-GoGN 1	0.8462	0.8938	0.8693	160
SN-GoGn 2	0.7919	0.7375	0.7638	160
SN-GoGn 3	0.8704	0.8812	0.8758	160
accuracy			0.8375	480
macro avg	0.8362	0.8375	0.8363	480
weighted avg	0.8362	0.8375	0.8363	480

Figure S1. 30 The original and Grad-CAM Images for Cant of SN-GoGn Generated by MobileNetV2

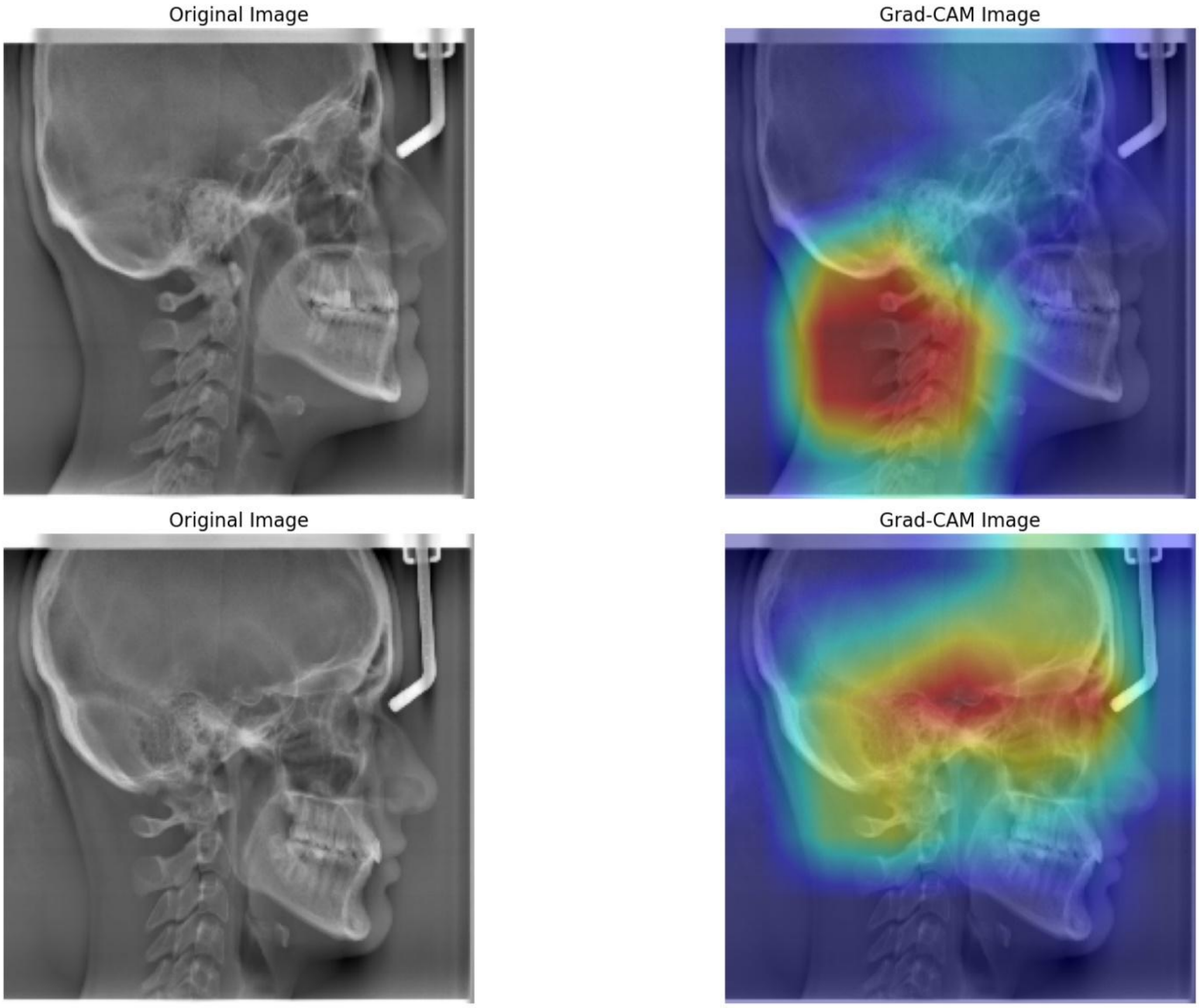

3.7 Classification of SN-GoGn by ResNet101

Figure S3. 31 Training and Testing Loss and Training and Testing Accuracy Graphs for ResNet101

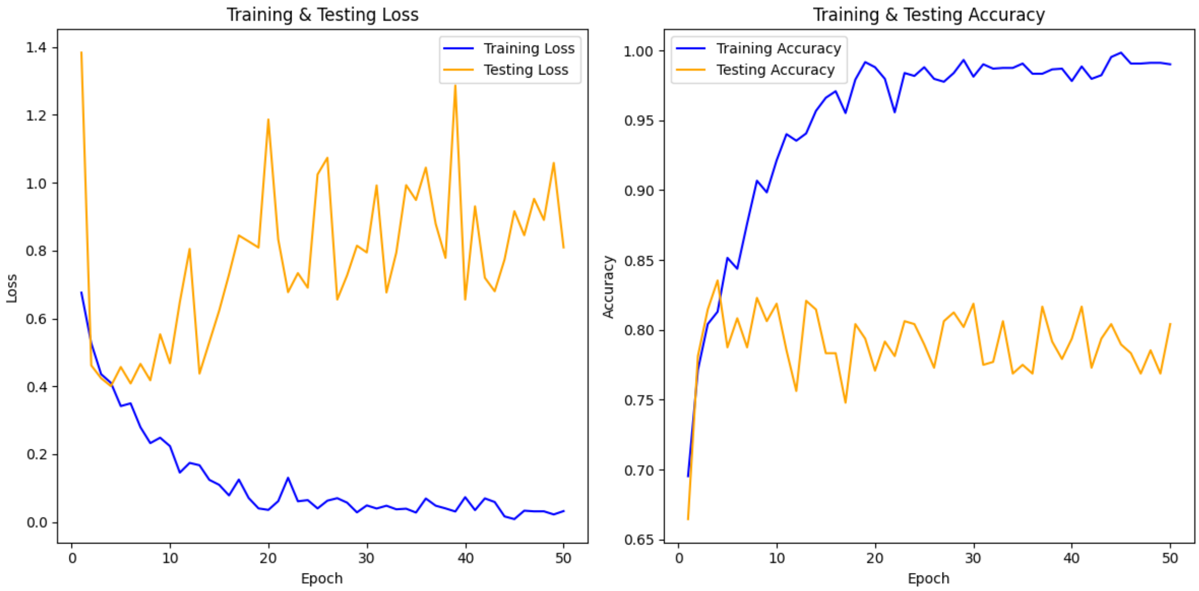

Figure S3. 32 Confusion Matrix for Actual and Predicted SN-GoGn values classified by ResNet101

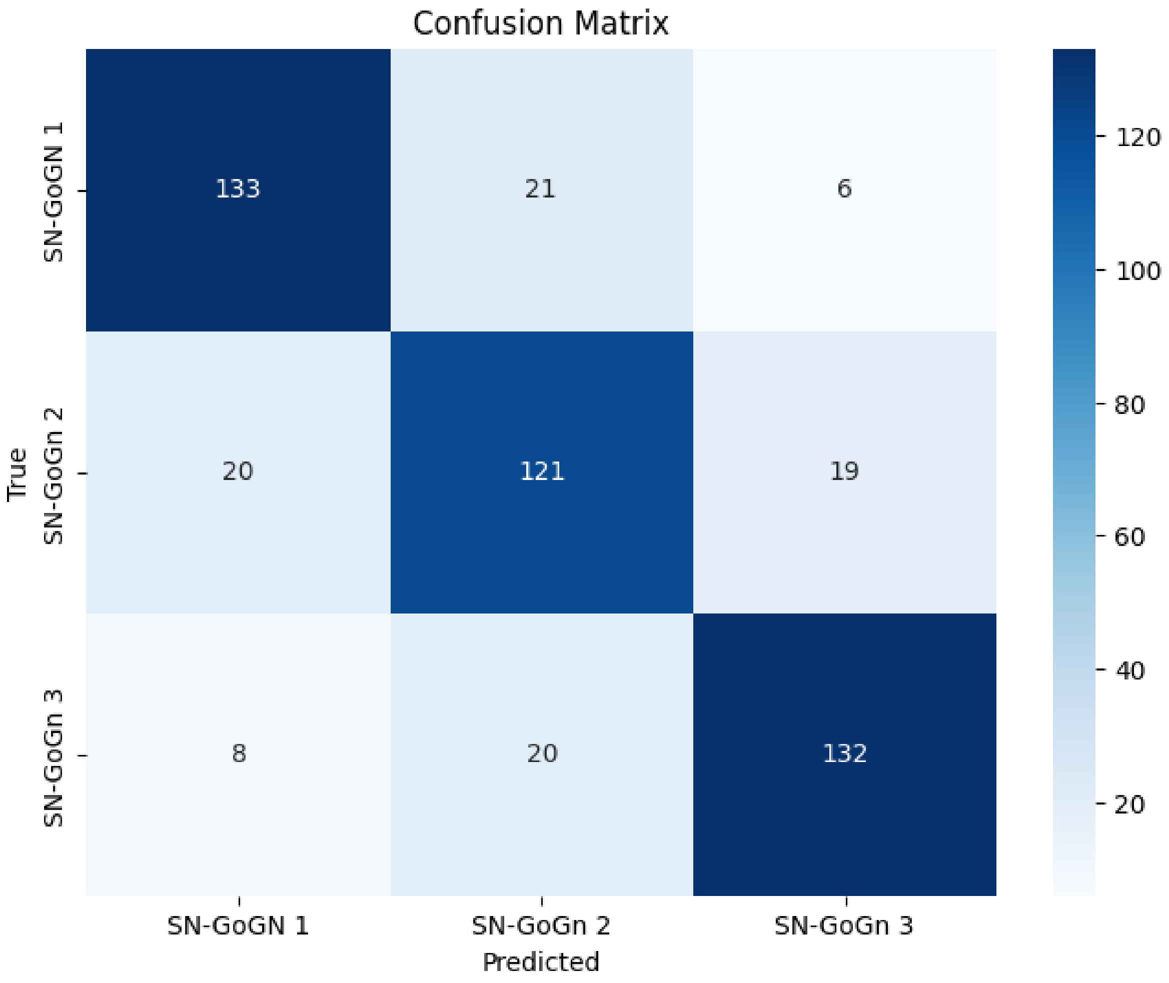

Figure S3. 33 AUC-ROC curve for SN-GoGn classified by ResNet101

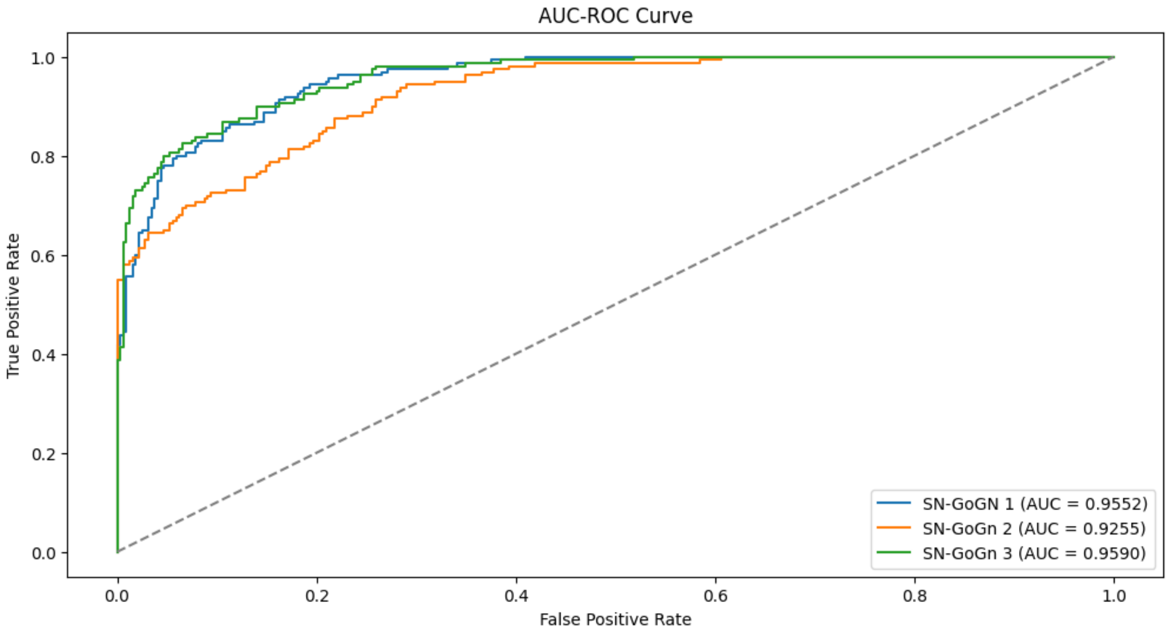

Figure S3. 34 Precision–recall curve for SN-GoGn classified by ResNet101

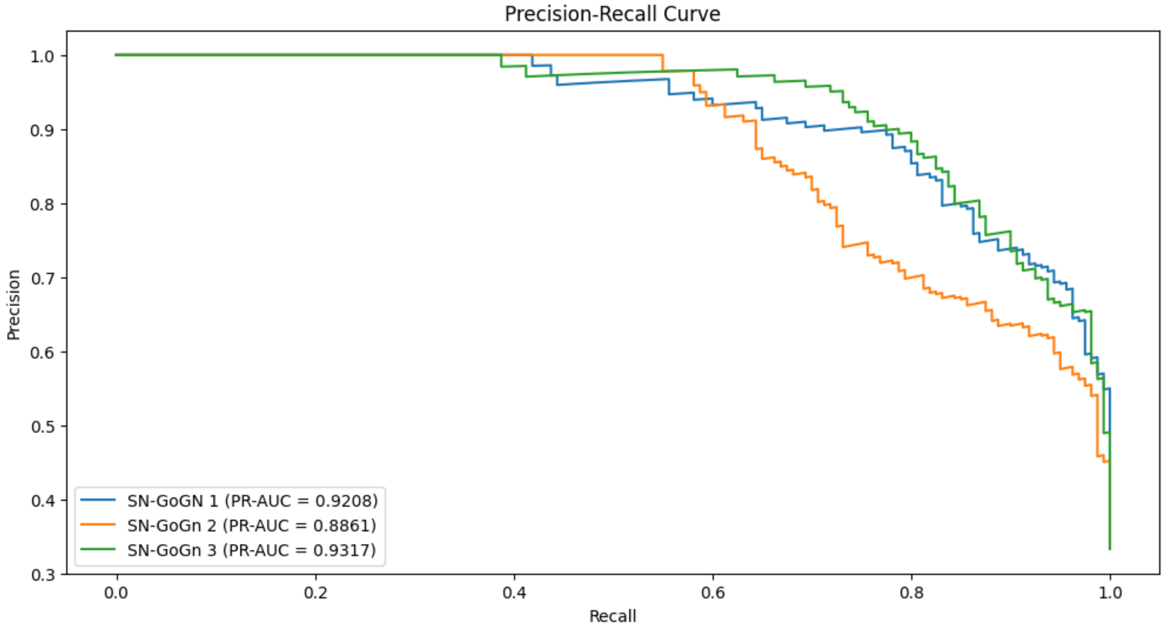

Table S3. 7 Classification Report for SN-GoGn by ResNet101

Mean Absolute Error (MAE): 0.2250
Cohen's Kappa: 0.7063
Classification Report:

	precision	recall	f1-score	support
SN-GoGN 1	0.8261	0.8313	0.8287	160
SN-GoGn 2	0.7469	0.7562	0.7516	160
SN-GoGn 3	0.8408	0.8250	0.8328	160
accuracy			0.8042	480
macro avg	0.8046	0.8042	0.8043	480
weighted avg	0.8046	0.8042	0.8043	480

Figure S3. 35 The original and Grad-CAM Images for SN-GoGn Generated by ResNet101

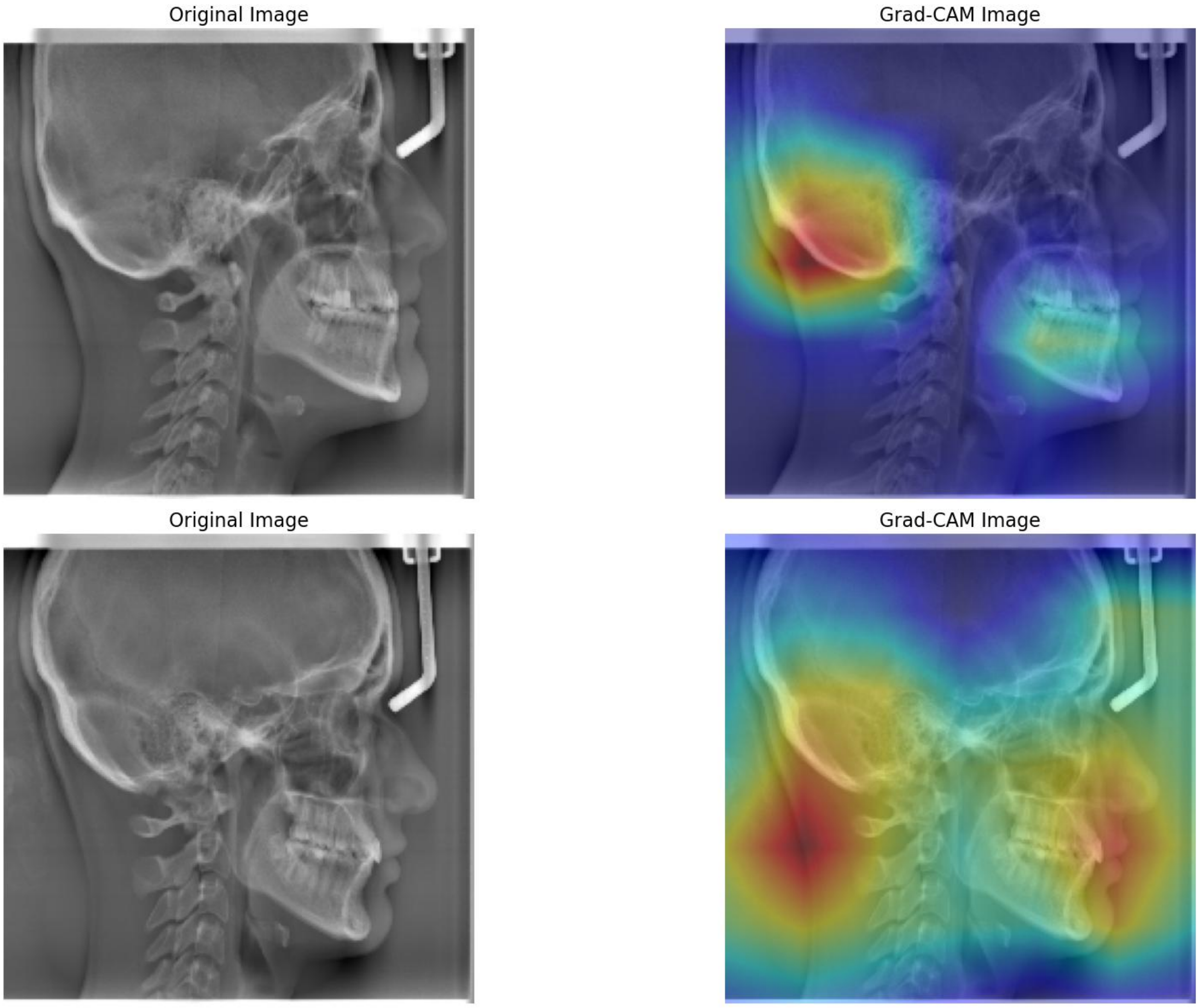

Figure S3. 36 Comparisons of Models According to Accuracy , Mean Absolute Error and Cohen's Kappa for SN-GoGn

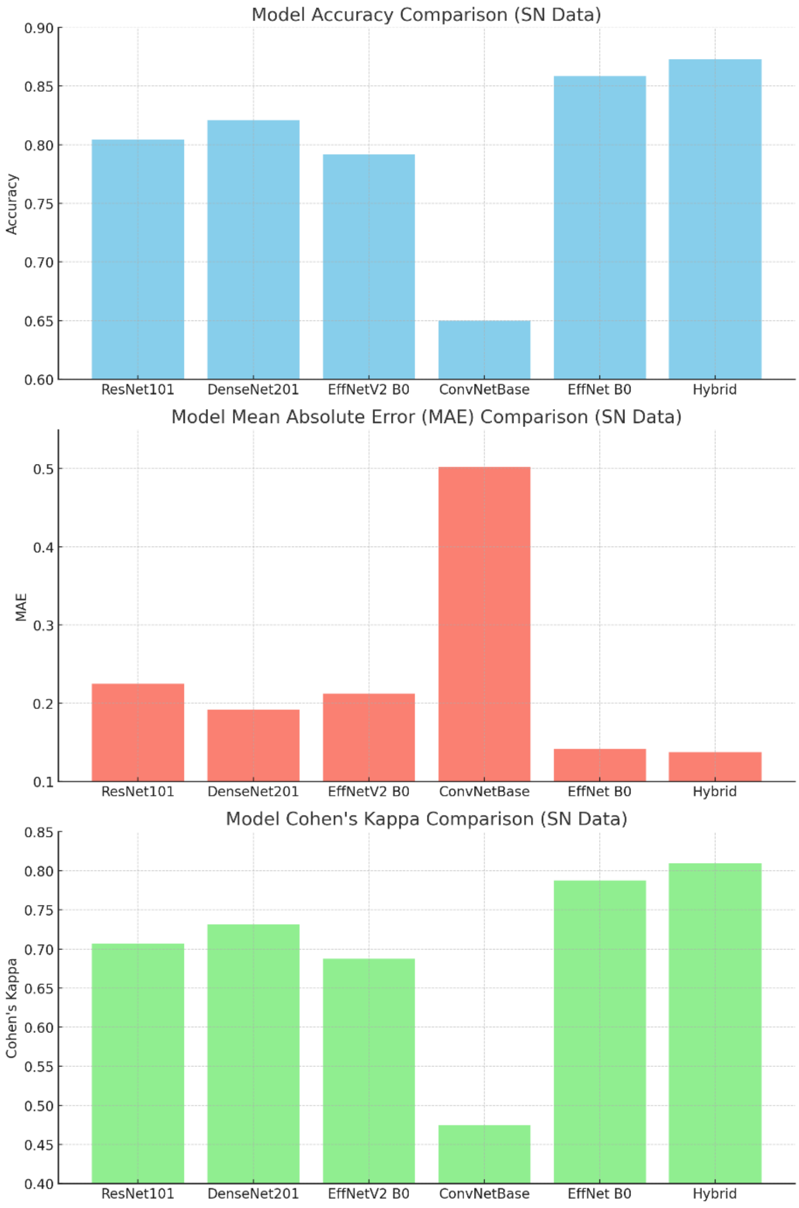

Supplement: Supplementary file 1 [file diagnostics-15-02240-s001.zip › 3 SN-GoGn.pdf]
